# Supplementary material for: ‘It was a joint plan we worked out together’. How the I-WOTCH programme enabled people with chronic non-malignant pain to taper their opioids: a process evaluation
Source: BMJ Open. 2023 Dec 6;13(12):e074603. doi: 10.1136/bmjopen-2023-074603 (PMC10711817; doi:10.1136/bmjopen-2023-074603)
Supplement: Supplementary data [file bmjopen-2023-074603supp001.pdf]

I-WOTCH Process evaluation supplementary material 1-10

Supplementary material 1 Logic Model

The problem	Intervention Aims	Intervention	Theory and Guidance	Interim Targets	Desired Outcomes
People with chronic non-malignant pain are taking opioids, which have side effects and are not effective in the long term.	To test the effectiveness and cost effectiveness of a patient-centred multicomponent self-management intervention targeting withdrawal of strong opioids on activities of daily living for people living with chronic non-malignant pain	**<u>Manualised Intervention Delivery</u>**<br>**Core pain management topics:**<br>• Acute versus Chronic pain<br>• Acceptance<br>• Attention Control and distraction<br>• the pain cycle<br>• Posture and movement advice<br>• Relaxation techniques<br>• Stress busting for health action planning, problem solving, pacing, SMART goal setting<br>• identifying and overcoming barriers to change<br>• Mindfulness<br>• Anger, irritability and frustration<br>• Communication Skills<br><br>**Core opioid specific topics:**<br>• The rationale of prescribing in chronic pain<br>• Opioid induced tolerance and need for dose escalation<br>• Evidence of usefulness of opioids short and long term<br>• Side effects of opioids short term and long term<br>• Case studies of successful discontinued opioid therapy<br>• Opioid withdrawal symptoms	Theory of Planned Behaviour<br><br>Social Cognitive Theory<br><br>Information Motivation and Behavioural (IMB model) skills<br><br>Patient Centred Communication<br><br>Motivational Interviewing	**<u>Staff Training</u>**<br>To facilitate groups, deliver individual tapering consultations and telephone support in an inclusive and non-judgemental manner<br>**<u>Individual participant changes:</u>**<br><u>a Knowledge of:</u> opioids, withdrawal effects, chronic pain<br><u>b Fostering change:</u> self-validation, legitimising pain, normalising expectations<br><u>c Motivation to change by:</u> Improved self-efficacy, effective tapering<br><u>d Skills:</u><br>• <u>General Self-Regulation</u><br>*<u>Psychological skills</u>*<br>Identify reasons for negative emotions (anger /frustration /irritable)<br>Identify problems and solutions, barriers to change<br>Recognise errors in thinking/automatic thoughts<br>Goal setting, goal review<br>*<u>Physical skills</u>*<br>Promote body awareness, posture<br>Reduce muscle tension<br>Body awareness and core strength<br>Relaxation-contract relax<br>• <u>Pain Self-Regulation</u><br>Understand that pain and mood are linked – when is pain bearable and when not bearable.<br>Understanding of pain cycle, unhelpful emotions and behaviours<br>Using mind to relieve pain does not mean pain in mind<br>Distraction whilst relaxed<br>Focus mind away from pain	**<u>Primary outcomes:</u>**<br>Patient-Reported Outcomes Measurement Information System (PROMIS) Pain Interference Short Form (8A)(PROMIS -PI-SF-8A)<br><br>Daily morphine equivalent opioid dose

		• Advantages of slow supervised tapering<br>• Symptom management during tapering<br>• Pain control after opioids		Mindfulness for pain<br>Managing flare ups<br>Need for stretching<br>• Communication Skills<br>How to communicate with General Practitioners (GPs) and Health Care Professionals (HCPs)<br>Listening skills - Active and giving feedback in communication-reward for help.	

Supplementary material 2 I-WOTCH Indicative Interview Topic Guide

Patient Participants

What did you hope to achieve by participating in this research?

Do you want to reduce your opioid intake- and if so why?

How important is it to you to reduce your opioid intake (that is, how strongly motivated are you)?

What were your expectations about whether this would work for you once you heard which group you would be in? (Intervention [I-WOTCH]) /best usual care [my opioid manager plus relaxation package])

How did your expectations match your actual experience?

How did you find using I-WOTCH/my opioid manager/relaxation package? (Where any parts easy for you? Were any parts difficult for you? Why was this?)

Did you find you were you able to use all the different components or not? (Prompt list of the components of intervention/control). If not, which components did you use? Why was this? Were there components you rarely or never used? Why was this?

What was it like trying to use this in your everyday life? (barriers/enablers)

Intervention group only: I'd like to ask you about your experiences of being in a group – how was that for you? (What was good about it, what was less good about it?)

Following from above…. How did the group work, was it better than having one-to-one sessions? Why?

How well was the group facilitated?

If you could change three things about the intervention what would those be?

If another patient asked you about taking part in this programme, what would you say?

Is there anything else you'd like to say that is important to you that I haven't asked you?

Supplementary material 3 Random sampling chart

	Group Number																													
	**1**	**2**	**3**	**4**	**5**	**6**	**7**	**8**	**9**	**10**	**11**	**12**	**13**	**14**	**15**	**16**	**17**	**18**	**19**	**20**	**21**	**22**	**23**	**24**	**25**	**26**	**27**	**28**	**29**	**30**
Sample 1		2		1		3					2			1			3				2		1	3						

All of the intervention groups were given a chronological number. To identify which groups would be checked for fidelity, for each block of ten groups (representing early, mid and later stages of the study) the trial statistician randomly allocated a day 1, day 2 and day 3 session.

Supplementary material 4 Fidelity data collection checklist (sessions in bold assessed for fidelity)

	***Educational and/or self-management regarding pain or opioid use**	Practical, reflection or summarising sessions (not assessed for fidelity, recorded if took place or not)
Day 1	**2, 3, 4, 7, 8**	1,5,6,9,10,11
Day 2	**13, 14, 16**	12,15,17,18,19
Day 3	**21, 22, 23**	20,22 part 2, 24,25,26,27,28

**DAY 1**
Session 1 Introduction
***Session 2 Pain information**
***Session 3 Painkiller information and opioid education**
***Session 4 Acceptance: John's story**
Session 5 Attention Control and distraction
Session 6 Distraction activity – rose drawing
***Session 7 Good days, Bad days when is pain bearable and when is it not?**
***Session 8 The pain cycle unhelpful emotions and behaviours**
Session 9 Posture
Session 10 Relaxation and Breathing
Session 11 Summary of the day

**DAY 2**
Session 12 Reflections from day 1
***Session 13 Stress-busting – prioritising what's important, action planning, goal setting and pacing**
***Session 14 Withdrawal symptoms, case studies (Opioid Education 2)**
Session 15 Distraction activity- origami
***Session 16 Identifying and overcoming Barriers to change Part 1 – recognising unhelpful thinking**
***Identifying and overcoming Barriers to change Part 2– reframing negatives to positives**
Session 17 Mindful attention control
Session 18 Balance and introduction to stretch
Session 19 Summary of the Day

**DAY 3**
Session 20 Reflections from day 2 and previous week
***Session 21 Anger, irritability and frustration**
***Session 22 Relationships Part 1 Getting the most from your healthcare team**
Session 22 Part 2 Relationships Part 2 Listening skills
***Session 23 Managing setbacks and non-drug management techniques**
Session 24 Distraction activity – mindfulness colouring
Session 25 Stretching muscles that commonly get tight
Session 26 Mindfulness of thoughts and Senses
Session 27 Summary of Day 3
Session 28 Summary of the course

Supplementary material 5 Examples of I-WOTCH fidelity sessional and nurse consultation score sheets for adherence and competence

Day 1 /Session 2 /Title: Pain Information 30mins

Adherence: of the delivery as per protocol

Instructions: When at all possible please rate as 'Yes' or 'No' If 'partially' then write reason in comments box. Questions need not be verbatim (unless specified) as long as content of session is covered.

No.	Item	Adherence	Comments
Intro	Did the facilitator(s) introduce the session?	Yes (2) Partially (1) No (0)	
Step 1	Did the facilitator(s) play the DVD of the biomedical explanation about acute and chronic pain?	Yes (2) Partially (1) No (0)	
	Did the facilitator(s) ask the group Q1 and discuss, "What do you think about this explanation of pain? Is it missing anything?"	Yes (2) Partially (1) No (0)	
Step 2	Did the facilitator(s) present the bio-psycho-social explanation of pain?	Yes (2) Partially (1) No (0)	
	Did the facilitator(s) ask the group Q2 and discuss, "What do you think about this explanation of pain?"	Yes (2) Partially (1) No (0)	
Step 3	Did the facilitator(s) play the DVD of Experiences of living with opioid- treated long term pain?	Yes (2) Partially (1) No (0)	
	Did the facilitator(s) ask the group Q3 and discuss, "What do you think about Caroline's description of living with opioid-treated long-term pain?"	Yes (2) Partially (1) No (0)	
Summary	Did the facilitator(s) consolidate/embed the group's learning at the end of the session? *e.g. reading the summary, putting the session in context*	Yes (2) Partially (1) No (0)	
	Total adherence score (max 16)		
	Percentage adherence score<br>(Total adherence score */16x100)		
**Comments:** For use if sessions; go off track, include items which are not on checklist, contain surprising unforeseen aspects or the facilitation wasn't covered as intended. Also if there was no opportunity to demonstrate the skill listed.			

Day 1 /Session 2 /Title: Pain Information and Opioid Education

<u>Competence:</u> of the quality of delivery or 'skill' of the facilitators

	Item	Competence measure	Comments (*use box below to expand)*
1	Did the facilitator(s) create opportunities for discussion *e.g. did they; encourage individuals to participate, ask open questions, give enough time for the group to answer (rather than answer their own questions)*	Evident (2)<br>Partially evident (1)<br>Not evident (0)<br>Did not happen in this session (N/A)	
2	Did the facilitator(s) encourage individual disclosure? *e.g. did they ask different group members to comment or encourage the group to explore issues further (either individually or as a group)?*	Evident (2)<br>Partially evident (1)<br>Not evident (0)<br>Did not happen in this session (N/A)	
3	Did the facilitator(s) validate participants' disclosures? *e.g. Do other people find this/think that? I know how you feel. Sometimes people may feel differently about things.*	Evident (2)<br>Partially evident (1)<br>Not evident (0)<br>Did not happen in this session (N/A)	
4	Did the facilitator(s) give encouraging feedback on participants reported behaviours? *e.g. Did they give appraisal 'that's really good' or 'that's really good but I wonder if…'*	Evident (2)<br>Partially evident (1)<br>Not evident (0)<br>Did not happen in this session (N/A)	
5	Did the facilitator(s) foster a positive group climate? *e.g. did they; use humour, say positive things about people 'that's a helpful comment' 'thank you for sharing that'*	Evident (2)<br>Partially evident (1)<br>Not evident (0)<br>Did not happen in this session (N/A)	
6	Did the facilitator acknowledge and respond appropriately to admissions or statements of low self-efficacy? e.*g. 'yes this can be difficult but…' ideas or examples offered of how this may be done. Issues surrounding confidence.*	Evident (2)<br>Partially evident (1)<br>Not evident (0)<br>Did not happen in this session (N/A)	
7	Did the facilitator respond appropriately to disclosures of negative events or barriers to progress?	Evident (2)<br>Partially evident (1)<br>Not evident (0)<br>Did not happen in this session (N/A)	
	Total competence score (max 14)		
	Percentage competence score		

Comments: For use if sessions; go off track, include items which are not on checklist, contain surprising unforeseen aspects or the facilitation wasn't covered as intended. Also if there was no opportunity to demonstrate the skill listed.

Fidelity score sheet for first one to one nurse consultation

Item	Adherence	Score	Comments *(expand in box below)*
1	Was the participant asked about their thoughts/feelings on reducing their opioids?	Yes (2)<br>Partially (1)<br>No (0)	
2	Was the participant asked about their pain related medication usage?	Yes (2)<br>Partially (1)<br>No (0)	
3	Was the participant's pain discussed?	Yes (2)<br>Partially (1)<br>No (0)	
4	Was a tapering plan discussed/negotiated?	Yes (2)<br>Partially (1)<br>No (0)<br>Not evident	
5	Were temporary withdrawal side effects mentioned?	Yes (2)<br>Partially (1)<br>No (0)	
6	Was a tapering plan summarised and a copy given to the participant?	Yes(2)<br>Partially (1)<br>No (0)	
7	Were barriers to implementing the tapering plan and setbacks discussed?	Yes (2)<br>Partially (1)<br>No (0)<br>Not evident	
8	Did the nurse leave an opportunity for any questions?	Yes (2)<br>Partially (1)<br>No (0)	
9	Did the nurse ensure the participant knew to make an appointment with the GP?	Yes (2)<br>Partially (1)<br>No (0)	
	Total score out of 14 or 18 max		
	Percentage score total score/ 14 or 18 x 100		
Comments:			

Item	Competence	Score	Comments *(expand in box below if nec.)*

1	Did the nurse allow participants to express their concerns/achievements?	Evident (2)<br>Partially evident (1)<br>Not evident (0)	
2	Did the nurse allow participants to discuss and/or explore their concerns/achievements?	Evident (2)<br>Partially evident (1)<br>Not evident (0)	
3	Did the nurse demonstrate empathy? e.g. *Did they show they understood the participant's feelings?*	Evident (2)<br>Partially evident (1)<br>Not evident (0)	
4	Did the nurse accept the participant's perspective? e.*g. Did they allow exploration of positive and negative feelings about pain and/or opioids, were they non-judgmental?*	Evident (2)<br>Partially evident (1)<br>Not evident (0)	
5	Did the nurse actively listen to the participant? e.*g. Did they use 'uhuu', 'oh' 'um' 'really' type of phrases to demonstrate they were listening*	Evident (2)<br>Partially evident (1)<br>Not evident (0)	
6	Did they support self-efficacy? *e.g. Did they offer reassurance, suggest other techniques, congratulate them on any successes or small steps in the right direction.*	Evident (2)<br>Partially evident (1)<br>Not evident (0)	
	Total score out of 12 max		
	Percentage score total score/ 12 x 100		
Comments:			

Supplementary material 6 Change Mechanism Questions

Motivation, expectation, self-efficacy and perceived intervention efficacy questions

Baseline motivation (baseline and follow-up).

I want to reduce my opioid use.

(Not at all, by a little, by half, so I only use a little, so I use no opioids)

Baseline expectation (baseline only).

I expect that, in 4 months' time, I will have reduced my opioid use.

(Not at all, by a little, by half, so I only use a little, so I use no opioids)

Baseline self-efficacy (baseline only).

I am confident I could reduce my opioid use a lot over 4 months.

(Not at all confident, somewhat confident, fairly confident, strongly confident, completely confident)

Perceived intervention efficacy (baseline and follow-up).

Baseline

I feel that involvement in this study can help me to reduce my opioid use.

(Not at all, by a little, by half, so I only use a little, so I use no opioids)

Follow-up

I feel that involvement in this study has helped me to reduce my opioid use.

(Not at all, by a little, by half, so I only use a little, so I use no opioids)

Supplementary material 7 Interviewee characteristics and uptake for the interview study

**Interviewee ID**	**Location cohort ID** NE (Northeast) orblank(midlands)	**Gender**	**Age decade**	**Opioid usage at 12m compared to baseline***	**Opioid Type**	**Baseline Morphine Equivalent (ME)** **	**Allocation*****
Pilot 1	P1	F	70's	0	Di hydrocodeine / morphine	30-59	Int
3	1	F	30's	S	Tramadol MR	0-29	Intention to treat
4	1	F	50's	L by half	Tramadol	0-29	UC
5	2	M	60's	0	Buprenorphine patches	60-89	Int
6	3	M	60's	H	Tramadol and cocodamol	0-29	UC
7	3	F	50's	L sl	Buprenorphine patches	30-59	Int
8	5 NE	F	60's	0	Zomorph(morphine)	30-59	UC
9	5 NE	M	60's	0	Tramadol	0-29	Int
10	4 NE	F	50's	0	Fentanyl patches	30-59	Int
11	6	F	40's	S	Morphine SR and Oromorph	150+	UC
12	7 NE	F	50's	L	Oxycodone and liquid morphine	90-119	UC
13	12 NE	F	50's	0	Tramadol	0-29	Int
14	9 NE	M	60's	S	Tramadol	0-29	Int
15	11	F	70's	H *(?)*	Tramadol	0-29	UC
16	11	M	60's	H	Oxycodone	30-59	Int
17	10	M	70's	S	Tramadol	0-29	UC
18	8	F	80's	0	Morphine	0-29	UC
19	17 NE	M	60's	L sl.	Tapentadol /morphine/ liquid morphine	60-89	UC
20	9 NE	M	60's	L	Morphine / Codeine phosphate / Tramadol	60-89	UC
21	15	F	60's	L sl	Morphine / liquid morphine	150+	Int
22	8	M	50's	H	Tramadol / Morphine	60-89	Int

23	13	M	70's	H	Liquid morphine and Codeine phosphate	0-29	UC
24	16	M	40/*50*	(S) Still on	Oxycodone changed to fentanyl unknown 12m ME	90-119	UC
25	16	F	80	?L	Buprenorphine patches	30-59	Int
26	13	M	70	L	Morphine + ?	60-89	Int
27	19	F	50's	H	Fentanyl patches	150+	UC
28	18 NE	F	70s	H	Morphine + liquid morphine	150+	UC
29	17 NE	F	50/60	L	Codeine phosphate and fentanyl patches	150+	Int
30	22 NE	M	60/70	L	Tramadol	0-29	Int
31	20	F	60's	0	Fentanyl patches	90-119	Int
32	21	F	80	L	Oxycodone	60-89	Int
33	21	F	80's	Lower by 2/3	Buprenorphine patch	30-59	UC
34	20	F	60's	H(? S)	Hydromorphone ??	150+??	Int
35	20	M	50's	sl L	Tramadol	30-59	UC
36	26 NE	M	70's	L (or 0)	??Oxycodone and liquid morphine	150+	Int
37	26 NE	F	50s	S	Tramadol	60-89	UC
38	24	F	70's	H sl	Tramadol	0-29	Int
39	27	F	50s	L (or S)	Buprenorphine to Morphine	30-59	UC
40	23 NE	F	60s	0 (or L)	Tramadol to Codeine phosphate	0-29	UC
41	29 NE	F	60s	L sl	Fentanyl patches ??liquid morphine	??	Int

Legend: * H=higher S=Same L=lower 0 = no opioids
**Baseline morphine equivalent bandings: 0-29mg,30-59mg,60-89mg,90-119mg, 120-149mg, 150mg+
***Allocation: UC- Usual Care, Int-Intervention, ITT-Intention to treat (allocated to Intervention but not exposed to intervention)

Comparison of Usual care and Intervention

Usual care Total 20	7NE 13 Mids	7M 13F	30s to 80s	5H 5S 7L 30	Range of patches tablets and liquid	0-29(8),30-59(4),60-89(3), 90-119(2), 120-149(0), 150+(3)	UC
Intervention Total 20	8 NE 12 Mids	8M 12F	50s to 80s	4H 1S 9L 60	Range of patches tablets and liquid	0-29(5),30-59(5),60-89(4), 90-119(1),120-149(0),150+(4),1 unknown	Int

Commented [NV1]: Do we need this in?

Supplementary material 8 Baseline demographic characteristics of all randomised participants vs interviewed participants

Demographic characteristics	Total N=608	PE Interviewees N=40
**Age (years)**		
N	608	40
Mean (SD)	61.3 (12.9)	64.7 (11.9)
Median (IQR)	62.3 (53.0 – 70.7)	64.1 (58.5, 71.8)
Missing	0	0
**Gender**		
Male	242 (39.8%)	15 (37.5%)
Female	362 (59.5%)	25 (62.5%)
Other	1 (0.2%)	0 (0%)
Prefer not to say	0 (0.0%)	0 (0%)
Missing	3 (0.5%)	0 (0%)
**Ethnicity**		
White	585 (96.2%)	38 (95.0%)
Black Caribbean	6 (1.0%)	1 (2.5%)
Black African	1 (0.2%)	1 (2.5%)
Black Other	1 (0.2%)	0 (0.0%)
Indian	6 (1.0%)	0 (0.0%)
Pakistani	1 (0.2%)	0 (0.0%)
Bangladeshi	0 (0.0%)	0 (0.0%)
Chinese	0 (0.0%)	0 (0.0%)
Prefer not to say	1 (0.2%)	0 (0.0%)
Other	4 (0.7%)	0 (0.0%)
Missing	3 (0.5%)	0 (0.0%)
**Employment status**		
Employed	132 (21.7%)	6 (15.0%)
Unemployed	14 (2.3%)	0 (0.0%)
At school or full time education	1 (0.2%)	0 (0.0%)
At school or part time education	1 (0.2%)	0 (0.0%)
Unable to work due to long term sickness	154 (25.3%)	13 (32.5%)
Looking after home/family	13 (2.1%)	0 (0.0%)
Retired from paid work	270 (44.4%)	21 (52.5%)
Other	20 (3.3%)	0 (0.0%)
Missing	3 (0.5%)	0 (0.0%)
**Age left full time education**		
Did not receive formal education	2 (0.3%)	0 (0.0%)
Age 12 or less	1 (0.2%)	0 (0.0%)
Age 13 to 16	345 (56.7%)	21 (52.5%)
Age 17 to 19	135 (22.2%)	6 (15.0%)
Age 20 or over	109 (17.9%)	12 (30.0%)
Still in full time education	4 (0.7%)	0 (0.0%)
Other	9 (1.5%)	1 (2.5%)
Missing	3 (0.5%)	0 (0.0%)
**How long have you experience pain**		
Less than 1 year	8 (1.3%)	0 (0.0%)
1-5 years	97 (16.0%)	9 (22.5%)
More than 5 years	500 (82.2%)	31 (77.5%)
Missing	3 (0.5%)	0 (0.0%)
**How long have you been taking opioids for your chronic pain**		
Less than 1 year	29 (4.8%)	0 (0.0%)
1-5 years	211 (34.7%)	20 (50.0%)
More than 5 years	365 (60.0%)	20 (50.0%)
Missing	3 (0.5%)	0 (0.0%)

Supplementary material 9 Participant feedback form findings

Questions	Responses			
**Q1 Were the aims of the course made clear?**	Out of 27 responses (4 missing) : 26 Yes, 1 No			
**Q2 What were the three most useful things on this course?**	**Theme** *26 responses (ranked but not all filled in all three).*	**1st**	**2nd**	**3rd**
	Appreciation of the information	8	7	3
	Being in a group – meeting and interacting	8	8	2
	Supported by facilitators	3	3	1
	Lay facilitator input	3	1	0
	Techniques taught in course which were helpful (all different)	3	3	0
	Motivational aspects	1	0	4
	Support of GP	0	0	3
**Q3 What three things would you suggest to make this course better for future participants?**	Out of 24 responses, six suggested to keep it as it is. The overall suggestions from the remainder were disparate. Some wanted more time for discussion (but one wanted shorter sessions due to comfort), more contacts with staff, more meetings especially at end of entire course. Practical issues specific to venue e.g. closeness, parking, one toilet. Two suggested CDs should also be in MP3/4 format. Specific issues: One felt the App advice was unrealistic, one didn't like the meditation, one didn't like the self-help booklet manual, one hadn't used CDs.			

	Very confident	Confident	Not very confident	Not confident at all
**Q4: How confident do you feel that the course content will help you personally?**	14	11	1	4
**Q5: How confident do you feel that you will be able to use this in the future?**	16	10	0	1
	**Very good**	**Good**	**Satisfactory**	**Poor**
**Q6: Overall were the facilitators?**	23	4	1	0
**Q7: Overall were the handouts?**	15	12	1	0
	**Very useful**	**Useful**	**Not very useful**	**Not useful at all**
**Q8: How did you find the face to face meeting with the nurse?**	22	5	1	0
**Q9: How did you find the telephone calls with the nurse?**	18	9	1	0
**Q10: Overall how useful did you find the whole course?**	22	5	0	1

Q11: Is there anything else you'd like to say? 21 responses to an open comments box. Fifteen positive, 2 negative ('course was a waste of time' and that the approach had not worked for them) and three mixed. Indicative quotes:

"I am now completely off tramadol and despite still going through some nasty withdrawal symptoms I am so glad I had the opportunity to attend the course. I didn't realise how bad the drug is and how ineffective it is for long term chronic pain. I am looking forward to getting my life back."

"I would recommend this course to anyone who has a chance to attend it. It has helped me to come off one of my painkillers, to be more outgoing, to talk about my problems more and given me more confidence when in a group of people."

Supplementary material 10 Summary of intervention attendance data

	Self-management (intervention)
**Group session attendance[1,2]**	
Number randomised to intervention	305
Session 1 only	13 (4.3%)
Session 1 and 2	17 (5.6%)
Session 1 and 3	10 (3.3%)
Session 1, 2 & 3	166 (54.4%)
Attended no sessions	90 (29.5%)
Missing	9 (3.0%)
**Group size at randomisation**	
N	35
Mean (SD)	8.71 (2.9)
Median (IQR)	9 (5,11)
Missing	0 (0%)
**Group size at Session 1[3]**	
N	35
Mean (SD)	6.24 (2.82)
Median (IQR)	7 (3, 8)
Missing	2 (5.7%)
**Face to Face interviews**	
Attended first F2F interview	190 (62.3%)
Attended both F2F interviews	131 (42.9%)
Missing	15 (4.9%)
**Telephone interviews**	
Attended first telephone session	167 (54.8%)
Attended both telephone sessions	152 (49.8%)
Missing	34 (11.1%)
**Compliance**	
Number of participants with full compliance	144 (47%)
Number of participants with at least minimal compliance	190 (62%)
Number with less than minimal compliance	115 (38%)

Legend: [1] 161/305 participants achieved minimal compliance by attending at least Day 1 and the first one-to-one consultation.
[2] 144/305 participants achieved full compliance by attending at least Day 1, 2 & 3, the first one-to-one consultation and 1 telephone call.
[3] 6 participants who attended day 1 attended groups they were not randomised to. This has been summarised by groups they attended, not randomised too.

Supplementary material 11 Fidelity scores of group sessions in percentages

Session	Adherence *(italics 10% check)*			Totals	Competence			Totals
	**Early**	**Mid**	**Late**		**Early**	**Mid**	**Late**	
Day 1 session 2	100	81	25 *(25)*		92	64	0 *(0)*	
Day 1 session 3	94 *(94)*	100	44		90 *(87)* agreed 88	86	42	
Day 1 session 4	100	75	88		70	71	67	
Day1 session 7	72	89	83		80	75	90	
Day 1 session 8	100	88	81		100	60	75	
Day 2 session 13	77	88 *(90)* agreed 89	84		57	100 *(100)*	100	
Day 2 session 14	56 *(66)* agreed 61	100	94		58*(43)* agreed 50	92	90	
Day 2 session 16	79	88	82		50	70	100	
Day 3 session 21	88	100	89		80	100	100	
Day 3 session 22pt 1	93	86	64		70	92	83	
Day3 session 23	90	73	91		100	100	100	
**Average**	**86.72**	**88.09**	**75.00**	**83.27%**	**76.09**	**82.73**	**77**	**78.61%**
**Range**	**61-100**	**73-100**	**25-94**	**25-100%**	**50-100**	**60-100**	**0-100**	**0-100%**
**Median**	**90**	**88**	**83**	**88**	**80**	**86**	**90**	**86**

Supplementary material 12 Fidelity scores of one-to-one nurse consultations

Timepoint Early	Group ID	Adherence Score	Competence Score
1st	1	100%	100%
2nd	2	93% *(93%)*	100% (*100%*)
1st	3	61%	67%
2nd	3	86%	83%
1st	4 NE	100%	100%
2nd	4 NE	100%	100%
1st	5 NE	100%	100%
2nd	5 NE	100%	100%
2nd	6	100%	100%
1st	7 NE	67%	100%
1st	8	100%	100%
1st	9 NE	83% *(94%)*94	100% *(83%)*92
2nd	10	88%	83%
2nd	10	100%	100%
**Early averages**		**92.07%**	**94.64%**
**Range**		**61 to 100**	**67 to 100**
**Timepoint Mid**	**Group ID**	**Adherence**	**Competence**
1st	11	100%	100%
1st	12 NE	94%	100%
2nd	12 NE	100%	100%
1st	13	89%	92%
1st	14 NE	61% *(61%)*	50% *(50%)*
1st	15	94%	100%
1st	15	94%	100%
2nd	20	71%	83%
**Mid averages**		**87.87%**	**90.63%**
**Range**		**61 to 100**	**50 to 100**
**Timepoint Late**	**Group ID**	**Adherence Score**	**Competence Score**
1st	21	100%	92%
2nd	21	93%	100%
1st	24	94%	100%
2nd	24	86% *(86%)*	100% *(100%)*
1st	27	89%	83%
**Late averages**		**92.4%**	**95%**
**Range**		**86 to 100**	**83 to 100**
	**Total averages**	**90.78%**	**93.42%**
	**Range**	**61 to 100**	**50 to 100**
**Legend**: NE - North East region			

Supplementary material 13

Baseline change mechanism questions of all randomised participants by treatment group

		Control N=303	Intervention N=305	TOTAL N=608
**1.I want to reduce my opioid use**				
Motivation	Not at all	25 (8%)	21 (7%)	46 (8%)
	By a little	45 (15%)	37 (12%)	82 (14%)
	By Half	36 (12%)	44 (14%)	80 (13%)
	So I only use a little	60 (20%)	95 (31%)	155 (26%)
	So I use no opioids	133 (44%)	102 (33%)	235 (39%)
	Missing	4 (1%)	6 (2%)	10 (2%)
**2.I expect in 4 months' time, I will have reduced my opioid use**				
Expectations	Not at all	45 (15%)	43 (14%)	88 (15%)
	By a little	78 (26%)	82 (27%)	160 (26%)
	By Half	56 (19%)	56 (18%)	112 (18%)
	So I only use a little	67 (22%)	82 (27%)	149 (25%)
	So I use no opioids	50 (17%)	37 (12%)	87 (14%)
	Missing	7 (2%)	5 (2%)	12 (2%)
**3.I am confident I could reduce my opioid use a lot over 4 months**				
Self-efficacy	Not at all confident	90 (30%)	90 (30%)	180 (30%)
	Somewhat confident	70 (23%)	77 (25%)	147 (24%)
	Fairly confident	79 (26%)	79 (26%)	158 (26%)
	Strongly confident	35 (12%)	40 (13%)	75 (12%)
	Completely confident	22 (7%)	15 (5%)	37 (6%)
	Missing	7 (2%)	4 (1%)	11 (2%)
**4.I feel that involvement in this study can help me to reduce my opioid use**				
Perceived credibility of intervention	Not at all	25 (8%)	22 (7%)	47 (8%)
	By a little	76 (25%)	73 (24%)	149 (25%)
	By Half	38 (13%)	46 (15%)	84 (14%)
	So I only use a little	68 (22%)	86 (28%)	154 (25%)
	So I use no opioids	86 (28%)	69 (23%)	155 (26%)
	Missing	10 (3%)	9 (3%)	19 (3%)

Supplementary material 14

Change mechanism question data from 4, 8 and 12 month follow up

**4-month follow-up change mechanism questions of all randomised participants by treatment group**	**Control**	**Intervention**
**I want to reduce my opioid use (If still on opioids)**		
Number still on opioids	194	166
Not at all	20 (10.3%)	18 (10.8%)
By a little	23 (11.9%)	11 (6.6%)
By Half	32 (16.5%)	11 (6.6%)
So I only use a little	29 (14.9%)	31 (18.7%)
So I use no opioids	40 (20.6%)	57 (34.3%)
Missing	50 (25.8%)	38 (22.9%)
**I feel that involvement in this study has helped me to reduce my opioid use**		
Number still on opioids	159	192
Not at all	82 (51.6%)	30 (15.6%)
By a little	43 (27.0%)	24 (12.5%)
By Half	10 (6.3%)	21 (10.9%)
So I only use a little	6 (3.8%)	33 (17.2%)
So I use no opioids	8 (5.0%)	79 (41.1%)
Missing	10 (6.3%)	5 (2.6%)
**8-month follow-up change mechanism questions of all randomised participants by treatment group**	**Control**	**Intervention**
**I want to reduce my opioid use (If still on opioids)**		
Number still on opioids	152	136
Not at all	25 (16.4%)	20 (14.7%)
By a little	25 (16.4%)	16 (11.8%)
By Half	24 (15.8%)	9 (6.6%)
So I only use a little	25 (16.4%)	26 (19.1%)
So I use no opioids	36 (23.7%)	45 (33.1%)
Missing	17 (11.2%)	20 (14.7%)
**I feel that involvement in this study has helped me to reduce my opioid use**		
N	149	181
Not at all	68 (45.6%)	27 (14.9%)
By a little	41 (27.5%)	27 (14.9%)
By Half	16 (10.7%)	20 (11.0%)
So I only use a little	9 (6.0%)	28 (15.5%)
So I use no opioids	12 (8.1%)	74 (40.9%)
Missing	3 (2.0%)	5 (2.8%)
**12-month follow-up change mechanism questions of all randomised participants by treatment group**	**Control**	**Intervention**
**I want to reduce my opioid use (If still on opioids)**		
Number still on opioids	193	160
Not at all	20 (10.4%)	18 (11.3%)
By a little	22 (11.4%)	10 (6.3%)
By Half	27 (14.0%)	15 (9.4%)
So I only use a little	36 (18.7%)	32 (20.0%)
So I use no opioids	38 (19.7%)	49 (30.6%)
Missing	50 (25.9%)	36 (22.5%)
**I feel that involvement in this study has helped me to reduce my opioid use**		
N	160	188
Not at all	73 (45.6%)	26 (13.8%)
By a little	39 (24.4%)	25 (13.3%)
By Half	17 (10.6%)	18 (9.6%)
So I only use a little	10 (6.3%)	44 (23.4%)

So I use no opioids	18 (11.3%)	72 (38.3%)
Missing	3 (1.9%)	3 (1.6%)

Supplementary material 15

Involvement in study reducing opioid use at 4, 8 and 12 months allocation comparison

**I feel that involvement in this study has helped me to reduce my opioid use:** Not at all, by a little, by half, so I only use a little, so I use no opioids						
**Grouping into 3 bands**	**Control 4month follow up**	**Intervention 4month follow up**	**Control 8month follow up**	**Intervention 8month follow up**	**Control 12month follow up**	**Intervention 12month follow-up**
N	159	192	149	181	160	188
Opioids reduced not at all + by a little	125 (79%)	54 (28%)	109 (73%)	54 (30%)	112 (70%)	51 (26%)
By half	10 (6%)	21 (11%)	16 (11%)	20 (11%)	17 (11%)	18 (10%)
Opioids reduced so I only use a little + so I use none	14 (9%)	112 (58%)	21 (14%)	102 (56%)	28 (18%)	116 (62%)
Missing	10 (6%)	5 (3%)	3 (2%)	5 (3%)	3 (2%)	3 (2%)
