# Supplementary material for: ‘It was a joint plan we worked out together’. How the I-WOTCH programme enabled people with chronic non-malignant pain to taper their opioids: a process evaluation
Source: BMJ Open. 2023 Dec 6;13(12):e074603. doi: 10.1136/bmjopen-2023-074603 (PMC10711817; doi:10.1136/bmjopen-2023-074603)
Supplement: Author's manuscript [file bmjopen-2023-074603.draft_revisions.pdf]

BMJ Open is committed to open peer review. As part of this commitment we make the peer review history of every article we publish publicly available.

When an article is published we post the peer reviewers' comments and the authors' responses online. We also post the versions of the paper that were used during peer review. These are the versions that the peer review comments apply to.

The versions of the paper that follow are the versions that were submitted during the peer review process. They are not the versions of record or the final published versions. They should not be cited or distributed as the published version of this manuscript.

BMJ Open is an open access journal and the full, final, typeset and author-corrected version of record of the manuscript is available on our site with no access controls, subscription charges or pay-per-view fees (http://bmjopen.bmj.com).

If you have any questions on BMJ Open's open peer review process please email info.bmjopen@bmj.com

BMJ Open

"It was a joint plan we worked out together." How the I-WOTCH programme enabled people with chronic non-malignant pain to taper their opioids: a process evaluation

Journal:	*BMJ Open*
Manuscript ID	bmjopen-2023-074603
Article Type:	Original research
Date Submitted by the Author:	20-Apr-2023
Complete List of Authors:	Nichols, Vivien; University of Warwick, Warwick Clinical Trials Unit<br>Abraham, Charles; Deakin University<br>Eldabe, Sam; The James Cook University Hospital<br>Sandhu, Harbinder; University of Warwick Warwick Medical School, Clinical Trials Unit<br>Underwood, Martin; Warwick University, Warwick Medical School<br>Seers, Kate; University of Warwick, Warwick Medical School
Keywords:	Chronic Pain, PAIN MANAGEMENT, QUALITATIVE RESEARCH, Patient-Centered Care

SCHOLARONE™
Manuscripts

I, the Submitting Author has the right to grant and does grant on behalf of all authors of the Work (as defined in the below author licence), an exclusive licence and/or a non-exclusive licence for contributions from authors who are: i) UK Crown employees; ii) where BMJ has agreed a CC-BY licence shall apply, and/or iii) in accordance with the terms applicable for US Federal Government officers or employees acting as part of their official duties; on a worldwide, perpetual, irrevocable, royalty-free basis to BMJ Publishing Group Ltd ("BMJ") its licensees and where the relevant Journal is co-owned by BMJ to the co-owners of the Journal, to publish the Work in this journal and any other BMJ products and to exploit all rights, as set out in our licence.

The Submitting Author accepts and understands that any supply made under these terms is made by BMJ to the Submitting Author unless you are acting as an employee on behalf of your employer or a postgraduate student of an affiliated institution which is paying any applicable article publishing charge ("APC") for Open Access articles. Where the Submitting Author wishes to make the Work available on an Open Access basis (and intends to pay the relevant APC), the terms of reuse of such Open Access shall be governed by a Creative Commons licence – details of these licences and which Creative Commons licence will apply to this Work are set out in our licence referred to above.

Other than as permitted in any relevant BMJ Author's Self Archiving Policies, I confirm this Work has not been accepted for publication elsewhere, is not being considered for publication elsewhere and does not duplicate material already published. I confirm all authors consent to publication of this Work and authorise the granting of this licence.

For peer review only

"IT WAS A JOINT PLAN WE WORKED OUT TOGETHER." HOW THE I-WOTCH PROGRAMME ENABLED PEOPLE WITH CHRONIC NON-MALIGNANT PAIN TO TAPER THEIR OPIOIDS: A PROCESS EVALUATION

Authors

Vivien P Nichols
Warwick Clinical Trials Unit,
Warwick Medical School,
University of Warwick, Coventry, UK

Charles A Abraham
Professor of Psychology
School of Psychology, Deakin University, Geelong.
Australia

Sam Eldabe
Department of Pain Medicine,
The James Cook University
Hospital, Middlesbrough, UK

Harbinder K Sandhu
Warwick Clinical Trials Unit,
Warwick Medical School,
University of Warwick, Coventry, UK

Martin Underwood
Warwick Clinical Trials Unit,
Warwick Medical School,
University of Warwick, Coventry, UK
University Hospitals of Coventry and Warwickshire
Coventry, UK

Kate Seers corresponding author
Warwick Research in Nursing,
Division of Health Sciences,
Warwick Medical School,
University of Warwick, Coventry, UK
Kate.Seers@warwick.ac.uk

On behalf of the I-WOTCH team see acknowledgements

For peer review only - http://bmjopen.bmj.com/site/about/guidelines.xhtml

Abstract

Background

The I-WOTCH randomised controlled trial found that a group based educational intervention to support people using strong opioids for chronic non-malignant pain helped a significant proportion of people to stop or decrease opioid use with no increase in pain-related disability. We report a linked process evaluation of the group-based intervention evaluated in comparison to a usual-care control group who received a self-help booklet and relaxation CD.

Methods

We interviewed 18 intervention facilitators, and 20 intervention and 20 control participants who had chronic non-malignant pain and were recruited from general (family) practices in the UK. Quantitative data included change-mechanism questions on the trial questionnaires which explored motivation, expectations, and self-efficacy. Fidelity was assessed by listening to a sample of audio-recorded group sessions and nurse consultations. Quantitative and qualitative data were integrated using 'follow a thread' and a mixed methods matrix.

Findings

Four overarching themes emerged: (i) the right time to taper, (ii) the backdrop of a life with chronic pain, (iii) needing support and (iv) the benefits of being in a group. Delivery fidelity was good, adherence (83%) and competence (79%) across a range of intervention groups. Staff delivering the intervention found three typical responses to the intervention: resistance, open to trying, and feeling it was not the right time. The group experience was important to those in the intervention arm. It provided people with a forum in which to learn about the current thinking about opioid usage and its effects. It also gave them examples of how feasible or personally relevant coming off opioids might be.

Conclusion

The process evaluation data showed that the I-WOTCH intervention was well delivered, well received and useful for most interviewees. Being "the right time" to taper and having support throughout tapering, emerged as important factors within the context of living with chronic pain.

For peer review only - http://bmjopen.bmj.com/site/about/guidelines.xhtml

Key words: Opioid, process evaluation, chronic non-malignant pain, pain self-management, tapering, behaviour change, complex intervention.

For peer review only

For peer review only - http://bmjopen.bmj.com/site/about/guidelines.xhtml

Strengths and Limitations

- The success of I-WOTCH was demonstrated from multiple perspectives providing an insight into the acceptability, replicability, and transferability of the intervention.
- Enablers of, and barriers to, the tapering process were explored, both of which are valuable for tailoring of this type of intervention to the individual.
- This protocol-based process evaluation showed good fidelity of delivery and a very positive experience of the intervention, suggesting its suitability for service roll out.
- General practitioner perspectives were not a part of this process evaluation but may have provided useful information for future implementation.
- Our participants had agreed to take part in a tapering trial so they may not be representative of the wider chronic non-malignant pain population.

This work was supported by the National Institute for Health and Care Research (NIHR) Health Technology Assessment (HTA) project number 14/224/04 and given ethics approval by: Yorkshire and the Humber- South Yorkshire Research Ethics Committee on 13 Sept 2016 (16/YH/0325)

Trial registration number: ISRCTN49470934

For peer review only

Background

Embedded process evaluations of complex interventions in randomised controlled trials are critical to increase understanding of how and why interventions may or may not work, as well as how practical interventions are when implemented in service contexts. They increase confidence in trial results and provide guidance on their implementation [1, 2].

In the landmark I-WOTCH (Improving the Wellbeing of people with Opioid Treated CHronic pain) trial (n=608) we demonstrated that a self-management support intervention helps one in five people using strong opioids become opioid free at one year: 29% intervention vs 7% control, odds ratio 6.55 (95% confidence interval 3.42 to 12.55) with no increase in pain interference.

The I-WOTCH intervention included educational, behavioural, and psychological components, combining group and one to one support. Papers reporting the intervention development, the trial protocol, and the main results, and process evaluation protocol have been published [3 4, 5 6]. Here we report the I-WOTCH process evaluation, specifically.

- Experiences of the I-WOTCH intervention(s): including enablers of, and barriers to, change among participants.
- Implementation of the I-WOTCH group intervention: exploring the dose delivered and received, and the fidelity of delivery.
- Change mechanisms potentially underpinning intervention effects.
- Contextual issues: exploring how these may affect the outcome or running of the study and/or intervention.

For peer review only

Methods

We explored experiences of the participants and intervention delivery staff as well as key areas of process evaluation; context (contextual factors which may affect the implementation), fidelity (whether the intervention was delivered as conceived), dose delivered (the amount of the intervention delivered) and dose received (the amount of the intervention received by participants).[7]

The I-WOTCH trial recruited participants with chronic non-malignant pain from general (family) practices from midlands and north-east of England (Table 1). Once randomised all participants received a self-help booklet ‘My Opioid Manager’ and a relaxation CD. The intervention group also received a three-day group intervention, run by a specially trained nurse/other health care professional and lay person with chronic pain who had tapered their own opioids, with two additional face to face nurse consultations and telephone support.

Box 1: I-WOTCH participant inclusion and exclusion criteria

Inclusion criteria	Exclusion criteria
• Provision of written informed consent<br>• Aged 18 years old or above<br>• Using opioids for chronic non-malignant pain<br>• Using strong opioids for at least 3 months<br>• Using strong opioids on most days in the preceding month<br>• Fluent in written and spoken English<br>• Able to attend group sessions<br>• Willing for GP to be informed of participation	• Regular use of injected opioid drugs<br>• Chronic headache as the dominant painful disorder<br>• Serious mental health problems that preclude participation in a group intervention<br>• Previous entry or randomisation in the present trial<br>• Participation in a clinical trial of an investigational medicinal product in the last 90 days<br>• Pregnant at time of eligibility assessment, or actively trying to become pregnant<br>• People receiving strong opioid for the management of pain due to active malignant disease

We used process evaluation theory to devise a logic model specific to this intervention and its theoretical underpinnings, informed by The Information, Motivation and Behaviour Skills Model[8,9] which highlights that knowledge, strong and stable motivation, and prerequisite skills are needed to initiate and maintain behaviour change (supplementary material 1 – Logic Model). We used a mixed methods approach for the process evaluation (Table 1).

For peer review only - http://bmjopen.bmj.com/site/about/guidelines.xhtml

Table 1 I-WOTCH process evaluation: Key components, methods, analyses and aims

Key Components	Data Type	Methods	Analysis	Aims Addressed
**Participant interviews**	Qualitative	Semi-structured, face-to-face at home or convenient location. Post 12-month questionnaire. Purposively sampled to attain a range across; age, opioid reduction experience, location and gender across the intervention and control arms.	Framework Analysis[10]	1) Experiences of being in the I-WOTCH study
**Intervention delivery staff interviews**	Qualitative	Semi-structured, face-to-face at workplace or convenient location or telephone. After all intervention groups were completed Pragmatic sample of Nurse and Lay facilitators– all invited	Framework Analysis[10]	1) Experiences of delivering the I-WOTCH intervention
**Participant feedback forms**	Qualitative & Quantitative	Paper form given to last 10 groups after day 3, asked to complete and send back in trial return prepaid envelope. Open and closed questions.	Thematic Analysis[11]	1) Experiences of attending the group components of the intervention
**Dose delivered**	Quantitative	Trial data describing how many group sessions were conducted in each location.	Descriptive statistics, charts, tables or figures using STATA 17.0. The mean and standard deviation (sd) were presented for continuous data, and the frequency and percentage for categorial data, summarised by treatment arm.	2) Intervention implementation
**Dose received**	Quantitative	Trial data of attendance and attrition. Uptake of the one-to-one consultations and follow up telephone calls. (It was not possible to record telephone conversations).	Descriptive statistics, charts, tables or figures using STATA 17.0. The mean and standard deviation (sd) presented for continuous data, and the frequency and percentage for categorial data, summarised by treatment arm.	2) Intervention implementation
**Fidelity**	Quantitative	All intervention groups and one-to-one nurse consultations audio recorded. A sample of recordings listened to and assessed for adherence and competence.	Descriptive statistics - Adherence and competency scores summed, and percentage score calculated.	2) Intervention implementation
**Change mechanism questions**	Quantitative	Trial data collected from four questions about participant motivation, expectations, self-efficacy and perceived credibility of the intervention in the main trial questionnaire.	Descriptive statistics, charts, tables or figures using STATA 17.0. The mean and standard deviation (sd) presented/ continuous data, and the frequency and percentage / categorial data, summarised by treatment arm.	3) Potential effects of Change mechanisms
**Context**	Qualitative & Quantitative	Contextual factors were considered across all the data	Thematic Analysis	4) Contextual issues
**Synthesis of the data**	Qualitative & Quantitative	Mixed methods approach combining all the data to produce a model of Overarching Themes.	Use of a 'mixed methods matrix' and 'following a thread' analysis strategy. O'Cathain et al[12]	(1,2,3 and 4)

For peer review only - http://bmjopen.bmj.com/site/about/guidelines.xhtml

Interviews

Potential interviewees (participants and staff) were sent an information leaflet. Those interested gave informed consent to be interviewed. Intervention delivery staff were told of the interviews at their training and were approached on completing their final group. Semi-structured interviews were recorded using an encrypted device (OLYMPUS DS-7000 digital voice recorder) see supplementary material 2 for indicative topic guide. Recordings were transcribed verbatim, anonymised, given numerical identifiers to maintain confidentiality and checked for accuracy by the research interviewer (VN). The data were analysed using NVivo 12 software to explore and organise the data. Warwick CTU's lone worker policy, including a risk assessment, was followed for interview visits.

All data were collected and stored in digitally secure locations with restricted access in accordance with the Data Protection Act 2018c12 which is the UK's implementation of the General Data Protection Regulation (GDPR)[13]

Participant Feedback forms

We gave feedback forms to the last 10 groups of participants after completing day three of the intervention. Questions asked about different aspects including how the group intervention was delivered and what they had found personally useful/not useful. See supplementary material 9 in results.

Dose delivered and dose received.

We collated trial data including date, location, and attendance of the intervention groups to determine the dose delivered and received.

Fidelity

Our fidelity assessment is detailed in our protocol paper and supplementary material 3 shows our random sampling.[5] We listened to audio recordings of 11 prespecified, intervention group sessions across days one, two and three (those which were educational and promoted discussion see supplementary material 4) to assess adherence to the manual and competence of delivery. Other more practical sessions were checked for content presence but not rated in terms of delivery competence. We used bespoke assessment forms for group and individual sessions across a range of facilitators. Adherence items were scored as taking place:

For peer review only - http://bmjopen.bmj.com/site/about/guidelines.xhtml

Yes (2), Partially (1) or No (0). Competence items were scored as; Evident (2) Partially evident (1) or Not evident (0) (see supplementary material 5). Scores were summated and averages calculated for adherence and competence of each session.

A random 10% of first and second one to one nurse/patient consultation recordings were also assessed throughout the study. If audio-recordings were missing or inaudible, then the same sessions as those missing would be taken from the next group or one to one consultation in the same area (northeast or midlands). A second researcher (KS) double coded 10% of all the assessments.

Potential Change Mechanisms

Four change mechanism questions about; patient motivations, expectations, self-efficacy, and perceived credibility of the intervention were added to the main trial questionnaire to ascertain if any of these factors had an effect on opioid reduction (Supplementary material 6).

The Process Evaluation team

The process evaluation team included; KS a co-applicant and work package lead who has a long track record in qualitative and mixed methods research in complex interventions, CA a co-applicant, highly experienced in behaviour change research and process evaluations and VN a research fellow with a background in physiotherapy, experienced in mixed methods in rehabilitation RCTs. The team collated data and analysed it separately from the main study. The initial report was given to the senior and chief investigators SE, HS and MU prior to the results from the main trial being available.

Patient and Public Involvement Statement

PPI input was not used directly with this process evaluation although one lay advisor, recruited via UNTRAP (Universities/User Teaching and Research Action Partnership) who helped with the development of I-WOTCH and its intervention, stayed on to become a valuable team member. Study findings will be disseminated via newsletters and a lay summary put onto the study website as well as feedback to UNTRAP and other partnerships as outlined in the I-WOTCH protocol paper[5].

For peer review only - http://bmjopen.bmj.com/site/about/guidelines.xhtml

Findings

1) Experiences of the I-WOTCH interventions

We had multiple sources of qualitative data (Figure 1).

Figure 1 – Qualitative data sources

Participant interviews

The 40 interviewees (20 Control/20 intervention) had a mean age of 65, range 59-72 years, 38/40 (95%) were white and 25 (63%) were female. Half (50%) had been on opioids for >5 years and 31 (78%) had pain for >5 years. See supplementary material 7 and 8 for full interviewee characteristics and compared to the main study demographics. Eighteen participants declined an interview mostly because of 'health issues' (e.g. appointments or operations) or 'not a good time' (e.g. work commitments, too busy).

Each interviewee has been given a numerical identifier, which follows each presented quotation. Table 2 provides evidence from the control interviews.

Control Interviews

The majority found the self-help booklet useful for information and further resources they could access.

Table 2 Control exemplar quotes

Aspects of Control Intervention	Exemplar quotes
Some spoke about the information being a potential trigger to start the tapering process and that 'it made you think':<br>Some found the information about risks and possible side effects interesting or shocking.	*'…to make you think about what you're taking and why you're taking it'.08.*<br>*'…I was never told about the constipation problems that it would cause and obviously you read about the side-effects in the little leaflets and then it's put away!' 20*<br>*'sleep apnoea… …that was a bit scary..' 23*
A few interviewees used the self-help booklet to start tapering.	*'I just followed everything it was doing…I just came down off the pills…I've done more exercise…' 15*
A few didn't find it as useful:	*''…in a way it's assuming that you're overweight and you don't exercise!......it didn't tell me what I could do if I wasn't overweight…and I'm getting loads of exercise…… It doesn't really tell you what you can do as an alternative…28*
Some reported using the relaxation CD with good effect and found it helpful either regularly or when needed. Half had tried it once or twice but hadn't continued as it wasn't useful for them, but a few who used it more than a small number of times said that it wasn't useful overall.	

For peer review only - http://bmjopen.bmj.com/site/about/guidelines.xhtml

Enablers and barriers to tapering

In the interview schedule we asked all interviewees about enablers and barriers to tapering. As we are focussing on the I-WOTCH intervention for this paper, we illustrate the themes with quotations from the intervention arm only for clarity.

We analysed the data from all 40 patient interviews using framework analysis for enablers and barriers to the tapering process including any attribution to the I-WOTCH study from the participants' perspectives.

Enablers

We identified four themes; (i) Readiness to start tapering, (ii) I-WOTCH as a trigger or motivator, (iii) Continuing to taper (once they had started), and (iv) Living without opioids. Subthemes are listed in Table 3 with exemplar quotes from the intervention participants.

For peer review only

Table 3abcd Enablers to Tapering Opioids

**3a Theme 1: Readiness to Taper**	
**Theme 1 sub themes**	**Exemplar quotes (with participant number) – intervention**
Already made the decision or had started to taper	*and then the invitation to take part in the study so I was reasonably invested in actually engaging with the study because it was something that I'd been thinking of myself… if you understand… 29*
Didn't like the side effects	*so it was a case of well I would try and see if I could reduce them or come off them and see if I wouldn't need as much other medication so it's the side-effect medication so I wouldn't be taking the anti-nausea tablets I wouldn't be having the… mmm… bowel medication…34*
Not wanting to be on opioids	*… it was when it was on the tele as well about being an opioid I thought I don't want to be addicted or to be totally dependent on them…13*
Wondering if opioids were working anyway	*'Do you really need to be on this now?' ……so I was reasonably invested in actually engaging with the study because it was something that I'd been thinking of myself 29*
Support from GP or family to taper	*…I myself thought right I don't want to be on these anymore and the doctor agreed…25*
Positive past experience of tapering	*16 – 17 years ago just after I'd had my first pair of hip replacements before they started giving me problems I came straight off all the opioids I was taking then so I knew that I didn't really have a problem with… withdrawal from a previous experience…22*
The right time to fit with life events	*I'm trying to think back to that part of the summer… how I was like… think I was relatively well which is why I started reducing it…34*

**3b Theme 2: I-WOTCH as a trigger or motivator to taper**	
**Theme 2 sub themes**	**Exemplar quotes - intervention**
I-WOTCH as trigger	*… but being part of the study has… mmm… enabled me not to be on them anymore whereas I don't know whether that would've been the case otherwise!29*
Information	*… and then when I was on the course I realised how damaging these things could be and I got all this information off the ladies and went through that …. well there's only one thing you've got to do… you've got to come off these damn things…30*
Support from group or nurse	*…I think because I had the support …. I just felt encouraged like that I could do it meself so I liked the support I suppose and the fact that they believed in me…13*
Tools to help	*I was over the moon because I got more tips you know… … it was all helpful to me. 10*
Tapering plan - given the means	*… it was a joint plan that we worked out together… 09*
Open Mindset 'give it a go'	*… but I went in with an open mind and everything we did on the group the… the practice sessions and the things we got to do at home you know the colouring and everything else that we did on the study… mmm… and listening to the relaxation CD's and everything like that… I'm… I went in with an open mind on that and listened to it and took out what… what I could…..09*

**3c Theme 3: Continuing to taper**	
**Theme 3 sub themes**	**Exemplar quotes - intervention**
Self-efficacy	*… the Tramadol thing is still there I look at it at times and I sort of say 'maybe it's better that it's there instead of me throwing it away because that's training me' it's giving me enough courage to sort of say 'I'm straight I can actually see it and overcome it' and… err… I'm almost like it's like a mini training moulding myself on how to… to… to manage that…38*
Feeling better, decreased side effects	*but it was really important to me to get rid of this… rid myself really of this drug… all I can say is this complete… having from the other side now I describe as 'un-reality' 29*
Pain similar	*… but really I can say coming off of it the pain's not any worse than it was…31*
Seeing the reduction of opioids	*…so it just shows you… you just… you know if I can do without half of them I'm going to try and get down a bit further…21*

For peer review only - http://bmjopen.bmj.com/site/about/guidelines.xhtml

Flexibility (slow) of the tapering plan	*it was done so… so slowly and minutely it was… I… I can't honestly say I had any side-effects!31*
Participant in control	*I thought that was helpful you know to set your goals and know what you'd set 31*
Understanding/ weathering withdrawal effects	*I was kicking I was twitching I was… I couldn't sit still I was… Aah… me hands were going and… I'm not joking I had to run round… I was running the table or walking fast round the table 'cause I couldn't keep still… I couldn't get any comfort whatsoever or any satisfaction and I was like 'Jeeze' and a couple of times I went… thought shall I put a patch on but I no I've got to persevere because if I put a patch on I know it will stop but I am back to square one so no I've got to persevere… I've got to persevere, and I did and I carried on and that's it… eventually it dispersed and… great so I've never touched the patches since… it's really great I'm chuffed…05*
Support from group or nurse	*…and that's what was good about the course with the support and the support network …… but it's that conversation amongst yourselves because you all know you're in the same boat you know what I mean and a lot of ya will have… would've had similar experiences and similar thoughts and whatever it be… err… it's… it's that which I think motivates ya…22*

**3d Theme 4: Living without opioids**	
Living without opioids	*……because me body was getting itself put right if you understand where I'm at… …and in my mind I know I'm not abusing my body any more than what just for Paracetamols and everything I'm on now… 10*<br>*…there's negative points of coming off them but I feel the plus side is that these tablets… these opioids are not doing any harm to me body anymore…31*<br>*… because of the course you know the few weeks that we were on that was a great help to me it just urged me to get off you know… wean myself off them which I did! 25*

Barriers to tapering

Three barrier themes are from different phases of tapering; pre-tapering, during tapering and after stopping opioids. Subthemes are given in table 4abc below with exemplar quotes from the intervention.

Table 4abc Barriers to tapering

**4a Theme 1: Pre tapering**	
**Sub-themes**	**Intervention- exemplar quotes**
No intention: not receptive/ 'I'm not addicted'/ Not motivated	*… I think I'd be in a bad place if I couldn't take them… I certainly don't think I'm addicted to opioids 16*
No choice, no alternatives	*… I really don't want to be on them that's my wish but… mmm… it's taught me that I cannot be without them which is unfortunate….25*

For peer review only - http://bmjopen.bmj.com/site/about/guidelines.xhtml

Not the right time: Other health priorities / Won't fit in with life (holidays) / Awaiting procedures which may help or affect their pain e.g. knee operation/operative pain	*…… I'd not even considered that at all until… until I have the… my hip replaced and then that knee's done that hip's done me eyes are done then we can see… start tapering off maybe having one… one a day instead of two a day. 16*
Fear of: Increased pain / Decreased function / Not coping / Opioids being stopped / Having no future access to opioids	*'I remember thinking 'I'll be in so much pain and I'll be… I'll be even more angry with myself' so I was thinking, ' I really… I can't… I can't do this…21*
Resistance to group intervention messages: Anger at GPs- (Affecting GP/Patient relationship) / Scepticism	*… but I'm quite annoyed that a GP who's prescribing more and more and more and should ought to… jolly well ought to know the research background so… and there was a lot of anger in the room that I was in about that…29*<br>*but I was a little pessimistic… mmm… because of knowing how much pain I've been in… in the past and nothing else has worked…07*

**4b Theme 2: During Tapering**	
**Sub-themes**	**Intervention- exemplar quotes**
Increased pain	*I think I was wrong to come off them believe it or not… and I really want to get off them because that make one very constipated and I just wanted to be free of them but I couldn't manage it… Saturday and Sunday were dreadful days I was in such pain! 25*
Unpleasant withdrawal symptoms	*… but I'd say the symptoms were far worse when I reduced the second half …… … cramps in my legs and the… oh the hot sweats and cold sweats they went on for a long time that was horrible…01*
Tapering too fast	*… it's a shame that there wasn't another way or to have changed the patches in some way to have come down at a slower rate and perhaps then I may have had more success I think with coming down 25% because this particular patch that I'm on… the Butec one……it only comes in 5's…07*
GP unsupportive of the process or poor access	*…cos the doctor actually… err… didn't seem at all conversant with the idea…32*
Relatives worried not supportive of process	*… even my brother noticed he said 'you know it's been really rough for you coming off …07*
Missing trial support	*I know that I couldn't have done it without going into a group to begin with to have that information if someone had just sat me down and said ' you're doing to reduce this by this and this' I would be screaming I think… no, no I can't you know yeah.21*

**4c Theme 3: After stopping opioids**	
**Sub-theme**	**Intervention- exemplar quotes**
New or returned pain	*… I came completely off the opioids while I was doing the course… completely off them… erm… stopped them all… err… sadly I fell over in December… it caused me a load of problems I slipped a disc and whatever and hurt me left hip… err… so I went back on them to deal with the initial pain and if I'm completely honest with myself the pain's now bearable again but I've gone to that what I call my traditional crutch that I rely on…22*

For peer review only - http://bmjopen.bmj.com/site/about/guidelines.xhtml

The role of the general practitioner (GP)

Most interviewees spoke about their GP in relation to their opioid use and tapering and the role of GP is presented in Table 5. In the I-WOTCH study, intervention participants were given a tapering plan which was also sent to their GP and people were encouraged to make an appointment and discuss the plan. It was the GP who then actioned the plan if acceptable to both parties. The self-help booklet also suggested control participants could discuss tapering with their GPs. We have thus utilised quotations from intervention and control groups for this section on the role of the GP. There were five themes: (i) GP as information giver and sounding board, (ii) Trust in doctor, (iii) Teamwork, (iv) GP reluctance and (v) Access to GP. The table below gives exemplar quotes.

Table 5 Role of the GP

**Themes**	**Explanation**	***Exemplar quotes***
GP as information giver and sounding board.	Prior discussion with GPs helped participants to prepare. Often this meant more interest or confidence in IWOTCH.	*' Talking through [with the GP] made me realise I was ready' 4 control*
Trust in doctor	Participants spoke of a history of trying everything else before opioids and often upward titration. They accepted this as they trusted their doctor.	*I sort of trusted my doctor that he was doing the right thing by me… 28 control*
Teamwork	Some participants described a good working relationship with their doctor who supported and encouraged tapering.	*… I'd come down an awful lot and my doctor was absolutely gobsmacked… she calls me 'her star patient…' …she's a fantastic GP….. she wanted me to succeed as much as I did so it was teamwork but if you've got a good GP like that you're sailing aren't you but some are too busy! 31 intervention*
GP reluctance	There were some participants whose doctor was resistant to them tapering.	*'they didn't think I would get off them'[opioids] (because of their pain) 13 intervention*<br>*[You]Can't come off them we've tried all sorts 15 control*
Access to GP	Some participants felt it was difficult to get to see their GP	*… getting help really it's very difficult and getting an appointment up here is very difficult! 20 control*

For peer review only - http://bmjopen.bmj.com/site/about/guidelines.xhtml

Intervention Interviews – participants' experiences of the intervention (n=20)

We present 4 main themes with subthemes where appropriate.

1. *Acceptability of the intervention*
2. *Being in a Group*
   - *Shared experience*
   - *Social comparisons*
   - *Support*
   - *Being committed to something*
   - *Group numbers*
   - *Perceptions about group facilitators*
   - *Challenges to establishing group cohesion.*
3. *Group sessions of interest*
   - *Opioid Information*
   - *Distraction techniques*
   - *Anger irritability and frustration*
   - *Relaxation*
   - *Mindfulness*
4. *Nurse one to one consultations: participant perspectives*

Acceptability of the intervention

Half said that the intervention was delivered in an appropriate format. When asked about suggestions to improve it, key responses were; provision of more support especially as tapering progressed, more group time, less didactic delivery and more local venues. All agreed they would recommend the I-WOTCH approach to others although some noted that participants needed to be open and receptive.

Being in a Group

Looking back on the group intervention, most interviewees found that being in a group was beneficial. They spoke about the groups as a shared experience where they were with people in similar circumstances which included the lay facilitator with whom they could share experiences. There were social comparisons within the group with participants noticing that others were like, or not like, themselves. Social support from the group was a key theme with evidence of people encouraging others especially when they were experiencing difficulties. Interviewees often talked

For peer review only - http://bmjopen.bmj.com/site/about/guidelines.xhtml

about the commitment to the group and or the trial. Group numbers seemed important, less than three members limited discussion. Lay group facilitators were seen either as good examples of how people cope with chronic pain or, alternatively, that they were somehow not the same and may not have had the same long-term experience of opioids and pain. The former perception allowing greater identification with the lay facilitator. Not all groups ran smoothly especially if they included disruptive individuals (Figure 2).

Figure 2 Being in a group

For peer review only

Group session aspects of interest

There were five sessions which people talked about repeatedly across the data (see below) but on the whole people tended to talk globally about the intervention even when prompted to discuss individual sessions.

Box 2 Specific aspects spoken about repeatedly.

Aspects of Interest

Opioid Information:

Information sessions gave most participants new insights into opioids and their effects. Participants also learned about differences between dependence, tolerance and addiction and the effects of withdrawal and/or tapering and valued exploring these in the groups.

Distraction techniques:

Distraction techniques were felt to be the most useful technique, some recognised the technique in existing pastimes and hobbies.

Anger irritability and frustration:

This session was appreciated because participants felt they were not alone in having these feelings and that they had an opportunity to talk about them.

Relaxation:

Most liked the relaxation sessions but few used relaxation techniques regularly, as needed. Participants often confused relaxation with mindfulness.

Mindfulness:

Unless exposed to this previously, this was not well understood and often thought to be another form of relaxation. A few participants remained unsure about what it was.

For peer review only - http://bmjopen.bmj.com/site/about/guidelines.xhtml

Nurse one-to-one consultations: participant perspective

Most intervention participants talked about these sessions. A majority appreciated the tailoring of the tapering plan and how they could personally apply the elements they had learned from the intervention. Sessions were described as encouraging and supportive and almost all talked about having a good relationship with the nurse.

… she was hopeful she said I had the determination to succeed in coming off the Tramadol… erm… so I felt positive with that… that I had like sort of the support with the words that I could actually do it … … I just felt encouraged like that I could do it meself so I liked the support I suppose and the fact that they believed in me…yeah! 13

Participant feedback forms

Participants were given feedback forms at the end of day three in a sub-set of ten intervention groups, providing 31 responses (delays in submitting an amendment about this for ethical approval meant the earlier groups were not able to be asked to complete this feedback form). Overall, responses indicate positive appreciation of most aspects of the course, but this is a small sub-sample, reported in detail in supplementary file 9 and should be treated with caution.

Intervention delivery staff interviews

Staff Characteristics

We interviewed 18 intervention-delivery staff; nurse facilitators n=10, lay facilitators n=6 and other health care professional (HCP) facilitators n=2. Four (one nurse, 3 lay) did not respond. Identifier prefixes after each quotation are N for nurses, L for lay and H for healthcare professionals.

We have presented the four themes from the staff interviews: 1) Acceptability and training, 2) Running the group sessions (Venues, Facilitators and Group dynamics), 3) Factors affecting readiness to change across group sessions, and 4) One-to-one sessions.

For peer review only - http://bmjopen.bmj.com/site/about/guidelines.xhtml

Acceptability and training

Almost all were happy with the package as delivered. Quality assurance assessment on Day One was appreciated as was the general support from the team *"I wanted that there was somebody there on the first day actually because it was quite good to find out how… whether I was actually doing it [correctly]…"L05*

Staff interviewees regarded the training as adequate and the manual as comprehensive. Some would have liked more training on; practical facilitation and/or subjects with which they were not familiar e.g. mindfulness or teaching posture. Most put in extra unpaid time to read around relevant subjects and to practice their delivery, and most were happy doing this.

Running the sessions

- Venues varied greatly, particularly in community settings. There were instances when even working to a lone worker risk-assessment protocol, facilitators were asked to lock up, being last in the building. On a few occasions, telephone back up with the I-WOTCH team was compromised by a lack of signal. IT problems were mitigated by facilitators having written scripts as backup.
- Facilitators mostly worked well together, with only occasional disagreements over differences in delivery or choice of sessions. Most would have preferred to work in the same pairings which was not possible within the intervention delivery design. Nurses felt the days were quite 'full on' having little downtime because even in breaks and meal-breaks everybody still chatted. Lay facilitators who were having to deal with their own health issues commented that the travel and full days (09.00 to 15.00) left them tired. However, all the facilitators said that the lay facilitator role was key to the groups running well. "*… do not remove the lay facilitators if you're going forward, they have to be there…" N02*
- Group dynamics

For peer review only - http://bmjopen.bmj.com/site/about/guidelines.xhtml

There was sometimes initial scepticism and negativity about the programme which was challenging for the staff to deal with. Resistance sometimes changed around day 2.

"… I think by sort of early afternoon is when people started you know listening a bit more thinking, 'Oh ok maybe there is an alternative?'" L07

Groups fared differently – occasionally groups bonded or 'gelled' as early as day one but more often by the end of day two and definitely by day three where shared experiences and supportive bonds had been formed.

"Now dealing with positive people looking and moving forward." L04

The most challenging groups involved a small minority of participants actively resistant to the information and techniques offered. Some showed challenging behaviour such as 'rubbishing' the content or interrupting. Some of these participants did not return after day 1.

'Shutters down' straight away. L05

Factors affecting readiness to change across group sessions – Categories of change

Intervention delivery staff described three presentations of change experience (across participants) and two turning points around the two main opioid information sessions. See boxes 3 and 4 below.

Box 3 Three categories of change experience identified by intervention delivery staff

**Resistance to change**<br>Some did not want to engage, either that they were sceptical about the information, had no side effects or were fearful of coming off opioids. This was sometimes due to bad experiences or felt reliant on them or wanted something else as a substitute.<br>*… that was the bit they all dreaded… mmm… and they were super nervous and a lot of them I think weren't quite sure whether they wanted to take that step…L05*
**Open to Trying**<br>Some participants were ready to try – motivated themselves or by the group or that they felt the medication perhaps wasn't working anyway. There was concern after learning more about opioids especially its effects, tolerance and dependence. Tapering slowly was a reassuring message rather than 'all or nothing' enabling participants to 'just give it a go.'<br>*and sometimes they would talk themselves into it and talk themselves out of it… it was… cos it's a step into the unknown and we tell them this is what could possibly affect you …it did make it worse I… … cos some*

For peer review only

of the patients felt that they were already dealing with quite a bit you know to start with…… what made it really good was…… the other facilitator the nurse said you know 'it need not be a dramatic drop it can be a gentle tapering thing' and I think when they saw the tapering that settled them down at the end… L05
**Not the Right Time**<br>Staff spoke about participants wanting to delay tapering until after an event: e.g. Holiday or surgery. They felt some were not able to manage it at that time but could reduce a little and try again later.<br>*… there was only the one man at first on the first class who hadn't been on opioids long… didn't feel dependent on them yet but he said he'd learnt and he knew what to look out for so yes he would be you know aiming to come off them but not quite yet…* L04

Box 4 *Turning points identified by intervention delivery staff*

1) on the morning of day one some were shocked and unaware of the issues and surprised to learn opioids aren't helpful long term.<br>*Bit of an eye opener N09*<br>*I think they were surprised to learn that opioids aren't helpful long term… … they would've been I think of the view that… err… I need to take this because if I don't take it I'll be in even more pain rather than this doesn't seem to be working cos I'm still in pain after 20 years! N11*<br>*[Angry with Doctors] and I think they were quite cross that they were getting it and being giving it and the length of time they'd been given… mmm that didn't go down too well in any of the groups! L05*
2) on the morning of day two when they started to think how they may start tapering and asked more questions. This was after the second session about opioids which covered withdrawal and John's tapering story which worked well.<br>*…my opinion was that they really needed their hands holding if they were going to make the jump… H2*<br>*… and then on day two pretty much you can tell who is more open to it ……most of the time it was the majority of the group you'd have the one or two that were still sceptical during day two still kind of like asking us questions… L03*

One-to-one sessions: nurse facilitator perspective

Nurse facilitators spoke of the tapering app being straightforward to use, although at times they were unable to get a mobile phone signal and occasionally the opioid the patient was taking was not included in the tapering app. Hard copies of the tapering plan were also written. On discussing the tapering plans suggested on the app algorithm, nurses and participants often wanted to taper more slowly e.g., tramadol tablets decreasing by 50mgs but with no smaller dosages available, lack of confidence or having withdrawal effects. This was supported and nurses felt that it was important that the participant felt in control of the process.

For peer review only - http://bmjopen.bmj.com/site/about/guidelines.xhtml

..a lot of the participants … mmm.. didn't feel that they could taper at that speed and my sort of take on that is that it is a marathon not a sprint and you know if you can't taper at that speed then you know we'll go a bit further …' N10

Nurses described participants wanting to talk about a lot of issues which they hadn't necessarily brought to the group and that they needed that opportunity to talk.

'it gives them an opportunity to talk openly with me and perhaps mention things that they haven't been able to mention within the group.' N10

Some nurses spoke about seeing a difference in participants at their 1st and 2nd face to face.

..people used phrases like they'd felt they'd 'come from behind a curtain.' And they didn't seem quite so dazed…'N06

Nurse data suggested that confidence, motivation, and the participant being in control were key factors to consider in the tapering process.

3) Implementation of the I-WOTCH group intervention

This section explores how the trial was implemented and the uptake of the components offered (figure 3).

Figure 3 Structure of the implementation items

Groups run (dose delivered)

The trial ran 35 groups - 20 in the midlands, 15 in the northeast, of England. At randomisation the mean group size was 9 (IQR 5 to 11).

For peer review only - http://bmjopen.bmj.com/site/about/guidelines.xhtml

Groups delivered (dose received)

Uptake and attendance (dose received)

Minimal compliance is defined as attending at least Day 1 and the first one face to face consultation. Full compliance is defined as attending at least Day 1, 2 & 3, the first one to one consultation and at least one phone call.

We randomised 305 people to the group intervention with 166 (54%) attending all three group days. The first one to one session attendance was 190 (62%) and 131 (43%) attended both the first and second session. 167 (55%) received one or more telephone calls. Thirteen (4%) did not return after day one (as mentioned in the staff interviews). Non-attendance at the first and subsequent sessions was high at 90 (30%) (Full trial attendance see supplementary material 10).

Participant reasons for non-attendance throughout the study

Reasons for non-attendance (if given) were poor health, competing work commitments and family interests.

Fidelity

Fidelity score averages for the group sessions adherence was 83% - (very good 81-90% as rated by Borrelli et al 2011[14]), with a range of 25-100 and a median of 88 and overall competence 79% (good 71-80% [12]), range 0-100, median 86 [14]. One to one session scores were high with an adherence average percentage score of 91%, range 61-100 and competence an average of 93%, range 50-100 (excellent 91-100% [14]) (supplementary material 11 and 12).

For peer review only - http://bmjopen.bmj.com/site/about/guidelines.xhtml

4) Potential Change Mechanisms

The control and intervention scores are comparable over the items of each question at baseline. The two baseline only questions (2 and 3) (supplementary material 6) were; Baseline expectation: *I expect that, in 4 months' time, I will have reduced my opioid use* and Baseline self-efficacy *I am confident I could reduce my opioid use a lot over 4 months which* showed a trend of low expectations of successful tapering within 4 months and low self-efficacy/confidence in tapering (supplementary material 13).

The motivation to reduce opioids question showed a trend for more participants wanting to cut down or stop their opioids at baseline. There is a weak trend showing the self-management group to be more slightly more motivated to continue tapering at later timepoints. However, this should be viewed with caution due to the high proportion of missing data for this item.

Our statistician ran statistical analyses on the full data collected; baseline four, eight and 12 months to look for any impact of responses to the change mechanism questions on main trial outcomes (see supplementary material 14). There were few significant correlations except for the study perceived credibility question. Correlations indicate that more participants attributed their tapering success to involvement in the intervention arm of I-WOTCH with the reverse trend in the control group. (supplementary material 15).

For peer review only - http://bmjopen.bmj.com/site/about/guidelines.xhtml

5) Contextual issues

The main contextual issues were trying to live a life, often with multiple health problems and medications alongside managing multiple appointments, tests and operations. Day to day life is often a challenge dealing with their pain and the things they need to get done. Living with chronic pain can be challenging physically, mentally and emotionally. For some just the idea of starting to taper is ‘a step too far’ that they may be unwilling or unable to consider. This was seen in the participant interviews and the staff interviews especially with the three categories of change given previously in table 9 when people are; ‘Resistant to change’, ‘Open to Trying’ or that it’s ‘Not the Right Time.’

Overarching Themes from integration of qualitative and quantitative data

Analyses revealed threads across the qualitative and quantitative data which generated four main overarching themes: (1) the right time, (2) the backdrop of life with chronic pain, (3) needing support and (4) the group effect.

1. The right time. This theme encompasses evidence indicating whether someone is ready to change, including enablers or barriers to tapering and whether participants felt fully informed, motivated, and confident to be able to taper.
2. Life with chronic pain is often complex with many having multiple health problems and medications. This means that participants were living life around their pain, pain relief, healthcare appointments and procedures as well as social and family commitments. Each of these could affect their decision about when or whether to taper.
3. Support is needed at all phases of tapering. This could come from their family, GP or from a tapering intervention such as I-WOTCH which agreed a tailored tapering programme. If

For peer review only - http://bmjopen.bmj.com/site/about/guidelines.xhtml

family members or their GPs were ambivalent or against tapering, people were less keen to try it.

4. The group effect allowed for people taking opioid medication to come together to gain more information and skills in pain management as well as choosing whether to start on a tapering path as a joint group goal. They were given the opportunity to discuss and explore their fears and motivations about decreasing their medication.

The interaction between the overarching themes can be seen in our model. See figure 4. Participants make decisions about tapering based upon whether it is the right time for them, their current pain experiences and healthcare and the support available to them. Helping participants to identify and discuss potential barriers whilst fostering knowledge, motivation, confidence and self-management skills means that tapering is more likely to take place.

Figure 4 Conceptual model of overarching themes

For peer review only

Discussion

The I-WOTCH main trial results showed that the intervention was effective at helping participants to stop taking opioids or decrease their use by 50% without any difference in pain, or pain related disability, between the study arms. The process evaluation data indicates that the intervention was delivered as intended with good fidelity scores. There was evidence of participants attributing their opioid reduction to the I-WOTCH study. Participants and staff alike felt the package as whole was acceptable and deliverable. However poor attendance was an issue with 90 participants randomised to the intervention not attending any group.

The secret ingredient?

No one factor or 'secret ingredient' was identified but rather a combination of active ingredients as enablers of opioid tapering. The I-WOTCH intervention seemed to have a synergistic, summative effect from its different components which could be specific to the individual. The qualitative data

indicate that the intervention served to inform and provide a forum where discussion in groups and in one-to-one sessions could help people decide whether to reduce their opioid use. The final decision was the participants, and no judgements were made about whether or when they might start tapering. The I-WOTCH intervention provided a space in which people could fill gaps in their knowledge of opioids. The intervention also enhanced motivations to taper, including intrinsic motivations that participants brought to the sessions. Information provided by the intervention about the lack of long-term effectiveness and side effects developed motivation. This was emphasised in staff interviews which highlighted how provision of information about opioid use consequences was, for some participants, a turning point in becoming motivated to change. Support from their GP, their family, the I-WOTCH one-to-one meetings, the tapering plan and group peers also enhanced motivation. Exploring pain management skills in a group over three sessions allowed them to try things out and swap ideas, so establishing the skill base needed for change.

The Information, Motivation and Behavioural Skills model[8,9] provided the theoretical foundation and guided our understanding of key components of change that would support action towards opioid tapering. The qualitative data confirm the utility of identifying sources of information, developing motivation at the right time and skill development, including use of the lay facilitator as a role model, as critical precursors of change. This process evaluation reveals how the I-WOTCH intervention provided all three of these critical supports. Barriers to change may be specific to the individual but supporting people to become informed and to develop motivation and skills optimises the chances of behaviour change. The main barriers were that it might not have been the right time for participants to consider tapering and other things in life often took priority. Fears of returning to uncontrolled pain were common alongside multiple other potential barriers.

These findings correspond to a wider literature. A 2020 qualitative evidence synthesis using meta-ethnography (31 studies) looking at experiences of people taking opioids for chronic non-malignant

For peer review only - http://bmjopen.bmj.com/site/about/guidelines.xhtml

pain by the current authors [15], found five themes, four of which resonated with our findings[16]. The fifth theme was around societal stigma, which did not emerge from the data in this English study but was common in North American studies.

Our findings are also broadly supported by recent literature. Goesling et al (2019) who held focus groups with former opioid users about their experiences before, during and after opioid cessation [15]. Motivators to stop included concerns about a lack of opioid efficacy, addiction, and quality of life impact. Quinlan et al (2021) noted that 60 patients undertaking a pre tapering survey found there were more barriers given than motivators [18]. The main areas of concern were around quality of life, pain and withdrawal which the authors suggest need to be addressed for successful tapering to take place. Henry et al (2019) conducted focus groups and interviews with 21 adults with chronic pain at different stages of opioid tapering [19]. They explored the complex contextual backdrop of life with chronic pain describing changes or fluctuations in many areas including pain, social relationships, health status, emotional state and the perceived need for opioids at any given time. They recognise the substantial effort involved for people undertaking the tapering journey, the effect on their day to day lives and the strategies used. They suggest early anticipatory guidance about tapering and that tapering should be patient centred and responsive to the patients' needs.

We identified GPs as integral to tapering support. Three 2020-22 papers from health care providers perspective[20-22] throw light on the complex challenges involved in tapering opioids, especially the importance of maintaining a good patient /provider relationship. Hamilton et al 2021 also cite patient motivation to be a central factor for successful tapering[22]. GPs are seen as authoritative experts in relation to health behaviour and patients often welcome behaviour change advice from their GPs, especially when such advice may have a positive effect on long-term condition management [23].

There is a long history of successful use of small groups to develop support, share common health problems, build trust, share ideas, enhance information, change attitudes, and develop motivation and

For peer review only - http://bmjopen.bmj.com/site/about/guidelines.xhtml

skills to promote health behaviour change [24, 25]. This evaluation shows that the I-WOTCH intervention successfully applied these group processes in supporting individual change. Participants often had a strong sense of shared experience around opioid use and the challenges of use reduction. They felt as if they were all in the same boat. This is a key foundation for successful group interventions [26]. There is also evidence of participants experiencing social support and social learning both key to individual change. Facilitation of interaction in small groups is critical to optimising change and interviews revealed that the lay facilitator role was very important to participants and nursing facilitators alike. Facilitators did not underestimate the challenges some participants might face and appreciated the lay facilitator's personal experience with which participants could identity and then learn from. The challenges of group facilitation observed also emphasise the importance of careful skill-based training for group facilitators [25].

Conclusion

Our conceptual model shows the active ingredients involved in an ideal pathway to successful tapering. This may look straightforward at first glance; however, the reality is that there are multiple contextual issues and barriers which influence someone's ability to taper. The I-WOTCH intervention gave practical strategies, information, support, room for discussion and a tailored tapering plan to help participants navigate this difficult journey.

Acknowledgements

We would like to thank the participants and intervention delivery staff who were interviewed for their valuable input into the process evaluation. Also the members of the I-WOTCH team.
Alleyne, Sharisse
Balasubramanian, Shyam
Betteley, Lauren
Booth, Katie
Carnes, Dawn
Furlan, Andrea D

For peer review only - http://bmjopen.bmj.com/site/about/guidelines.xhtml

Haywood, Kirstie
Iglesias Urrutia, Cynthia Paola
Lall, Ranjit
Manca, Andrea
Mistry, Dipesh
Noyes, Jennifer
Rahman, Anisur
Shaw, Jane
Tang, Nicole K Y
Taylor, Stephanie
Tysall, Colin
Underwood, Martin
Withers, Emma J

Collaborators

Author contribution:
Conceived the original study design: KS, MU, SE and HKS
Developed the study design and plan for data collection and undertook analysis. VPN, KS and CA
All authors have provided critical revisions to the manuscript and approved the final manuscript.

Funding

This project is funded by the National Institute for Health Research (NIHR), Heath Technology Assessment (HTA) (project number 14/224/04. The views and opinions expressed therein are those of the authors and do not necessarily reflect those of the HTA, NIHR, NHS or the Department of Health.

Competing interests

MU is chief investigator or co-investigator on multiple previous and current research grants from the UK National Institute for Health Research, and Arthritis Research UK and is a co-investigator on grants funded by the Australian NHMRC and Norwegian MRC. He was an NIHR Senior Investigator until March 2021. He is a director and shareholder of Clinvivo Ltd that provides electronic data collection for health services research. He receives some salary support from University Hospitals Coventry and Warwickshire He is a co-investigator on two current and one completed NIHR funded studies that are, of have had, additional support from Stryker Ltd. Until March 2020 he was an editor of the NIHR journal series, and a member of the NIHR Journal Editors Group, for which he received a fee.

KS is chief or co-investigator on many previous and current grants from the UK National Institute for Health Research. She also sat on a NIHR funding board (HS&DR) until 2018.

SE is a clinician and Chief investigator on several past and ongoing trials, his department has received research support from the NIHR as well as Medtronic, Boston Scientific and Saluda Medical. He is a paid consultant to Medtronic, Saluda Medical and Mainstay Medical.

Data Sharing: Data available upon reasonable request.

For peer review only - http://bmjopen.bmj.com/site/about/guidelines.xhtml

References

1. Moore GF, Audrey S, Barker M, et al. Process evaluation of complex interventions: Medical Research Council guidance. 2015;350.
2. Skivington K, Matthews L, Simpson SA, et al. A new framework for developing and evaluating complex interventions: update of Medical Research Council guidance. *BMJ*2021;374:n2061.
3. Nichols VP, Abraham C, Eldabe S, et al. Process evaluation protocol for the I-WOTCH study: an opioid tapering support programme for people with chronic non-malignant pain. *BMJ Open*2019;9:e028998.
4. Sandhu HK, Shaw J, Carnes D, et al. Development and testing of an opioid tapering self-management intervention for chronic pain: I-WOTCH. *BMJ Open*2022;12:e053725.
5. Sandhu HK, Abraham C, Alleyne S, et al. Testing a support programme for opioid reduction for people with chronic non-malignant pain: the I-WOTCH randomised controlled trial protocol. *BMJ Open*2019;9:e028937.
6. Sandhu HK, Booth K, Furlan AD, et al. A group-based educational intervention for reducing opioid use for chronic pain: a randomized clinical trial *JAMA* 2023.
7. Linnan L and Steckler A. Process evaluation for public health interventions and research San Francisco: Jossey-Bass, 2002.
8. Fisher JD, Fisher WA. Changing AIDS risk behavior. Psychological Bulletin. 1992;111:455–474.
9. Fisher JD, Fisher WA. & Harman, J. (2003). The Information Motivation–Behavioral Skills Model: A general social psychological. In, Social Psychological Foundations of Health and Illness (Eds. Suls, J & Wallston, K A), Blackwell Publishing Ltd. Oxford.
10. Ritchie J and Spencer L. In: The qualitative researcher's companion. In: Huberman AM, & Miles, M. B. (Eds.), (ed.). *Qualitative data analysis for applied policy research.*: SAGE Publications, Inc 2002: p. 305–29.
11. Braun V and Clarke VJQRP. Using thematic analysis in psychology. 2006;3.
12. O'Cathain A, Murphy E and Nicholl J. Three techniques for integrating data in mixed methods studies. *BMJ*2010;341:c4587.
13. GOV.UK. Data Protection Act. In: Collections, (ed.). 2018. https://www.legislation.gov.uk/ukpga/2018/12/contents/enacted. Accessed 4-4-23
14. Borrelli B, Sepinwall D, Ernst D, et al. A new tool to assess treatment fidelity and evaluation of treatment fidelity across 10 years of health behavior research. 2005;73.
15. Nichols VP, Toye F, Eldabe S, et al. Experiences of people taking opioid medication for chronic non-malignant pain: a qualitative evidence synthesis using meta-ethnography. *BMJ Open*2020;10:e032988.
16. Paulozzi L and Baldwin G. CDC grand rounds: prescription drug overdoses - a U.S. epidemic. *MMWR Morb Mortal Wkly Rep*2012;61:10-3.
17. Goesling J, DeJonckheere M, Pierce J, et al. Opioid cessation and chronic pain: perspectives of former opioid users. *Pain*2019;160:1131-45.
18. Quinlan J, Willson H and Grange K. Hopes and fears before opioid tapering: a quantitative and qualitative study of patients with chronic pain and long-term opioids. *British Journal of Pain*2021;15:120-28.
19. Henry SG, Paterniti DA, Feng B, et al. Patients' Experience With Opioid Tapering: A Conceptual Model With Recommendations for Clinicians. *J Pain*2019;20:181-91.

For peer review only

20. Langford AV, Gnjidic D, Lin CC, et al. "The lesser of two evils": a framework analysis of consumers' perspectives on opioid deprescribing and the development of opioid deprescribing guidelines. *Pain*2021;162:2686-92.
21. Matthias MS, Talib TL and Huffman MA. Managing Chronic Pain in an Opioid Crisis: What Is the Role of Shared Decision-Making? *Health Commun*2020;35:1239-47.
22. Hamilton M, Mathieson S, Gnjidic D, et al. Barriers, facilitators, and resources to opioid deprescribing in primary care: experiences of general practitioners in Australia. *Pain*2022;163:e518-e26.
23. Keyworth C, Epton T, Goldthorpe J, et al. Perceptions of receiving behaviour change interventions from GPs during routine consultations: A qualitative study. *PLoS One*2020;15:e0233399.
24. Borek AJ and Abraham C. How do small groups promote behaviour change? An integrative conceptual review of explanatory mechanisms. *Applied Psychology: Health and Well-Being*2018;10:30-61.
25. Borek AJ, Abraham C, Greaves CJ, et al. Identifying change processes in group-based health behaviour-change interventions: development of the mechanisms of action in group-based interventions (MAGI) framework. *Health Psychol Rev*2019:1-21.
26. Borek AJ, Abraham C, Greaves CJ, et al. 'We're all in the same boat': A qualitative study on how groups work in a diabetes prevention and management programme. *Br J Health Psychol*2019;24:787-805.

For peer review only

For peer review only - http://bmjopen.bmj.com/site/about/guidelines.xhtml

For peer review only

For peer review only - http://bmjopen.bmj.com/site/about/guidelines.xhtml

Figure 1 Qualitative data sources

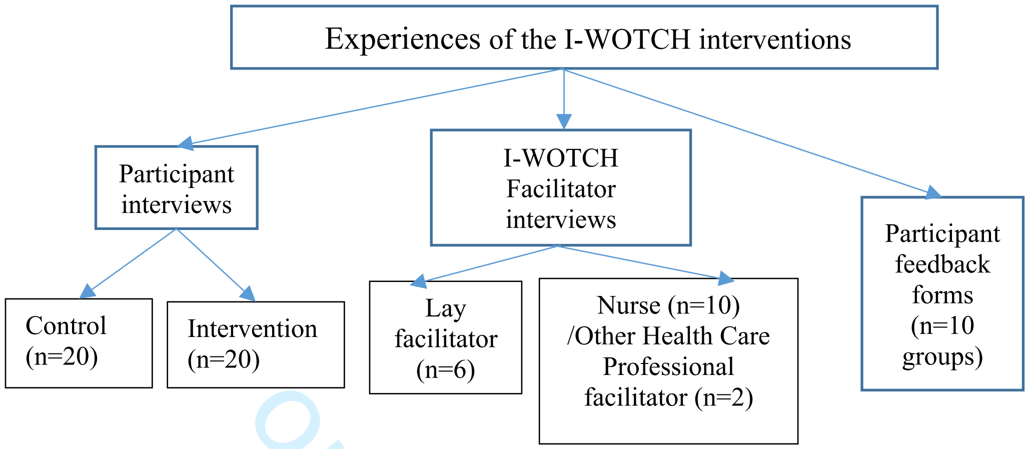

For peer review only

Figure 2 Being in a group

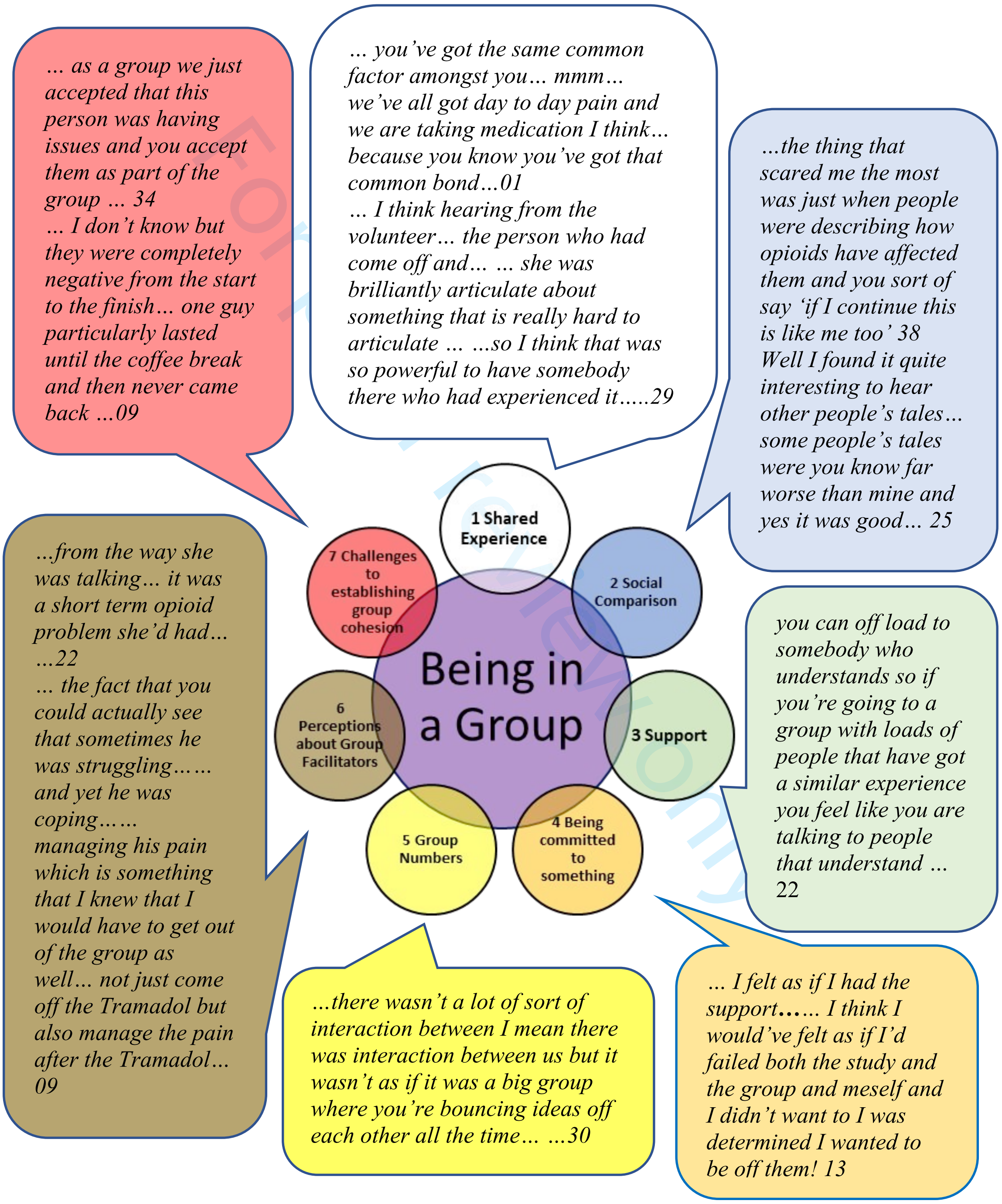

Figure 3 Structure of the implementation items

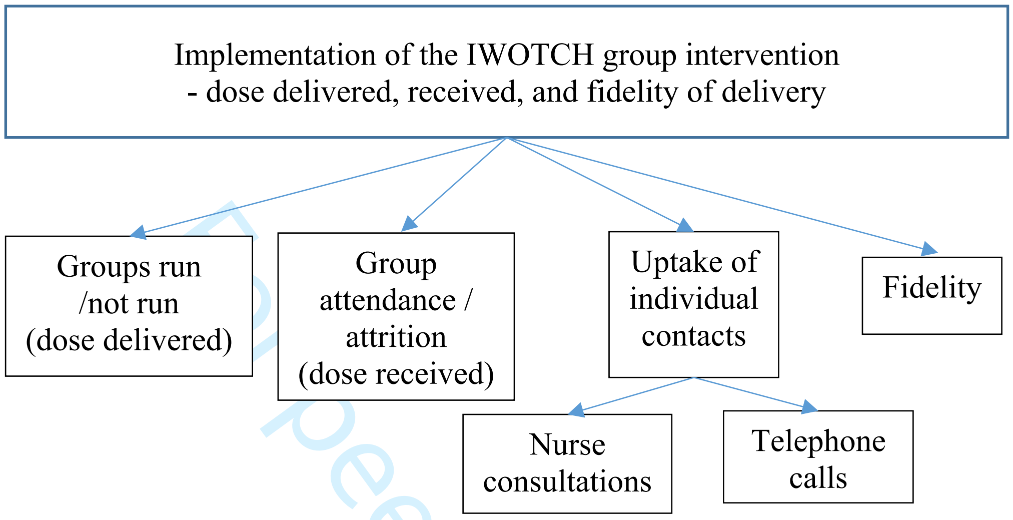

For peer review only

Figure 4 Conceptual model of overarching themes

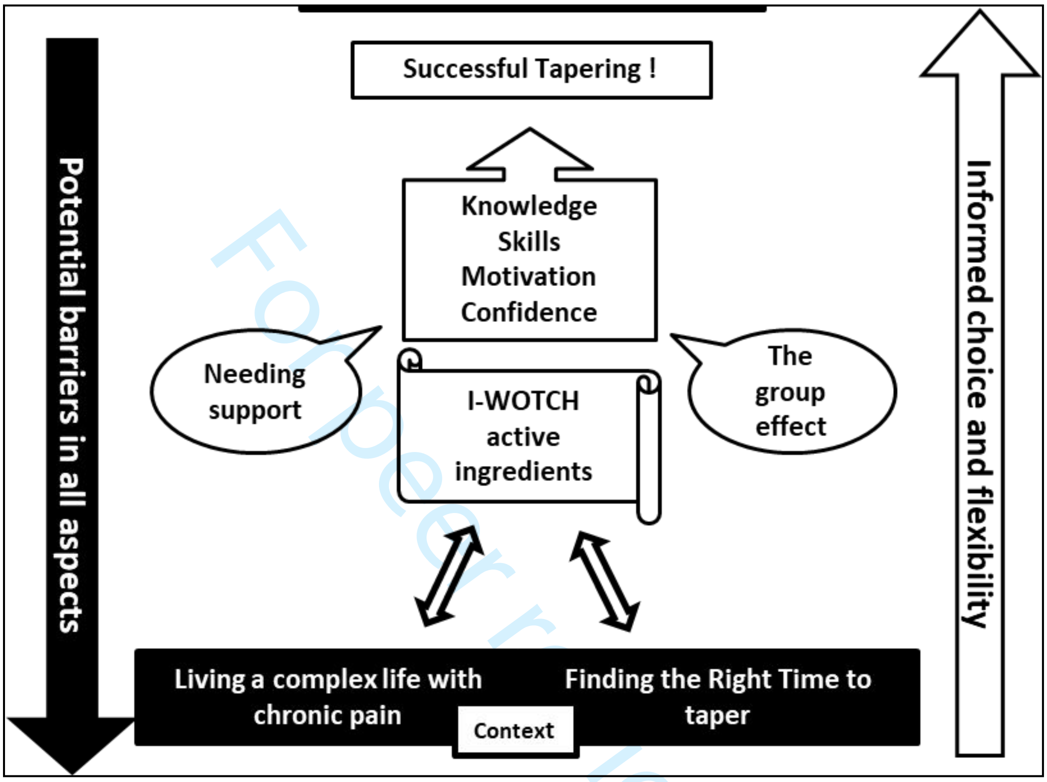

For peer review only

I-WOTCH Process evaluation supplementary material 1-10

Supplementary material 1 Logic Model

The problem	Intervention Aims	Intervention	Theory and Guidance	Interim Targets	Desired Outcomes
People with chronic non-malignant pain are taking opioids, which have side effects and are not effective in the long term.	To test the effectiveness and cost effectiveness of a patient-centred multicomponent self-management intervention targeting withdrawal of strong opioids on activities of daily living for people living with chronic non-malignant pain	**Manualised Intervention Delivery**<br>**Core pain management topics:**<br>• Acute versus Chronic pain<br>• Acceptance<br>• Attention Control and distraction<br>• the pain cycle<br>• Posture and movement advice<br>• Relaxation techniques<br>• Stress busting for health action planning, problem solving, pacing, SMART goal setting<br>• identifying and overcoming barriers to change<br>• Mindfulness<br>• Anger, irritability and frustration<br>• Communication Skills<br><br>**Core opioid specific topics:**<br>• The rationale of prescribing in chronic pain<br>• Opioid induced tolerance and need for dose escalation<br>• Evidence of usefulness of opioids short and long term<br>• Side effects of opioids short term and long term<br>• Case studies of successful discontinued opioid therapy<br>• Opioid withdrawal symptoms	Theory of Planned Behaviour<br><br>Social Cognitive Theory<br><br>Information Motivation and Behavioural (IMB model) skills<br><br>Patient Centred Communication<br><br>Motivational Interviewing	**Staff Training**<br>To facilitate groups, deliver individual tapering consultations and telephone support in an inclusive and non-judgemental manner<br>**Individual participant changes:**<br>a Knowledge of: opioids, withdrawal effects, chronic pain<br>b Fostering change: self-validation, legitimising pain, normalising expectations<br>c Motivation to change by: Improved self-efficacy, effective tapering<br>d Skills:<br>• General Self-Regulation<br>*Psychological skills*<br>Identify reasons for negative emotions (anger /frustration /irritable)<br>Identify problems and solutions, barriers to change<br>Recognise errors in thinking/automatic thoughts<br>Goal setting, goal review<br>*Physical skills*<br>Promote body awareness, posture<br>Reduce muscle tension<br>Body awareness and core strength<br>Relaxation-contract relax<br>• Pain Self-Regulation<br>Understand that pain and mood are linked – when is pain bearable and when not bearable.<br>Understanding of pain cycle, unhelpful emotions and behaviours<br>Using mind to relieve pain does not mean pain in mind<br>Distraction whilst relaxed	**Primary outcomes:**<br>Patient-Reported Outcomes Measurement Information System (PROMIS) Pain Interference Short Form (8A)(PROMIS -PI-SF-8A)<br><br>Daily morphine equivalent opioid dose

For peer review only

		• Advantages of slow supervised tapering<br>• Symptom management during tapering<br>• Pain control after opioids		Focus mind away from pain<br>Mindfulness for pain<br>Managing flare ups<br>Need for stretching<br>• <u>Communication Skills</u><br>How to communicate with General Practitioners (GPs) and Health Care Professionals (HCPs)<br>Listening skills - Active and giving feedback in communication-reward for help.	

For peer review only

Supplementary material 2 I-WOTCH Indicative Interview Topic Guide

Patient Participants

What did you hope to achieve by participating in this research?

Do you want to reduce your opioid intake- and if so why?

How important is it to you to reduce your opioid intake (that is, how strongly motivated are you)?

What were your expectations about whether this would work for you once you heard which group you would be in? (Intervention [I-WOTCH]) /best usual care [my opioid manager plus relaxation package])

How did your expectations match your actual experience?

How did you find using I-WOTCH/my opioid manager/relaxation package? (Where any parts easy for you? Were any parts difficult for you? Why was this?)

Did you find you were you able to use all the different components or not? (Prompt list of the components of intervention/control). If not, which components did you use? Why was this? Were there components you rarely or never used? Why was this?

What was it like trying to use this in your everyday life? (barriers/enablers)

Intervention group only: I'd like to ask you about your experiences of being in a group – how was that for you? (What was good about it, what was less good about it?)

Following from above.... How did the group work, was it better than having one-to-one sessions? Why?

How well was the group facilitated?

If you could change three things about the intervention what would those be?

If another patient asked you about taking part in this programme, what would you say?

Is there anything else you'd like to say that is important to you that I haven't asked you?

For peer review only

Supplementary material 3 Random sampling chart

	Group Number																													
	1	2	3	4	5	6	7	8	9	10	11	12	13	14	15	16	17	18	19	20	21	22	23	24	25	26	27	28	29	30
Sample 1		2		1		3					2			1			3				2		1	3						

For peer review only

Supplementary material 4 Fidelity data collection checklist (sessions in bold assessed for fidelity)

	*Educational and/or self-management regarding pain or opioid use	Practical, reflection or summarising sessions (not assessed for fidelity, recorded if took place or not)
Day 1	**2, 3, 4, 7, 8**	1,5,6,9,10,11
Day 2	**13, 14, 16**	12,15,17,18,19
Day 3	**21, 22, 23**	20,22 part 2, 24,25,26,27,28

**DAY 1**
Session 1 Introduction
***Session 2 Pain information**
***Session 3 Painkiller information and opioid education**
***Session 4 Acceptance: John's story**
Session 5 Attention Control and distraction
Session 6 Distraction activity – rose drawing
***Session 7 Good days, Bad days when is pain bearable and when is it not?**
***Session 8 The pain cycle unhelpful emotions and behaviours**
Session 9 Posture
Session 10 Relaxation and Breathing
Session 11 Summary of the day

**DAY 2**
Session 12 Reflections from day 1
***Session 13 Stress-busting – prioritising what's important, action planning, goal setting and pacing**
***Session 14 Withdrawal symptoms, case studies (Opioid Education 2)**
Session 15 Distraction activity- origami
***Session 16 Identifying and overcoming Barriers to change Part 1 – recognising unhelpful thinking**
***Identifying and overcoming Barriers to change Part 2– reframing negatives to positives**
Session 17 Mindful attention control
Session 18 Balance and introduction to stretch
Session 19 Summary of the Day

**DAY 3**
Session 20 Reflections from day 2 and previous week
***Session 21 Anger, irritability and frustration**
***Session 22 Relationships Part 1 Getting the most from your healthcare team**
Session 22 Part 2 Relationships Part 2 Listening skills
***Session 23 Managing setbacks and non-drug management techniques**
Session 24 Distraction activity – mindfulness colouring
Session 25 Stretching muscles that commonly get tight
Session 26 Mindfulness of thoughts and Senses
Session 27 Summary of Day 3
Session 28 Summary of the course

For peer review only

Supplementary material 5 Examples of I-WOTCH fidelity sessional and nurse consultation score sheets for adherence and competence

Day 1 /Session 2 /Title: Pain Information 30mins

Adherence: of the delivery as per protocol

Instructions: When at all possible please rate as 'Yes' or 'No' If 'partially' then write reason in comments box. Questions need not be verbatim (unless specified) as long as content of session is covered.

For peer review only

No.	Item	Adherence	Comments
Intro	Did the facilitator(s) introduce the session?	Yes (2) Partially (1) No (0)	
Step 1	Did the facilitator(s) play the DVD of the biomedical explanation about acute and chronic pain?	Yes (2) Partially (1) No (0)	
	Did the facilitator(s) ask the group Q1 and discuss, "What do you think about this explanation of pain? Is it missing anything?"	Yes (2) Partially (1) No (0)	
Step 2	Did the facilitator(s) present the bio-psycho-social explanation of pain?	Yes (2) Partially (1) No (0)	
	Did the facilitator(s) ask the group Q2 and discuss, "What do you think about this explanation of pain?"	Yes (2) Partially (1) No (0)	
Step 3	Did the facilitator(s) play the DVD of Experiences of living with opioid- treated long term pain?	Yes (2) Partially (1) No (0)	
	Did the facilitator(s) ask the group Q3 and discuss, "What do you think about Caroline's description of living with opioid-treated long-term pain?"	Yes (2) Partially (1) No (0)	
Summary	Did the facilitator(s) consolidate/embed the group's learning at the end of the session? *e.g. reading the summary, putting the session in context*	Yes (2) Partially (1) No (0)	
	Total adherence score (max 16)		
	Percentage adherence score (Total adherence score */16x100)		
**Comments:** For use if sessions; go off track, include items which are not on checklist, contain surprising unforeseen aspects or the facilitation wasn't covered as intended. Also if there was no opportunity to demonstrate the skill listed.			

Day 1 /Session 2 /Title: Pain Information and Opioid Education

<u>Competence:</u> of the quality of delivery or 'skill' of the facilitators

	Item	Competence measure	Comments (*use box below to expand)*
1	Did the facilitator(s) create opportunities for discussion *e.g. did they; encourage individuals to participate, ask open questions, give enough time for the group to answer (rather than answer their own questions)*	Evident (2)<br>Partially evident (1)<br>Not evident (0)<br>Did not happen in this session (N/A)	

For peer review only

2	Did the facilitator(s) encourage individual disclosure? *e.g. did they ask different group members to comment or encourage the group to explore issues further (either individually or as a group)?*	Evident (2)<br>Partially evident (1)<br>Not evident (0)<br>Did not happen in this session (N/A)	
3	Did the facilitator(s) validate participants' disclosures? *e.g. Do other people find this/think that? I know how you feel. Sometimes people may feel differently about things.*	Evident (2)<br>Partially evident (1)<br>Not evident (0)<br>Did not happen in this session (N/A)	
4	Did the facilitator(s) give encouraging feedback on participants reported behaviours? *e.g. Did they give appraisal 'that's really good' or 'that's really good but I wonder if…'*	Evident (2)<br>Partially evident (1)<br>Not evident (0)<br>Did not happen in this session (N/A)	
5	Did the facilitator(s) foster a positive group climate? *e.g. did they; use humour, say positive things about people 'that's a helpful comment' 'thank you for sharing that'*	Evident (2)<br>Partially evident (1)<br>Not evident (0)<br>Did not happen in this session (N/A)	
6	Did the facilitator acknowledge and respond appropriately to admissions or statements of low self-efficacy? e.g. *'yes this can be difficult but…' ideas or examples offered of how this may be done. Issues surrounding confidence.*	Evident (2)<br>Partially evident (1)<br>Not evident (0)<br>Did not happen in this session (N/A)	
7	Did the facilitator respond appropriately to disclosures of negative events or barriers to progress?	Evident (2)<br>Partially evident (1)<br>Not evident (0)<br>Did not happen in this session (N/A)	
	Total competence score (max 14)		
	Percentage competence score		
**Comments:** For use if sessions; go off track, include items which are not on checklist, contain surprising unforeseen aspects or the facilitation wasn't covered as intended. Also if there was no opportunity to demonstrate the skill listed.			

For peer review only

Fidelity score sheet for first one to one nurse consultation

Item	Adherence	Score	Comments *(expand in box below)*
1	Was the participant asked about their thoughts/feelings on reducing their opioids?	Yes (2)<br>Partially (1)<br>No (0)	
2	Was the participant asked about their pain related medication usage?	Yes (2)<br>Partially (1)<br>No (0)	
3	Was the participant's pain discussed?	Yes (2)<br>Partially (1)<br>No (0)	
4	Was a tapering plan discussed/negotiated?	Yes (2)<br>Partially (1)<br>No (0)<br>Not evident	
5	Were temporary withdrawal side effects mentioned?	Yes (2)<br>Partially (1)<br>No (0)	
6	Was a tapering plan summarised and a copy given to the participant?	Yes(2)<br>Partially (1)<br>No (0)	
7	Were barriers to implementing the tapering plan and setbacks discussed?	Yes (2)<br>Partially (1)<br>No (0)<br>Not evident	
8	Did the nurse leave an opportunity for any questions?	Yes (2)<br>Partially (1)<br>No (0)	
9	Did the nurse ensure the participant knew to make an appointment with the GP?	Yes (2)<br>Partially (1)<br>No (0)	
	Total score out of 14 or 18 max		
	Percentage score total score/ 14 or 18 x 100		
Comments:			

Item	Competence	Score	Comments *(expand in box below if nec.)*

For peer review only

1	Did the nurse allow participants to express their concerns/achievements?	Evident (2)<br>Partially evident (1)<br>Not evident (0)	
2	Did the nurse allow participants to discuss and/or explore their concerns/achievements?	Evident (2)<br>Partially evident (1)<br>Not evident (0)	
3	Did the nurse demonstrate empathy? e.g. *Did they show they understood the participant's feelings?*	Evident (2)<br>Partially evident (1)<br>Not evident (0)	
4	Did the nurse accept the participant's perspective? e.g. *Did they allow exploration of positive and negative feelings about pain and/or opioids, were they non-judgmental?*	Evident (2)<br>Partially evident (1)<br>Not evident (0)	
5	Did the nurse actively listen to the participant? e.g. *Did they use 'uhuu', 'oh' 'um' 'really' type of phrases to demonstrate they were listening*	Evident (2)<br>Partially evident (1)<br>Not evident (0)	
6	Did they support self-efficacy? *e.g. Did they offer reassurance, suggest other techniques, congratulate them on any successes or small steps in the right direction.*	Evident (2)<br>Partially evident (1)<br>Not evident (0)	
	Total score out of 12 max		
	Percentage score total score/ 12 x 100		
Comments:			

Supplementary material 6 Change Mechanism Questions

Motivation, expectation, self-efficacy and perceived intervention efficacy questions

Baseline motivation (baseline and follow-up).

I want to reduce my opioid use.

(Not at all, by a little, by half, so I only use a little, so I use no opioids)

Baseline expectation (baseline only).

I expect that, in 4 months' time, I will have reduced my opioid use.

(Not at all, by a little, by half, so I only use a little, so I use no opioids)

Baseline self-efficacy (baseline only).

I am confident I could reduce my opioid use a lot over 4 months.

(Not at all confident, somewhat confident, fairly confident, strongly confident, completely confident)

Perceived intervention efficacy (baseline and follow-up).

Baseline

I feel that involvement in this study can help me to reduce my opioid use.

(Not at all, by a little, by half, so I only use a little, so I use no opioids)

Follow-up

I feel that involvement in this study has helped me to reduce my opioid use.

(Not at all, by a little, by half, so I only use a little, so I use no opioids)

For peer review only

Supplementary material 7 Interviewee characteristics and uptake for the interview study

Interviewee ID	Location cohort ID NE (Northeast) orblank(midlands)	Gender	Age decade	Opioid usage at 12m compared to baseline*	Opioid Type	Baseline Morphine Equivalent (ME) **	Allocation***
Pilot 1	P1	F	70's	0	Di hydrocodeine / morphine	30-59	Int
3	1	F	30's	S	Tramadol MR	0-29	Intention to treat
4	1	F	50's	L by half	Tramadol	0-29	UC
5	2	M	60's	0	Buprenorphine patches	60-89	Int
6	3	M	60's	H	Tramadol and cocodamol	0-29	UC
7	3	F	50's	L sl	Buprenorphine patches	30-59	Int
8	5 NE	F	60's	0	Zomorph(morphine)	30-59	UC
9	5 NE	M	60's	0	Tramadol	0-29	Int
10	4 NE	F	50's	0	Fentanyl patches	30-59	Int
11	6	F	40's	S	Morphine SR and Oromorph	150+	UC
12	7 NE	F	50's	L	Oxycodone and liquid morphine	90-119	UC
13	12 NE	F	50's	0	Tramadol	0-29	Int
14	9 NE	M	60's	S	Tramadol	0-29	Int
15	11	F	70's	H *(?)*	Tramadol	0-29	UC
16	11	M	60's	H	Oxycodone	30-59	Int
17	10	M	70's	S	Tramadol	0-29	UC
18	8	F	80's	0	Morphine	0-29	UC
19	17 NE	M	60's	L sl.	Tapentadol /morphine/ liquid morphine	60-89	UC
20	9 NE	M	60's	L	Morphine / Codeine phosphate / Tramadol	60-89	UC
21	15	F	60's	L sl	Morphine / liquid morphine	150+	Int
22	8	M	50's	H	Tramadol / Morphine	60-89	Int

For peer review only

23	13	M	70's	H	Liquid morphine and Codeine phosphate	0-29	UC
24	16	M	40/*50*	(S) Still on	Oxycodone changed to fentanyl unknown 12m ME	90-119	UC
25	16	F	80	?L	Buprenorphine patches	30-59	Int
26	13	M	70	L	Morphine + ?	60-89	Int
27	19	F	50's	H	Fentanyl patches	150+	UC
28	18 NE	F	70s	H	Morphine + liquid morphine	150+	UC
29	17 NE	F	50/60	L	Codeine phosphate and fentanyl patches	150+	Int
30	22 NE	M	60/70	L	Tramadol	0-29	Int
31	20	F	60's	0	Fentanyl patches	90-119	Int
32	21	F	80	L	Oxycodone	60-89	Int
33	21	F	80's	Lower by 2/3	Buprenorphine patch	30-59	UC
34	20	F	60's	H(? S)	Hydromorphone ??	150+??	Int
35	20	M	50's	sl L	Tramadol	30-59	UC
36	26 NE	M	70's	L (or 0)	??Oxycodone and liquid morphine	150+	Int
37	26 NE	F	50s	S	Tramadol	60-89	UC
38	24	F	70's	H sl	Tramadol	0-29	Int
39	27	F	50s	L (or S)	Buprenorphine to Morphine	30-59	UC
40	23 NE	F	60s	0 (or L)	Tramadol to Codeine phosphate	0-29	UC
41	29 NE	F	60s	L sl	Fentanyl patches ??liquid morphine	??	Int

Legend: * H=higher S=Same L=lower 0 = no opioids
**Baseline morphine equivalent bandings: 0-29mg,30-59mg,60-89mg,90-119mg, 120-149mg, 150mg+
***Allocation: UC- Usual Care, Int-Intervention, ITT-Intention to treat (allocated to Intervention but not exposed to intervention)

Comparison of Usual care and Intervention

Usual care Total 20	7NE 13 Mids	7M 13F	30s to 80s	5H 5S 7L 30	Range of patches tablets and liquid	0-29(8),30-59(4),60-89(3), 90-119(2), 120-149(0), 150+(3)	UC

For peer review only

Intervention<br>Total 20	8 NE<br>12 Mids	8M 12F	50s to<br>80s	4H 1S<br>9L 60	Range of patches<br>tablets and liquid	0-29(5),30-59(5),60-89(4),<br>90-119(1),120-149(0),150+(4),1 unknown	Int

Commented [NV1]: Do we need this in?

For peer review only

Supplementary material 8 Baseline demographic characteristics of all randomised participants vs interviewed participants

Demographic characteristics	Total N=608	PE Interviewees N=40
**Age (years)**		
N	608	40
Mean (SD)	61.3 (12.9)	64.7 (11.9)
Median (IQR)	62.3 (53.0 – 70.7)	64.1 (58.5, 71.8)
Missing	0	0
**Gender**		
Male	242 (39.8%)	15 (37.5%)
Female	362 (59.5%)	25 (62.5%)
Other	1 (0.2%)	0 (0%)
Prefer not to say	0 (0.0%)	0 (0%)
Missing	3 (0.5%)	0 (0%)
**Ethnicity**		
White	585 (96.2%)	38 (95.0%)
Black Caribbean	6 (1.0%)	1 (2.5%)
Black African	1 (0.2%)	1 (2.5%)
Black Other	1 (0.2%)	0 (0.0%)
Indian	6 (1.0%)	0 (0.0%)
Pakistani	1 (0.2%)	0 (0.0%)
Bangladeshi	0 (0.0%)	0 (0.0%)
Chinese	0 (0.0%)	0 (0.0%)
Prefer not to say	1 (0.2%)	0 (0.0%)
Other	4 (0.7%)	0 (0.0%)
Missing	3 (0.5%)	0 (0.0%)
**Employment status**		
Employed	132 (21.7%)	6 (15.0%)
Unemployed	14 (2.3%)	0 (0.0%)
At school or full time education	1 (0.2%)	0 (0.0%)
At school or part time education	1 (0.2%)	0 (0.0%)
Unable to work due to long term sickness	154 (25.3%)	13 (32.5%)
Looking after home/family	13 (2.1%)	0 (0.0%)
Retired from paid work	270 (44.4%)	21 (52.5%)
Other	20 (3.3%)	0 (0.0%)
Missing	3 (0.5%)	0 (0.0%)
**Age left full time education**		
Did not receive formal education	2 (0.3%)	0 (0.0%)
Age 12 or less	1 (0.2%)	0 (0.0%)
Age 13 to 16	345 (56.7%)	21 (52.5%)
Age 17 to 19	135 (22.2%)	6 (15.0%)
Age 20 or over	109 (17.9%)	12 (30.0%)
Still in full time education	4 (0.7%)	0 (0.0%)
Other	9 (1.5%)	1 (2.5%)
Missing	3 (0.5%)	0 (0.0%)
**How long have you experience pain**		
Less than 1 year	8 (1.3%)	0 (0.0%)
1-5 years	97 (16.0%)	9 (22.5%)
More than 5 years	500 (82.2%)	31 (77.5%)
Missing	3 (0.5%)	0 (0.0%)
**How long have you been taking opioids for your chronic pain**		
Less than 1 year	29 (4.8%)	0 (0.0%)
1-5 years	211 (34.7%)	20 (50.0%)
More than 5 years	365 (60.0%)	20 (50.0%)
Missing	3 (0.5%)	0 (0.0%)

For peer review only

Supplementary material 9 Participant feedback form findings

Questions	Responses			
**Q1 Were the aims of the course made clear?**	Out of 27 responses (4 missing) : 26 Yes, 1 No			
**Q2 What were the three most useful things on this course?**	**Theme***26 responses (ranked but not all filled in all three).*	**1st**	**2nd**	**3rd**
	Appreciation of the information	8	7	3
	Being in a group – meeting and interacting	8	8	2
	Supported by facilitators	3	3	1
	Lay facilitator input	3	1	0
	Techniques taught in course which were helpful (all different)	3	3	0
	Motivational aspects	1	0	4
	Support of GP	0	0	3
**Q3 What three things would you suggest to make this course better for future participants?**	Out of 24 responses, six suggested to keep it as it is. The overall suggestions from the remainder were disparate. Some wanted more time for discussion (but one wanted shorter sessions due to comfort), more contacts with staff, more meetings especially at end of entire course. Practical issues specific to venue e.g. closeness, parking, one toilet. Two suggested CDs should also be in MP3/4 format. Specific issues: One felt the App advice was unrealistic, one didn't like the meditation, one didn't like the self-help booklet manual, one hadn't used CDs.			

	**Very confident**	**Confident**	**Not very confident**	**Not confident at all**
**Q4: How confident do you feel that the course content will help you personally?**	14	11	1	4
**Q5: How confident do you feel that you will be able to use this in the future?**	16	10	0	1
	**Very good**	**Good**	**Satisfacto ry**	**Poor**
**Q6: Overall were the facilitators?**	23	4	1	0
**Q7: Overall were the handouts?**	15	12	1	0
	**Very useful**	**Useful**	**Not very useful**	**Not useful at all**
**Q8: How did you find the face to face meeting with the nurse?**	22	5	1	0
**Q9: How did you find the telephone calls with the nurse?**	18	9	1	0
**Q10: Overall how useful did you find the whole course?**	22	5	0	1

Q11: Is there anything else you'd like to say? 21 responses to an open comments box. Fifteen positive, 2 negative ('course was a waste of time' and that the approach had not worked for them) and three mixed. Indicative quotes:

"I am now completely off tramadol and despite still going through some nasty withdrawal symptoms I am so glad I had the opportunity to attend the course. I didn't realise how bad the drug is and how ineffective it is for long term chronic pain. I am looking forward to getting my life back."

"I would recommend this course to anyone who has a chance to attend it. It has helped me to come off one of my painkillers, to be more outgoing, to talk about my problems more and given me more confidence when in a group of people."

Supplementary material 10 Summary of intervention attendance data

	Self-management (intervention)
**Group session attendance[1,2]**	
Number randomised to intervention	305
Session 1 only	13 (4.3%)
Session 1 and 2	17 (5.6%)
Session 1 and 3	10 (3.3%)
Session 1, 2 & 3	166 (54.4%)
Attended no sessions	90 (29.5%)
Missing	9 (3.0%)
**Group size at randomisation**	
N	35
Mean (SD)	8.71 (2.9)
Median (IQR)	9 (5,11)
Missing	0 (0%)
**Group size at Session 1[3]**	
N	35
Mean (SD)	6.24 (2.82)
Median (IQR)	7 (3, 8)
Missing	2 (5.7%)
**Face to Face interviews**	
Attended first F2F interview	190 (62.3%)
Attended both F2F interviews	131 (42.9%)
Missing	15 (4.9%)
**Telephone interviews**	
Attended first telephone session	167 (54.8%)
Attended both telephone sessions	152 (49.8%)
Missing	34 (11.1%)
**Compliance**	
Number of participants with full compliance	144 (47%)
Number of participants with at least minimal compliance	190 (62%)
Number with less than minimal compliance	115 (38%)

Legend: 161/305 participants achieved minimal compliance by attending at least Day 1 and the first one-to-one consultation.

[2] 144/305 participants achieved full compliance by attending at least Day 1, 2 & 3, the first one-to-one consultation and 1 telephone call.

[3] 6 participants who attended day 1 attended groups they were not randomised to. This has been summarised by groups they attended, not randomised too.

For peer review only

Supplementary material 11 Fidelity scores of group sessions in percentages

Session	Adherence *(italics 10% check)*			Totals	Competence			Totals
	**Early**	**Mid**	**Late**		**Early**	**Mid**	**Late**	
Day 1 session 2	100	81	25 *(25)*		92	64	0 *(0)*	
Day 1 session 3	94 *(94)*	100	44		90 *(87)* agreed 88	86	42	
Day 1 session 4	100	75	88		70	71	67	
Day1 session 7	72	89	83		80	75	90	
Day 1 session 8	100	88	81		100	60	75	
Day 2 session 13	77	88 *(90)* agreed 89	84		57	100 *(100)*	100	
Day 2 session 14	56 *(66)* agreed 61	100	94		58*(43)* agreed 50	92	90	
Day 2 session 16	79	88	82		50	70	100	
Day 3 session 21	88	100	89		80	100	100	
Day 3 session 22pt 1	93	86	64		70	92	83	
Day3 session 23	90	73	91		100	100	100	
**Average**	**86.72**	**88.09**	**75.00**	**83.27%**	**76.09**	**82.73**	**77**	**78.61%**
**Range**	**61-100**	**73-100**	**25-94**	**25-100%**	**50-100**	**60-100**	**0-100**	**0-100%**
**Median**	**90**	**88**	**83**	**88**	**80**	**86**	**90**	**86**

For peer review only

Supplementary material 12 Fidelity scores of one-to-one nurse consultations

Timepoint Early	Group ID	Adherence Score	Competence Score
1st	1	100%	100%
2nd	2	93% *(93%)*	100% *(100%)*
1st	3	61%	67%
2nd	3	86%	83%
1st	4 NE	100%	100%
2nd	4 NE	100%	100%
1st	5 NE	100%	100%
2nd	5 NE	100%	100%
2nd	6	100%	100%
1st	7 NE	67%	100%
1st	8	100%	100%
1st	9 NE	83% *(94%)*94	100% *(83%)*92
2nd	10	88%	83%
2nd	10	100%	100%
**Early averages**		**92.07%**	**94.64%**
**Range**		**61 to 100**	**67 to 100**
**Timepoint Mid**	**Group ID**	**Adherence**	**Competence**
1st	11	100%	100%
1st	12 NE	94%	100%
2nd	12 NE	100%	100%
1st	13	89%	92%
1st	14 NE	61% *(61%)*	50% *(50%)*
1st	15	94%	100%
1st	15	94%	100%
2nd	20	71%	83%
**Mid averages**		**87.87%**	**90.63%**
**Range**		**61 to 100**	**50 to 100**
**Timepoint Late**	**Group ID**	**Adherence Score**	**Competence Score**
1st	21	100%	92%
2nd	21	93%	100%
1st	24	94%	100%
2nd	24	86% *(86%)*	100% *(100%)*
1st	27	89%	83%
**Late averages**		**92.4%**	**95%**
**Range**		**86 to 100**	**83 to 100**
**Total averages**		**90.78%**	**93.42%**
**Range**		**61 to 100**	**50 to 100**

Legend: NE - North East region

For peer review only

Supplementary material 13

Baseline change mechanism questions of all randomised participants by treatment group

		Control N=303	Intervention N=305	TOTAL N=608
**1.I want to reduce my opioid use**				
Motivation	Not at all	25 (8%)	21 (7%)	46 (8%)
	By a little	45 (15%)	37 (12%)	82 (14%)
	By Half	36 (12%)	44 (14%)	80 (13%)
	So I only use a little	60 (20%)	95 (31%)	155 (26%)
	So I use no opioids	133 (44%)	102 (33%)	235 (39%)
	Missing	4 (1%)	6 (2%)	10 (2%)
**2.I expect in 4 months' time, I will have reduced my opioid use**				
Expectations	Not at all	45 (15%)	43 (14%)	88 (15%)
	By a little	78 (26%)	82 (27%)	160 (26%)
	By Half	56 (19%)	56 (18%)	112 (18%)
	So I only use a little	67 (22%)	82 (27%)	149 (25%)
	So I use no opioids	50 (17%)	37 (12%)	87 (14%)
	Missing	7 (2%)	5 (2%)	12 (2%)
**3.I am confident I could reduce my opioid use a lot over 4 months**				
Self-efficacy	Not at all confident	90 (30%)	90 (30%)	180 (30%)
	Somewhat confident	70 (23%)	77 (25%)	147 (24%)
	Fairly confident	79 (26%)	79 (26%)	158 (26%)
	Strongly confident	35 (12%)	40 (13%)	75 (12%)
	Completely confident	22 (7%)	15 (5%)	37 (6%)
	Missing	7 (2%)	4 (1%)	11 (2%)
**4.I feel that involvement in this study can help me to reduce my opioid use**				
Perceived credibility of intervention	Not at all	25 (8%)	22 (7%)	47 (8%)
	By a little	76 (25%)	73 (24%)	149 (25%)
	By Half	38 (13%)	46 (15%)	84 (14%)
	So I only use a little	68 (22%)	86 (28%)	154 (25%)
	So I use no opioids	86 (28%)	69 (23%)	155 (26%)
	Missing	10 (3%)	9 (3%)	19 (3%)

For peer review only

Supplementary material 14

Change mechanism question data from 4, 8 and 12 month follow up

**4-month follow-up change mechanism questions of all randomised participants by treatment group**	**Control**	**Intervention**
**I want to reduce my opioid use (If still on opioids)**		
Number still on opioids	194	166
Not at all	20 (10.3%)	18 (10.8%)
By a little	23 (11.9%)	11 (6.6%)
By Half	32 (16.5%)	11 (6.6%)
So I only use a little	29 (14.9%)	31 (18.7%)
So I use no opioids	40 (20.6%)	57 (34.3%)
Missing	50 (25.8%)	38 (22.9%)
**I feel that involvement in this study has helped me to reduce my opioid use**		
Number still on opioids	159	192
Not at all	82 (51.6%)	30 (15.6%)
By a little	43 (27.0%)	24 (12.5%)
By Half	10 (6.3%)	21 (10.9%)
So I only use a little	6 (3.8%)	33 (17.2%)
So I use no opioids	8 (5.0%)	79 (41.1%)
Missing	10 (6.3%)	5 (2.6%)
**8-month follow-up change mechanism questions of all randomised participants by treatment group**	**Control**	**Intervention**
**I want to reduce my opioid use (If still on opioids)**		
Number still on opioids	152	136
Not at all	25 (16.4%)	20 (14.7%)
By a little	25 (16.4%)	16 (11.8%)
By Half	24 (15.8%)	9 (6.6%)
So I only use a little	25 (16.4%)	26 (19.1%)
So I use no opioids	36 (23.7%)	45 (33.1%)
Missing	17 (11.2%)	20 (14.7%)
**I feel that involvement in this study has helped me to reduce my opioid use**		
N	149	181
Not at all	68 (45.6%)	27 (14.9%)
By a little	41 (27.5%)	27 (14.9%)
By Half	16 (10.7%)	20 (11.0%)
So I only use a little	9 (6.0%)	28 (15.5%)
So I use no opioids	12 (8.1%)	74 (40.9%)
Missing	3 (2.0%)	5 (2.8%)
**12-month follow-up change mechanism questions of all randomised participants by treatment group**	**Control**	**Intervention**
**I want to reduce my opioid use (If still on opioids)**		
Number still on opioids	193	160
Not at all	20 (10.4%)	18 (11.3%)
By a little	22 (11.4%)	10 (6.3%)
By Half	27 (14.0%)	15 (9.4%)
So I only use a little	36 (18.7%)	32 (20.0%)
So I use no opioids	38 (19.7%)	49 (30.6%)
Missing	50 (25.9%)	36 (22.5%)
**I feel that involvement in this study has helped me to reduce my opioid use**		
N	160	188
Not at all	73 (45.6%)	26 (13.8%)
By a little	39 (24.4%)	25 (13.3%)
By Half	17 (10.6%)	18 (9.6%)

For peer review only

So I only use a little	10 (6.3%)	44 (23.4%)
So I use no opioids	18 (11.3%)	72 (38.3%)
Missing	3 (1.9%)	3 (1.6%)

For peer review only

Supplementary material 15

Involvement in study reducing opioid use at 4, 8 and 12 months allocation comparison

**I feel that involvement in this study has helped me to reduce my opioid use:** Not at all, by a little, by half, so I only use a little, so I use no opioids						
**Grouping into 3 bands**	**Control 4month follow up**	**Intervention 4month follow up**	**Control 8month follow up**	**Intervention 8month follow up**	**Control 12month follow up**	**Intervention 12month follow-up**
N	159	192	149	181	160	188
Opioids reduced not at all + by a little	125 (79%)	54 (28%)	109 (73%)	54 (30%)	112 (70%)	51 (26%)
By half	10 (6%)	21 (11%)	16 (11%)	20 (11%)	17 (11%)	18 (10%)
Opioids reduced so I only use a little + so I use none	14 (9%)	112 (58%)	21 (14%)	102 (56%)	28 (18%)	116 (62%)
Missing	10 (6%)	5 (3%)	3 (2%)	5 (3%)	3 (2%)	3 (2%)

For peer review only

BMJ Open

"It was a joint plan we worked out together." How the I-WOTCH programme enabled people with chronic non-malignant pain to taper their opioids: a process evaluation

Journal:	*BMJ Open*
Manuscript ID	bmjopen-2023-074603.R1
Article Type:	Original research
Date Submitted by the Author:	25-Sep-2023
Complete List of Authors:	Nichols, Vivien; University of Warwick, Warwick Clinical Trials Unit<br>Abraham, Charles; Deakin University<br>Eldabe, Sam; The James Cook University Hospital<br>Sandhu, Harbinder; University of Warwick Warwick Medical School, Clinical Trials Unit<br>Underwood, Martin; Warwick University, Warwick Medical School<br>Seers, Kate; University of Warwick, Warwick Medical School
**Primary Subject Heading**:	Qualitative research
Secondary Subject Heading:	Health services research
Keywords:	Chronic Pain, PAIN MANAGEMENT, QUALITATIVE RESEARCH, Patient-Centered Care

SCHOLARONE™
Manuscripts

I, the Submitting Author has the right to grant and does grant on behalf of all authors of the Work (as defined in the below author licence), an exclusive licence and/or a non-exclusive licence for contributions from authors who are: i) UK Crown employees; ii) where BMJ has agreed a CC-BY licence shall apply, and/or iii) in accordance with the terms applicable for US Federal Government officers or employees acting as part of their official duties; on a worldwide, perpetual, irrevocable, royalty-free basis to BMJ Publishing Group Ltd ("BMJ") its licensees and where the relevant Journal is co-owned by BMJ to the co-owners of the Journal, to publish the Work in this journal and any other BMJ products and to exploit all rights, as set out in our licence.

The Submitting Author accepts and understands that any supply made under these terms is made by BMJ to the Submitting Author unless you are acting as an employee on behalf of your employer or a postgraduate student of an affiliated institution which is paying any applicable article publishing charge ("APC") for Open Access articles. Where the Submitting Author wishes to make the Work available on an Open Access basis (and intends to pay the relevant APC), the terms of reuse of such Open Access shall be governed by a Creative Commons licence – details of these licences and which Creative Commons licence will apply to this Work are set out in our licence referred to above.

Other than as permitted in any relevant BMJ Author's Self Archiving Policies, I confirm this Work has not been accepted for publication elsewhere, is not being considered for publication elsewhere and does not duplicate material already published. I confirm all authors consent to publication of this Work and authorise the granting of this licence.

For peer review only

"IT WAS A JOINT PLAN WE WORKED OUT TOGETHER." HOW THE I-WOTCH PROGRAMME ENABLED PEOPLE WITH CHRONIC NON-MALIGNANT PAIN TO TAPER THEIR OPIOIDS: A PROCESS EVALUATION

Authors

Vivien P Nichols
Warwick Clinical Trials Unit,
Warwick Medical School,
University of Warwick, Coventry, UK

Charles A Abraham
Professor of Psychology
School of Psychology, Deakin University, Geelong.
Australia

Sam Eldabe
Department of Pain Medicine,
The James Cook University
Hospital, Middlesbrough, UK

Harbinder K Sandhu
Warwick Clinical Trials Unit,
Warwick Medical School,
University of Warwick, Coventry, UK

Martin Underwood
Warwick Clinical Trials Unit,
Warwick Medical School,
University of Warwick, Coventry, UK
University Hospitals of Coventry and Warwickshire
Coventry, UK

Kate Seers corresponding author
Warwick Research in Nursing,
Division of Health Sciences,
Warwick Medical School,
University of Warwick, Coventry, UK

Kate.Seers@warwick.ac.uk

On behalf of the I-WOTCH team see acknowledgements

For peer review only

Abstract

Background

The I-WOTCH randomised controlled trial found that a group based educational intervention to support people using strong opioids for chronic non-malignant pain helped a significant proportion of people to stop or decrease opioid use with no increase in pain-related disability. We report a linked process evaluation of the group-based intervention evaluated in comparison to a usual-care control group who received a self-help booklet and relaxation CD.

Methods

We interviewed 18 intervention facilitators, and 20 intervention and 20 control participants who had chronic non-malignant pain and were recruited from general (family) practices in the UK. Quantitative data included change-mechanism questions on the trial questionnaires which explored motivation, expectations, and self-efficacy. Fidelity was assessed by listening to a sample of audio-recorded group sessions and nurse consultations. Quantitative and qualitative data were integrated using 'follow a thread' and a mixed methods matrix.

Findings

Four overarching themes emerged: (i) the right time to taper, (ii) the backdrop of a life with chronic pain, (iii) needing support and (iv) the benefits of being in a group. Delivery fidelity was good, adherence (83%) and competence (79%) across a range of intervention groups. Staff delivering the intervention found three typical responses to the intervention: resistance, open to trying, and feeling it was not the right time. The group experience was important to those in the intervention arm. It provided people with a forum in which to learn about the current thinking about opioid usage and its effects. It also gave them examples of how feasible or personally relevant coming off opioids might be.

Conclusion

The process evaluation data showed that the I-WOTCH intervention was well delivered, well received and useful for most interviewees. Being "the right time" to taper and having support throughout tapering, emerged as important factors within the context of living with chronic pain.

For peer review only - http://bmjopen.bmj.com/site/about/guidelines.xhtml

Key words: Opioid, process evaluation, chronic non-malignant pain, pain self-management, tapering, behaviour change, complex intervention.

For peer review only

Strengths and Limitations

- The success of I-WOTCH was demonstrated from multiple perspectives providing an insight into the acceptability, replicability, and transferability of the intervention.
- Enablers of, and barriers to, the tapering process were explored, both of which are valuable for tailoring of this type of intervention to the individual.
- This protocol-based process evaluation showed good fidelity of delivery, at least minimal compliance in 62%, and a very positive experience of the intervention, suggesting its suitability for service rollout.
- Although General Practitioners screened patients and prescribed for tapering, their perspectives were not a part of this process evaluation but may have provided useful information for future implementation.
- As 95% of participants in the process evaluation were white, the reach of the study was thus limited and given interviews were 12 months after the intervention, recall bias may be a limitation.

This work was supported by the National Institute for Health and Care Research (NIHR) Health Technology Assessment (HTA) project number 14/224/04

Trial registration number: ISRCTN49470934

For peer review only

Background

Embedded process evaluations of complex interventions in randomised controlled trials are critical to increase understanding of how and why interventions may or may not work, as well as how practical interventions are when implemented in service contexts. They increase confidence in trial results and provide guidance on their implementation [1,2].

In the landmark I-WOTCH (Improving the Wellbeing of people with Opioid Treated CHronic pain) trial (n=608) we demonstrated that a self-management support intervention helps one in five people using strong opioids become opioid free at one year: 29% intervention vs 7% control, odds ratio 6.55 (95% confidence interval 3.42 to 12.55) with no increase in pain interference.

The I-WOTCH intervention included educational, behavioural, and psychological components, combining group and one to one support. Papers reporting the intervention development, the trial protocol, and the main results, and process evaluation protocol have been published [3-6]. Here we report the I-WOTCH process evaluation, specifically.

- Experiences of the I-WOTCH intervention(s): including enablers of, and barriers to, change among participants.
- Implementation of the I-WOTCH group intervention: exploring the dose delivered and received, and the fidelity of delivery.
- Change mechanisms potentially underpinning intervention effects.
- Contextual issues: exploring how these may affect the outcome or running of the study and/or intervention.

For peer review only

For peer review only - http://bmjopen.bmj.com/site/about/guidelines.xhtml

Methods

We explored experiences of the participants and intervention delivery staff as well as key areas of process evaluation; context (contextual factors which may affect the implementation), fidelity (whether the intervention was delivered as conceived), dose delivered (the amount of the intervention delivered) and dose received (the amount of the intervention received by participants) [7]. The group sessions took place once a week for three weeks. There was a face-to-face consultation between the second and third group sessions. After the third group session, there were two telephone conversations and then a final face to face consultation over a total period of 9-10 weeks. The process evaluation ran alongside the main study from May 2017 to March 2020.

The I-WOTCH trial recruited participants with chronic non-malignant pain from general (family) practices from midlands and north-east of England (Table 1). Inclusion and Exclusion criteria are listed in Box 1. Once randomised all participants received a self-help booklet 'My Opioid Manager' and a relaxation CD. The intervention group also received a three-day group intervention, run by a specially trained nurse/other health care professional and lay person with chronic pain who had tapered their own opioids, with two additional face to face nurse consultations and telephone support.

*Box 1: **I-WOTCH participant inclusion and exclusion criteria***

Inclusion criteria	Exclusion criteria
• Provision of written informed consent<br>• Aged 18 years old or above<br>• Using opioids for chronic non-malignant pain<br>• Using strong opioids for at least 3 months<br>• Using strong opioids on most days in the preceding month<br>• Fluent in written and spoken English<br>• Able to attend group sessions<br>• Willing for GP to be informed of participation	• Regular use of injected opioid drugs<br>• Chronic headache as the dominant painful disorder<br>• Serious mental health problems that preclude participation in a group intervention<br>• Previous entry or randomisation in the present trial<br>• Participation in a clinical trial of an investigational medicinal product in the last 90 days<br>• Pregnant at time of eligibility assessment, or actively trying to become pregnant<br>• People receiving strong opioid for the management of pain due to active malignant disease

We used process evaluation theory to devise a logic model specific to this intervention and its theoretical underpinnings, informed by The Information, Motivation and Behaviour Skills Model [8-9] which highlights

For peer review only - http://bmjopen.bmj.com/site/about/guidelines.xhtml

For peer review only

that knowledge, strong and stable motivation, and prerequisite skills are needed to initiate and maintain behaviour change (supplementary material 1 – logic model). We used a mixed methods approach for the process evaluation (Table 1).

Table 1 I-WOTCH process evaluation: Aims, Key Components, Methods, Analyses and Data Type

Aims Addressed	Key Components	Methods	Analysis	Data Type
1) **Experiences** of being in the I-WOTCH study	Participant interviews	Semi-structured, face-to-face at home or convenient location, after they had completed the post 12-month questionnaire for the main study.<br>Purposively sampled to attain a range across; age, opioid reduction experience, location and gender across the intervention and control arms. n=20 intervention & n=20 control group.	Framework Analysis[10]	Qualitative
1) **Experiences** of delivering the I-WOTCH Intervention	Intervention delivery staff interviews	Semi-structured, face-to-face at workplace or convenient location or telephone. After all intervention groups were completed. Pragmatic sample of Nurse and Lay facilitators– all invited. n=18	Framework Analysis [10]	Qualitative
1) **Experiences** of attending the group components of the intervention	Participant feedback forms	Paper form given to last 10 groups after day 3, asked to complete and send back in trial return prepaid envelope. Open and closed questions. n=31	Thematic Analysis [11]	Qualitative & Quantitative
2) Intervention **Implementation**	Dose delivered	Trial data describing how many group sessions were conducted in each location.	Descriptive statistics, charts, tables or figures using STATA 17.0. The mean and standard deviation were presented for continuous data, and the frequency and percentage for categorial data, summarised by treatment arm.	Quantitative
2) Intervention **Implementation**	Dose received	Trial data of attendance and attrition.<br>Uptake of the one-to-one consultations and follow up telephone calls. (It was not possible to record telephone conversations).	Descriptive statistics, charts, tables or figures using STATA 17.0. The mean and standard deviation presented for continuous data, and the frequency and percentage for categorial data, summarised by treatment arm.	Quantitative
2) Intervention **Implementation**	Fidelity	All intervention groups and one-to-one nurse consultations audio recorded.<br>A sample of recordings listened to and assessed for adherence and competence.	Descriptive statistics - Adherence and competency scores summed, and percentage score calculated.	Quantitative

For peer review only - http://bmjopen.bmj.com/site/about/guidelines.xhtml

3) **Potential effects of change mechanisms**	Change mechanism questions	Trial data collected from four questions about participant motivation, expectations, self-efficacy and perceived credibility of the intervention in the main trial questionnaire.	Descriptive statistics, charts, tables or figures using STATA 17.0. The mean and standard deviation (sd) presented/ continuous data, and the frequency and percentage / categorial data, summarised by treatment arm.	Quantitative
4) **Contextual Issues**	Context	Contextual factors were considered across all the data	Thematic Analysis	Qualitative and Quantitative
**(1,2,3 and 4)**	Synthesis of the data	Mixed methods approach combining all the data to produce a model of Overarching Themes.	Use of a 'mixed methods matrix' and 'following a thread' analysis strategy. O'Cathain et al[12]	Qualitative & Quantitative

Interviews

Potential interviewees (participants and staff) were sent an information leaflet. Those interested gave informed consent to be interviewed. Intervention delivery staff were told of the interviews at their training and were approached on completing their final group. Semi-structured interviews were recorded using an encrypted device (OLYMPUS DS-7000 digital voice recorder) see supplementary material 2 for indicative topic guide. Recordings were transcribed verbatim, anonymised, given numerical identifiers to maintain confidentiality and checked for accuracy by the research interviewer (VN). The data were analysed using NVivo 12 software to explore and organise the data. Warwick CTU's lone worker policy, including a risk assessment, was followed for interview visits.

All data were collected and stored in digitally secure locations with restricted access in accordance with the Data Protection Act 2018c12 which is the UK's implementation of the General Data Protection Regulation (GDPR). [13]

Participant Feedback forms

We gave feedback forms to the last 10 groups of participants after completing day three of the intervention. Questions asked about different aspects including how the group intervention was delivered and what they had found personally useful/not useful.

For peer review only

Dose delivered and dose received.

We collated trial data including date, location, and attendance of the intervention groups to determine the dose delivered and received.

Fidelity

Our fidelity assessment is detailed in our protocol paper and supplementary material 3 shows our random sampling [5]. We listened to audio recordings of 11 prespecified, intervention group sessions across days one, two and three (those which were educational and promoted discussion see supplementary material 4) to assess adherence to the manual and competence of delivery. Other more practical sessions were checked for content presence but not rated in terms of delivery competence. We used bespoke assessment forms for group and individual sessions across a range of facilitators. Adherence items were scored as taking place: Yes (2), Partially (1) or No (0). Competence items were scored as; Evident (2) Partially evident (1) or Not evident (0) (see supplementary material 5). Scores were summated and averages calculated for adherence and competence of each session.

A random 10% of first and second one to one nurse/patient consultation recordings were also assessed throughout the study. If audio-recordings were missing or inaudible, then the same sessions as those missing would be taken from the next group or one to one consultation in the same area (northeast or midlands). A second researcher (KS) double coded 10% of all the assessments.

Potential Change Mechanisms

Four change mechanism questions about; patient motivations, expectations, self-efficacy, and perceived credibility of the intervention were added to the main trial questionnaire to ascertain if any of these factors had an effect on opioid reduction (Supplementary material 6).

The Process Evaluation team

The process evaluation team included; KS a co-applicant and work package lead who has a long track record in qualitative and mixed methods research in complex interventions, CA a co-applicant, highly experienced in behaviour change research and process evaluations and VN a research fellow with a background in physiotherapy, experienced in mixed methods in rehabilitation RCTs. The team collated data and analysed it

For peer review only - http://bmjopen.bmj.com/site/about/guidelines.xhtml

separately from the main study. The initial report was given to the senior and chief investigators SE, HS and MU prior to the results from the main trial being available.

Patient and Public Involvement Statement

PPI input was not used directly with this process evaluation although one lay advisor, recruited via UNTRAP (Universities/User Teaching and Research Action Partnership) who helped with the development of I-WOTCH and its intervention, stayed on to become a valuable team member. Study findings will be disseminated via newsletters and a lay summary put onto the study website as well as feedback to UNTRAP and other partnerships as outlined in the I-WOTCH protocol paper [5].

For peer review only

For peer review only - http://bmjopen.bmj.com/site/about/guidelines.xhtml

Findings

1) Experiences of the I-WOTCH interventions

We had multiple sources of qualitative data (Figure 1).

Figure 1 – Qualitative data sources

Participant interviews

The 40 interviewees (20 Control/20 intervention) had a mean age of 65, range 59-72 years, 38/40 (95%) were white and 25 (63%) were female. Half (50%) had been on opioids for >5 years and 31 (78%) had pain for >5 years. See supplementary material 7 and 8 for full interviewee characteristics and compared to the main study demographics, noting the interview sample were broadly consistent with the main study. Eighteen participants declined an interview mostly because of ‘health issues’ (e.g. appointments or operations) or ‘not a good time’ (e.g. work commitments, too busy).

Each interviewee has been given a numerical identifier, which follows each presented quotation. Table 2 provides evidence from the control interviews.

Control Interviews

The majority (14/20) found the self-help booklet informative, useful for further resources they could access. Three did not remember receiving the self-learning manual and two found some of it difficult to understand.

Table 2 Control exemplar quotes

**Aspects of Control Intervention**	**Exemplar quotes**
Some spoke about the information being a potential trigger to start the tapering process and that ‘it made you think’:<br>Some found the information about risks and possible side effects interesting or shocking.	*‘…to make you think about what you’re taking and why you’re taking it’.08.*<br>*‘…I was never told about the constipation problems that it would cause and obviously you read about the side-effects in the little leaflets and then it’s put away!’ 20*<br>*‘sleep apnoea… …that was a bit scary..’ 23*
A few interviewees used the self-help booklet to start tapering.	*‘I just followed everything it was doing…I just came down off the pills…I’ve done more exercise…’ 15*
A few didn’t find it as useful:	*‘‘…in a way it’s assuming that you’re overweight and you don’t exercise!......it didn’t tell me what I could do if I wasn’t overweight…and I’m getting loads of exercise…… It doesn’t really tell you what you can do as an alternative…28*

For peer review only - http://bmjopen.bmj.com/site/about/guidelines.xhtml

Most (n=10) listened to the CD once or twice and did not find it useful. Another four who used it more than once or twice said that ultimately it wasn't useful for them. However, five participants used it to good effect over the trial period, some on a regular basis and others 'as and when' they felt they needed it.

Enablers and barriers to tapering

In the interview schedule we asked all interviewees about enablers and barriers to tapering. We analysed the data from all 40 patient interviews using framework analysis for enablers and barriers to the tapering process including any attribution to the I-WOTCH study from the participants' perspectives.

As we are focussing on the I-WOTCH intervention for this paper, we illustrate the themes with quotations from the intervention arm only for clarity.

Enablers

We identified four themes; (i) Readiness to start tapering, (ii) I-WOTCH as a trigger or motivator, (iii) Continuing to taper (once they had started), and (iv) Living without opioids. Subthemes are listed in Table 3 with exemplar quotes from the intervention participants.

For peer review only - http://bmjopen.bmj.com/site/about/guidelines.xhtml

Table 3abcd Enablers to Tapering Opioids

3a Theme 1: Readiness to Taper	
**Theme 1 sub themes**	**Exemplar quotes (with participant number) – intervention**
Already made the decision or had started to taper	*and then the invitation to take part in the study so I was reasonably invested in actually engaging with the study because it was something that I'd been thinking of myself… if you understand… 29*
Didn't like the side effects	*so it was a case of well I would try and see if I could reduce them or come off them and see if I wouldn't need as much other medication so it's the side-effect medication so I wouldn't be taking the anti-nausea tablets I wouldn't be having the… mmm… bowel medication…34*
Not wanting to be on opioids	*… it was when it was on the tele as well about being an opioid I thought I don't want to be addicted or to be totally dependent on them…13*
Wondering if opioids were working anyway	*'Do you really need to be on this now?' ……so I was reasonably invested in actually engaging with the study because it was something that I'd been thinking of myself 29*
Support from GP or family to taper	*…I myself thought right I don't want to be on these anymore and the doctor agreed…25*
Positive past experience of tapering	*16 – 17 years ago just after I'd had my first pair of hip replacements before they started giving me problems I came straight off all the opioids I was taking then so I knew that I didn't really have a problem with… withdrawal from a previous experience…22*
The right time to fit with life events	*I'm trying to think back to that part of the summer… how I was like… think I was relatively well which is why I started reducing it…34*

3b Theme 2: I-WOTCH as a trigger or motivator to taper	
**Theme 2 sub themes**	**Exemplar quotes - intervention**
I-WOTCH as trigger	*… but being part of the study has… mmm… enabled me not to be on them anymore whereas I don't know whether that would've been the case otherwise!29*
Information	*… and then when I was on the course I realised how damaging these things could be and I got all this information off the ladies and went through that …. well there's only one thing you've got to do… you've got to come off these damn things…30*
Support from group or nurse	*…I think because I had the support …. I just felt encouraged like that I could do it meself so I liked the support I suppose and the fact that they believed in me…13*
Tools to help	*I was over the moon because I got more tips you know… … it was all helpful to me. 10*
Tapering plan - given the means	*… it was a joint plan that we worked out together… 09*
Open Mindset 'give it a go'	*… but I went in with an open mind and everything we did on the group the… the practice sessions and the things we got to do at home you know the colouring and everything else that we did on the study… mmm… and listening to the relaxation CD's and everything like that… I'm… I went in with an open mind on that and listened to it and took out what… what I could…..09*

3c Theme 3: Continuing to taper	
**Theme 3 sub themes**	**Exemplar quotes - intervention**
Self-efficacy	*… the Tramadol thing is still there I look at it at times and I sort of say 'maybe it's better that it's there instead of me throwing it away because that's training me' it's giving me enough courage to sort of say 'I'm straight I can actually see it and overcome it' and… err… I'm almost like it's like a mini training moulding myself on how to… to… to manage that…38*
Feeling better, decreased side effects	*but it was really important to me to get rid of this… rid myself really of this drug… all I can say is this complete… having from the other side now I describe as 'un-reality' 29*
Pain similar	*… but really I can say coming off of it the pain's not any worse than it was…31*
Seeing the reduction of opioids	*…so it just shows you… you just… you know if I can do without half of them I'm going to try and get down a bit further…21*

For peer review only - http://bmjopen.bmj.com/site/about/guidelines.xhtml

Flexibility (slow) of the tapering plan	*it was done so… so slowly and minutely it was… I… I can't honestly say I had any side-effects!31*
Participant in control	*I thought that was helpful you know to set your goals and know what you'd set 31*
Understanding/ weathering withdrawal effects	*I was kicking I was twitching I was… I couldn't sit still I was… Aah… me hands were going and… I'm not joking I had to run round… I was running the table or walking fast round the table 'cause I couldn't keep still… I couldn't get any comfort whatsoever or any satisfaction and I was like 'Jeeze' and a couple of times I went… thought shall I put a patch on but I no I've got to persevere because if I put a patch on I know it will stop but I am back to square one so no I've got to persevere… I've got to persevere, and I did and I carried on and that's it… eventually it dispersed and… great so I've never touched the patches since… it's really great I'm chuffed…05*
Support from group or nurse	*…and that's what was good about the course with the support and the support network …… but it's that conversation amongst yourselves because you all know you're in the same boat you know what I mean and a lot of ya will have… would've had similar experiences and similar thoughts and whatever it be… err… it's… it's that which I think motivates ya…22*

**3d Theme 4: Living without opioids**	
Living without opioids	*……because me body was getting itself put right if you understand where I'm at… …and in my mind I know I'm not abusing my body any more than what just for Paracetamols and everything I'm on now… 10*<br>*…there's negative points of coming off them but I feel the plus side is that these tablets… these opioids are not doing any harm to me body anymore…31*<br>*… because of the course you know the few weeks that we were on that was a great help to me it just urged me to get off you know… wean myself off them which I did! 25*

For peer review only

Barriers to tapering

Three barrier themes are from different phases of tapering; pre-tapering, during tapering and after stopping opioids. Subthemes are given in table 4abc below with exemplar quotes from the intervention.

Table 4abc Barriers to tapering

**4a Theme 1: Pre tapering**	
**Sub-themes**	**Intervention- exemplar quotes**
No intention: not receptive/ 'I'm not addicted'/ Not motivated	*… I think I'd be in a bad place if I couldn't take them… I certainly don't think I'm addicted to opioids 16*
No choice, no alternatives	*… I really don't want to be on them that's my wish but… mmm… it's taught me that I cannot be without them which is unfortunate….25*

Not the right time: Other health priorities / Won't fit in with life (holidays) / Awaiting procedures which may help or affect their pain e.g. knee operation/operative pain	*…… I'd not even considered that at all until… until I have the… my hip replaced and then that knee's done that hip's done me eyes are done then we can see… start tapering off maybe having one… one a day instead of two a day. 16*
Fear of: Increased pain / Decreased function / Not coping / Opioids being stopped / Having no future access to opioids	*'I remember thinking 'I'll be in so much pain and I'll be… I'll be even more angry with myself' so I was thinking, ' I really… I can't… I can't do this…21*
Resistance to group intervention messages: Anger at GPs- (Affecting GP/Patient relationship) / Scepticism	*… but I'm quite annoyed that a GP who's prescribing more and more and more and should ought to… jolly well ought to know the research background so… and there was a lot of anger in the room that I was in about that…29*<br>*but I was a little pessimistic… mmm… because of knowing how much pain I've been in… in the past and nothing else has worked…07*

**4b Theme 2: During Tapering**	
**Sub-themes**	**Intervention- exemplar quotes**
Increased pain	*I think I was wrong to come off them believe it or not… and I really want to get off them because that make one very constipated and I just wanted to be free of them but I couldn't manage it… Saturday and Sunday were dreadful days I was in such pain! 25*
Unpleasant withdrawal symptoms	*… but I'd say the symptoms were far worse when I reduced the second half …… … cramps in my legs and the… oh the hot sweats and cold sweats they went on for a long time that was horrible…01*
Tapering too fast	*… it's a shame that there wasn't another way or to have changed the patches in some way to have come down at a slower rate and perhaps then I may have had more success I think with coming down 25% because this particular patch that I'm on… the Butec one……it only comes in 5's…07*
GP unsupportive of the process or poor access	*…cos the doctor actually… err… didn't seem at all conversant with the idea…32*
Relatives worried not supportive of process	*… even my brother noticed he said 'you know it's been really rough for you coming off …07*
Missing trial support	*I know that I couldn't have done it without going into a group to begin with to have that information if someone had just sat me down and said ' you're doing to reduce this by this and this' I would be screaming I think… no, no I can't you know yeah.21*

**4c Theme 3: After stopping opioids**	
**Sub-theme**	**Intervention- exemplar quotes**
New or returned pain	*… I came completely off the opioids while I was doing the course… completely off them… erm… stopped them all… err… sadly I fell over in December… it caused me a load of problems I slipped a disc and whatever and hurt me left hip… err… so I went back on them to deal with the initial pain and if I'm completely honest with myself the pain's now bearable again but I've gone to that what I call my traditional crutch that I rely on…22*

For peer review only - http://bmjopen.bmj.com/site/about/guidelines.xhtml

The role of the general practitioner (GP)

Most interviewees spoke about their GP in relation to their opioid use and tapering and the role of GP is presented in Table 5. In the I-WOTCH study, intervention participants were given a tapering plan which was also sent to their GP and people were encouraged to make an appointment and discuss the plan. It was the GP who then actioned the plan if acceptable to both parties. The self-help booklet also suggested control participants could discuss tapering with their GPs. We have thus utilised quotations from intervention and control groups for this section on the role of the GP. There were five themes: (i) GP as information giver and sounding board, (ii) Trust in doctor, (iii) Teamwork, (iv) GP reluctance and (v) Access to GP. The table below gives exemplar quotes.

Table 5 Role of the GP

**Themes**	**Explanation**	***Exemplar quotes***
GP as information giver and sounding board.	Prior discussion with GPs helped participants to prepare. Often this meant more interest or confidence in IWOTCH.	*' Talking through [with the GP] made me realise I was ready' 4 control*
Trust in doctor	Participants spoke of a history of trying everything else before opioids and often upward titration. They accepted this as they trusted their doctor.	*I sort of trusted my doctor that he was doing the right thing by me… 28 control*
Teamwork	Some participants described a good working relationship with their doctor who supported and encouraged tapering.	*… I'd come down an awful lot and my doctor was absolutely gobsmacked… she calls me 'her star patient…' …she's a fantastic GP….. she wanted me to succeed as much as I did so it was teamwork but if you've got a good GP like that you're sailing aren't you but some are too busy! 31 intervention*
GP reluctance	There were some participants whose doctor was resistant to them tapering.	*'they didn't think I would get off them'[opioids] (because of their pain) 13 intervention*<br>*[You]Can't come off them we've tried all sorts 15 control*
Access to GP	Some participants felt it was difficult to get to see their GP	*… getting help really it's very difficult and getting an appointment up here is very difficult! 20 control*

For peer review only - http://bmjopen.bmj.com/site/about/guidelines.xhtml

Intervention Interviews – participants' experiences of the intervention (n=20)

We present 4 main themes with subthemes where appropriate.

1. *Acceptability of the intervention*
2. *Being in a Group*
   - *Shared experience*
   - *Social comparisons*
   - *Support*
   - *Being committed to something*
   - *Group numbers*
   - *Perceptions about group facilitators*
   - *Challenges to establishing group cohesion.*
3. *Group sessions of interest*
   - *Opioid Information*
   - *Distraction techniques*
   - *Anger irritability and frustration*
   - *Relaxation*
   - *Mindfulness*
4. *Nurse one to one consultations: participant perspectives*

Acceptability of the intervention

Half said that the intervention was delivered in an appropriate format. When asked about suggestions to improve it, key responses were; provision of more support especially as tapering progressed, more group time, less didactic delivery and more local venues. All agreed they would recommend the I-WOTCH approach to others although some noted that participants needed to be open and receptive.

Being in a Group

Looking back on the group intervention, most interviewees found that being in a group was beneficial. They spoke about the groups as a shared experience where they were with people in similar circumstances which included the lay facilitator with whom they could share experiences. There were social comparisons within the group with participants noticing that others were like, or not like, themselves. Social support from the group was a key theme with evidence of people encouraging others especially when they were experiencing difficulties. Interviewees often talked

For peer review only - http://bmjopen.bmj.com/site/about/guidelines.xhtml

about the commitment to the group and or the trial. Group numbers seemed important, less than three members limited discussion. Lay group facilitators were seen either as good examples of how people cope with chronic pain or, alternatively, that they were somehow not the same and may not have had the same long-term experience of opioids and pain. The former perception allowing greater identification with the lay facilitator. Not all groups ran smoothly especially if they included disruptive individuals (Figure 2).

Figure 2 Being in a group

Group session aspects of interest

There were five sessions which people talked about repeatedly across the data (see box 2) but on the whole people tended to talk globally about the intervention even when prompted to discuss individual sessions.

Box 2 Specific aspects spoken about repeatedly.

Aspects of Interest

Opioid Information:

Information sessions gave most participants new insights into opioids and their effects. Participants also learned about differences between dependence, tolerance and addiction and the effects of withdrawal and/or tapering and valued exploring these in the groups.

Distraction techniques:

Distraction techniques were felt to be the most useful technique, some recognised the technique in existing pastimes and hobbies.

Anger irritability and frustration:

This session was appreciated because participants felt they were not alone in having these feelings and that they had an opportunity to talk about them.

Relaxation:

Most liked the relaxation sessions but few used relaxation techniques regularly, as needed. Participants often confused relaxation with mindfulness.

Mindfulness:

Unless exposed to this previously, this was not well understood and often thought to be another form of relaxation. A few participants remained unsure about what it was.

For peer review only - http://bmjopen.bmj.com/site/about/guidelines.xhtml

Nurse one-to-one consultations: participant perspective

Most intervention participants talked about these sessions. A majority appreciated the tailoring of the tapering plan and how they could personally apply the elements they had learned from the intervention. Sessions were described as encouraging and supportive and almost all talked about having a good relationship with the nurse.

… she was hopeful she said I had the determination to succeed in coming off the Tramadol… erm… so I felt positive with that… that I had like sort of the support with the words that I could actually do it … … I just felt encouraged like that I could do it meself so I liked the support I suppose and the fact that they believed in me…yeah! 13

Participant feedback forms

Participants were given feedback forms at the end of day three in a sub-set of ten intervention groups, providing 31 responses (delays in submitting an amendment about this for ethical approval meant the earlier groups were not able to be asked to complete this feedback form). Overall, responses indicate positive appreciation of most aspects of the course, but this is a small sub-sample, reported in detail in supplementary material 9 and should be treated with caution.

Intervention delivery staff interviews

Staff Characteristics

We interviewed 18 intervention-delivery staff; nurse facilitators n=10, lay facilitators n=6 and other health care professional (HCP) facilitators n=2. Four (one nurse, 3 lay) did not respond. Identifier prefixes after each quotation are N for nurses, L for lay and H for healthcare professionals.

We have presented the four themes from the staff interviews: 1) Acceptability and training, 2) Running the group sessions (Venues, Facilitators and Group dynamics), 3) Factors affecting readiness to change across group sessions, and 4) One-to-one sessions.

For peer review only - http://bmjopen.bmj.com/site/about/guidelines.xhtml

Acceptability and training

Almost all were happy with the package as delivered. Quality assurance assessment on Day One was appreciated as was the general support from the team *"I wanted that there was somebody there on the first day actually because it was quite good to find out how… whether I was actually doing it [correctly]…"L05*

Staff interviewees regarded the training as adequate and the manual as comprehensive.

The manual "was very good because it took you through every single section very, very clearly." (N09)

"….it's quite thorough and really, really detailed. L03

Some would have liked more training on; practical facilitation and/or subjects with which they were not familiar e.g. mindfulness or teaching posture. Most put in extra unpaid time to read around relevant subjects and to practice their delivery, and most were happy doing this.

Running the sessions

- Venues varied greatly, particularly in community settings. There were instances when even working to a lone worker risk-assessment protocol, facilitators were asked to lock up, being last in the building. On a few occasions, telephone back up with the I-WOTCH team was compromised by a lack of signal. IT problems were mitigated by facilitators having written scripts as backup.
- Facilitators mostly worked well together, with only occasional disagreements over differences in delivery or choice of sessions. Most would have preferred to work in the same pairings which was not possible within the intervention delivery design. Nurses felt the days were quite 'full on' having little downtime because even in breaks and meal-breaks everybody still chatted. Lay facilitators who were having to deal with their own health issues commented that the travel and full days (09.00 to 15.00) left them tired. However, all the

For peer review only

facilitators said that the lay facilitator role was key to the groups running well. "… *do not remove the lay facilitators if you're going forward, they have to be there…" N02*

- Group dynamics

There was sometimes initial scepticism and negativity about the programme which was challenging for the staff to deal with. Resistance sometimes changed around day 2.

"… I think by sort of early afternoon is when people started you know listening a bit more thinking, 'Oh ok maybe there is an alternative?'" L07

Groups fared differently – occasionally groups bonded or 'gelled' as early as day one but more often by the end of day two and definitely by day three where shared experiences and supportive bonds had been formed.

"Now dealing with positive people looking and moving forward." L04

The most challenging groups involved a small minority of participants actively resistant to the information and techniques offered. Some showed challenging behaviour such as 'rubbishing' the content or interrupting. Some of these participants did not return after day 1.

'Shutters down' straight away. L05

Factors affecting readiness to change across group sessions – Categories of change

Intervention delivery staff described three presentations of change experience (across participants) and two turning points around the two main opioid information sessions. See boxes 3 and 4 below.

Box 3 Three categories of change experience identified by intervention delivery staff

Resistance to change
Some did not want to engage, either that they were sceptical about the information, had no side effects or were fearful of coming off opioids. This was sometimes due to bad experiences or felt reliant on them or wanted something else as a substitute.
… that was the bit they all dreaded… mmm… and they were super nervous and a lot of them I think weren't quite sure whether they wanted to take that step…L05

For peer review only - http://bmjopen.bmj.com/site/about/guidelines.xhtml

"some people seemed to have no intention of looking at other ways to deal with their pain. Not Ready. L04. People "start off pessimistic" L05

Open to Trying

Some participants were ready to try – motivated themselves or by the group or that they felt the medication perhaps wasn't working anyway. There was concern after learning more about opioids especially its effects, tolerance and dependence. Tapering slowly was a reassuring message rather than 'all or nothing' enabling participants to 'just give it a go.'

and sometimes they would talk themselves into it and talk themselves out of it… it was… cos it's a step into the unknown and we tell them this is what could possibly affect you …it did make it worse I… … cos some of the patients felt that they were already dealing with quite a bit you know to start with…… what made it really good was…… the other facilitator the nurse said you know 'it need not be a dramatic drop it can be a gentle tapering thing' and I think when they saw the tapering that settled them down at the end… L05

"Became friends…Reassurance that they are not alone" L07

Not the Right Time

Staff spoke about participants wanting to delay tapering until after an event: e.g. Holiday or surgery. They felt some were not able to manage it at that time but could reduce a little and try again later.

… there was only the one man at first on the first class who hadn't been on opioids long… didn't feel dependent on them yet but he said he'd learnt and he knew what to look out for so yes he would be you know aiming to come off them but not quite yet… L04

Box 4 *Turning points identified by intervention delivery staff*

1) on the morning of day one some were shocked and unaware of the issues and surprised to learn opioids aren't helpful long term.

Bit of an eye opener N09

I think they were surprised to learn that opioids aren't helpful long term… … they would've been I think of the view that… err… I need to take this because if I don't take it I'll be in even more pain rather than this doesn't seem to be working cos I'm still in pain after 20 years! N11

[Angry with Doctors] and I think they were quite cross that they were getting it and being giving it and the length of time they'd been given… mmm that didn't go down too well in any of the groups! L05

2) on the morning of day two when they started to think how they may start tapering and asked more questions. This was after the second session about opioids which covered withdrawal and John's tapering story which worked well.

…my opinion was that they really needed their hands holding if they were going to make the jump… H2

… and then on day two pretty much you can tell who is more open to it ……most of the time it was the majority of the group you'd have the one or two that were still sceptical during day two still kind of like asking us questions… L03

One-to-one sessions: nurse facilitator perspective

Nurse facilitators spoke of the tapering app being straightforward to use, although at times they were unable to get a mobile phone signal and occasionally the opioid the patient was taking was not included in the tapering app. Hard copies of the tapering plan were also written. On discussing the tapering plans suggested on the app algorithm, nurses and participants often wanted to taper more

For peer review only - http://bmjopen.bmj.com/site/about/guidelines.xhtml

slowly e.g., tramadol tablets decreasing by 50mgs but with no smaller dosages available, lack of confidence or having withdrawal effects. This was supported and nurses felt that it was important that the participant felt in control of the process.

> *..a lot of the participants … mmm.. didn't feel that they could taper at that speed and my sort of take on that is that it is a marathon not a sprint and you know if you can't taper at that speed then you know we'll go a bit further …'* N10

Nurses described participants wanting to talk about a lot of issues which they hadn't necessarily brought to the group and that they needed that opportunity to talk.

> *'it gives them an opportunity to talk openly with me and perhaps mention things that they haven't been able to mention within the group.'* N10

Some nurses spoke about seeing a difference in participants at their 1st and 2nd face to face.

> *..people used phrases like they'd felt they'd 'come from behind a curtain.' And they didn't seem quite so dazed…'N06*

Nurse data suggested that confidence, motivation, and the participant being in control were key factors to consider in the tapering process.

3) Implementation of the I-WOTCH group intervention

This section explores how the trial was implemented and the uptake of the components offered (figure 3).

Figure 3 Structure of the implementation items

For peer review only - http://bmjopen.bmj.com/site/about/guidelines.xhtml

Groups run (dose delivered)

The trial ran 35 groups - 20 in the midlands, 15 in the northeast, of England. At randomisation the mean group size was 9, SD 2.9, median group size 9 IQR 5 to 11

Uptake and attendance (dose received)

Minimal compliance is defined as attending at least Day 1 and the first one face to face consultation. There were 190 (62%) with at least minimal compliance. Full compliance is defined as attending at least Day 1, 2 & 3, the first one to one consultation and at least one phone call. There were 144 participants with full compliance (47%).

We randomised 305 people to the group intervention with 166 (54%) attending all three group days. The first one to one session attendance was 190 (62%) and 131 (43%) attended both the first and second session. 167 (55%) received one or more telephone calls. Thirteen (4%) did not return after day one (as mentioned in the staff interviews).

Non-attendance at the first and subsequent sessions was high at 90 (30%) (Full trial attendance see supplementary material 10).

Participant reasons for non-attendance throughout the study

Reasons for non-attendance (if given) were poor health, competing work commitments and family interests.

Fidelity

Fidelity score averages for the group sessions adherence was 83% - (very good 81-90% as rated by Borrelli et al 2011[14]), with a range of 25-100 and a median of 88 and overall competence 79% (good 71-80%), range 0-100, median 86 [14]. One to one session scores were high with an adherence average percentage score of 91%, range 61-100 and competence an average of 93%, range 50-100

For peer review only - http://bmjopen.bmj.com/site/about/guidelines.xhtml

(excellent 91-100% [14]) (supplementary material 11 and 12). The 25% adherence score was rated when there was a technical problem and the facilitator did not use the back-up scripts provided, so did not cover that section of the session as expected. The lack of facilitation skills denoted by a score of 0 on competence does suggest facilitation is a skill shared by most but not by all.

4) Potential Change Mechanisms

The control and intervention scores are comparable over the items of each question at baseline. The two baseline only questions (2 and 3) (supplementary material 6) were; Baseline expectation: *I expect that, in 4 months' time, I will have reduced my opioid use* and Baseline self-efficacy *I am confident I could reduce my opioid use a lot over 4 months which* showed a trend of low expectations of successful tapering within 4 months and low self-efficacy/confidence in tapering (supplementary material 13).

The motivation to reduce opioids question showed a trend for more participants wanting to cut down or stop their opioids at baseline. There is a weak trend showing the self-management group to be more slightly more motivated to continue tapering at later timepoints. However, this should be viewed with caution due to the high proportion of missing data for this item.

Our statistician ran statistical analyses on the full data collected; baseline four, eight and 12 months to look for any impact of responses to the change mechanism questions on main trial outcomes (see supplementary material 14). There were few significant correlations except for the study perceived credibility question. Correlations indicate that more participants attributed their tapering success to involvement in the intervention arm of I-WOTCH with the reverse trend in the control group. (supplementary material 15).

For peer review only - http://bmjopen.bmj.com/site/about/guidelines.xhtml

5) Contextual issues

The main contextual issues were trying to live a life, often with multiple health problems and medications alongside managing multiple appointments, tests and operations. Day to day life is often a challenge dealing with their pain and the things they need to get done. Living with chronic pain can be challenging physically, mentally and emotionally. For some just the idea of starting to taper is 'a step too far' that they may be unwilling or unable to consider. This was seen in the participant interviews and the staff interviews especially with the three categories of change given previously in table 9 when people are; 'Resistant to change', 'Open to Trying' or that it's 'Not the Right Time.'

Overarching Themes from integration of qualitative and quantitative data

Analyses revealed threads across the qualitative and quantitative data which generated four main overarching themes: (1) the right time, (2) the backdrop of life with chronic pain, (3) needing support and (4) the group effect.

1. The right time. This theme encompasses evidence indicating whether someone is ready to change, including enablers or barriers to tapering and whether participants felt fully informed, motivated, and confident to be able to taper.
2. Life with chronic pain is often complex with many having multiple health problems and medications. This means that participants were living life around their pain, pain relief, healthcare appointments and procedures as well as social and family commitments. Each of these could affect their decision about when or whether to taper.
3. Support is needed at all phases of tapering. This could come from their family, GP or from a tapering intervention such as I-WOTCH which agreed a tailored tapering programme. If

For peer review only - http://bmjopen.bmj.com/site/about/guidelines.xhtml

family members or their GPs were ambivalent or against tapering, people were less keen to try it.

4. The group effect allowed for people taking opioid medication to come together to gain more information and skills in pain management as well as choosing whether to start on a tapering path as a joint group goal. They were given the opportunity to discuss and explore their fears and motivations about decreasing their medication.

The interaction between the overarching themes can be seen in our model. See figure 4. Participants make decisions about tapering based upon whether it is the right time for them, their current pain experiences and healthcare and the support available to them. Helping participants to identify and discuss potential barriers whilst fostering knowledge, motivation, confidence and self-management skills means that tapering is more likely to take place.

Figure 4 Conceptual model of overarching themes

Discussion

The I-WOTCH main trial results showed that 62/225 [29%] of participants in the intervention group stopped taking opioids, compared to 15/208 [7%] in the usual care group at 12 month follow-up without any difference in pain, or pain related disability, between the study arms. The process evaluation data indicates that the intervention was delivered as intended with good fidelity scores. There was evidence of participants attributing their opioid reduction to the I-WOTCH study. Participants and staff alike felt the package as whole was acceptable and deliverable. However poor attendance was an issue with 90 participants randomised to the intervention not attending any group.

The secret ingredient?

No one factor or 'secret ingredient' was identified but rather a combination of active ingredients as enablers of opioid tapering. The I-WOTCH intervention seemed to have a synergistic, summative effect from its different components which could be specific to the individual. The qualitative data

For peer review only - http://bmjopen.bmj.com/site/about/guidelines.xhtml

indicate that the intervention served to inform and provide a forum where discussion in groups and in one-to-one sessions could help people decide whether to reduce their opioid use. The final decision was the participant's, and no judgements were made about whether or when they might start tapering. The I-WOTCH intervention provided a space in which people could fill gaps in their knowledge of opioids. The intervention also enhanced motivations to taper, including intrinsic motivations that participants brought to the sessions. Information provided by the intervention about the lack of long-term effectiveness and side effects developed motivation. This was emphasised in staff interviews which highlighted how provision of information about opioid use consequences was, for some participants, a turning point in becoming motivated to change. Support from their GP, their family, the I-WOTCH one-to-one meetings, the tapering plan and group peers also enhanced motivation. Exploring pain management skills in a group over three sessions allowed them to try things out and swap ideas, so establishing the skill base needed for change.

The Information, Motivation and Behavioural Skills model[8,9] provided the theoretical foundation and guided our understanding of key components of change that would support action towards opioid tapering. The qualitative data confirm the utility of identifying sources of information, developing motivation at the right time and skill development, including use of the lay facilitator as a role model, as critical precursors of change. This process evaluation reveals how the I-WOTCH intervention provided all three of these critical supports. Barriers to change may be specific to the individual but supporting people to become informed and to develop motivation and skills optimises the chances of behaviour change. The main barriers were that it might not have been the right time for participants to consider tapering and other things in life often took priority. Fears of returning to uncontrolled pain were common alongside multiple other potential barriers.

These findings correspond to a wider literature. A 2020 qualitative evidence synthesis using meta-ethnography (31 studies) looking at experiences of people taking opioids for chronic non-malignant

For peer review only - http://bmjopen.bmj.com/site/about/guidelines.xhtml

pain by the current authors [15], found five themes, four of which resonated with our findings. The fifth theme was around societal stigma, which did not emerge from the data in this English study but was common in North American studies, where law enforcement agencies can be involved [16]. Our findings are also broadly supported by recent literature. Goesling et al (2019) who held focus groups with former opioid users about their experiences before, during and after opioid cessation [17]. Motivators to stop included concerns about a lack of opioid efficacy, addiction, and quality of life impact. Quinlan et al (2021) noted that 60 patients undertaking a pre tapering survey found there were more barriers given than motivators [18]. The main areas of concern were around quality of life, pain and withdrawal which the authors suggest need to be addressed for successful tapering to take place. Henry et al (2019) conducted focus groups and interviews with 21 adults with chronic pain at different stages of opioid tapering [19]. They explored the complex contextual backdrop of life with chronic pain describing changes or fluctuations in many areas including pain, social relationships, health status, emotional state and the perceived need for opioids at any given time. They recognise the substantial effort involved for people undertaking the tapering journey, the effect on their day to day lives and the strategies used. They suggest early anticipatory guidance about tapering and that tapering should be patient centred and responsive to the patients' needs.

We identified GPs as integral to tapering support. Three 2020-22 papers from health care providers perspective[20-22] throw light on the complex challenges involved in tapering opioids, especially the importance of maintaining a good patient /provider relationship. Hamilton et al 2021 also cite patient motivation to be a central factor for successful tapering [22]. GPs are seen as authoritative experts in relation to health behaviour and patients often welcome behaviour change advice from their GPs, especially when such advice may have a positive effect on long-term condition management [23]. There is a long history of successful use of small groups to develop support, share common health problems, build trust, share ideas, enhance information, change attitudes, and develop motivation and

For peer review only - http://bmjopen.bmj.com/site/about/guidelines.xhtml

skills to promote health behaviour change [24-25]. This evaluation shows that the I-WOTCH intervention successfully applied these group processes in supporting individual change. Participants often had a strong sense of shared experience around opioid use and the challenges of use reduction. They felt as if they were all in the same boat. This is a key foundation for successful group interventions[26]. There is also evidence of participants experiencing social support and social learning both key to individual change. Facilitation of interaction in small groups is critical to optimising change and interviews revealed that the lay facilitator role was very important to participants and nursing facilitators alike. Facilitators did not underestimate the challenges some participants might face and appreciated the lay facilitator's personal experience with which participants could identity and then learn from. The challenges of group facilitation observed also emphasise the importance of careful skill-based training for group facilitators [25]. At least minimum compliance was achieved with 62% of participants, and full compliance with 47%. All three group sessions were attended by 54%. We note that even with this level of compliance, clinically important differences were found. Even though adherence was less than we had hoped we have got a very clear positive result on one of the trial primary outcomes. It seems likely shared decision making between patients and GPs could be important to increasing compliance, although this would need to be evaluated. For future delivery, this study demonstrated that group sessions were an important part of the intervention, The one intervention that had the least positive feedback was mindfulness. However, we are reluctant to suggest this could be removed as not all facilitators felt comfortable explaining this element of the programme.

Conclusion

Our conceptual model shows the active ingredients involved in an ideal pathway to successful tapering. This may look straightforward at first glance; however, the reality is that there are multiple

For peer review only - http://bmjopen.bmj.com/site/about/guidelines.xhtml

contextual issues and barriers which influence someone's ability to taper. The I-WOTCH intervention gave practical strategies, information, support, room for discussion and a tailored tapering plan to help participants navigate this difficult journey.

Acknowledgements

We would like to thank the participants and intervention delivery staff who were interviewed for their valuable input into the process evaluation. Also the members of the I-WOTCH team.
Alleyne, Sharisse
Balasubramanian, Shyam
Betteley, Lauren
Booth, Katie
Carnes, Dawn
Furlan, Andrea D
Haywood, Kirstie
Iglesias Urrutia, Cynthia Paola
Lall, Ranjit
Manca, Andrea
Mistry, Dipesh
Noyes, Jennifer
Rahman, Anisur
Shaw, Jane
Tang, Nicole K Y
Taylor, Stephanie
Tysall, Colin
Underwood, Martin
Withers, Emma J

Collaborators

Author contribution:
Conceived the original study design: KS, MU, SE and HKS
Developed the study design and plan for data collection and undertook analysis. VPN, KS and CA
All authors have provided critical revisions to the manuscript and approved the final manuscript.

Funding

This project is funded by the National Institute for Health Research (NIHR), Heath Technology Assessment (HTA) (project number 14/224/04. The views and opinions expressed therein are those of the authors and do not necessarily reflect those of the HTA, NIHR, NHS or the Department of Health.

Competing interests

MU is chief investigator or co-investigator on multiple previous and current research grants from the UK National Institute for Health Research, and Arthritis Research UK and is a co-investigator on

For peer review only

grants funded by the Australian NHMRC and Norwegian MRC. He was an NIHR Senior Investigator until March 2021. He is a director and shareholder of Clinvivo Ltd that provides electronic data collection for health services research. He receives some salary support from University Hospitals Coventry and Warwickshire He is a co-investigator on two current and one completed NIHR funded studies that are, of have had, additional support from Stryker Ltd. Until March 2020 he was an editor of the NIHR journal series, and a member of the NIHR Journal Editors Group, for which he received a fee.

KS is chief or co-investigator on many previous and current grants from the UK National Institute for Health Research. She also sat on a NIHR funding board (HS&DR) until 2018.

SE is a clinician and Chief investigator on several past and ongoing trials, his department has received research support from the NIHR as well as Medtronic, Boston Scientific and Saluda Medical. He is a paid consultant to Medtronic, Saluda Medical and Mainstay Medical.

Data Sharing: Data available upon reasonable request.

Ethical approval given by Yorkshire and the Humber-South Yorkshire Research Ethics Committee on 13 Sept 2016 (16/YH/0325)

References

1. Moore GF, Audrey S, Barker M, et al. Process evaluation of complex interventions: Medical Research Council guidance. 2015;350.
2. Skivington K, Matthews L, Simpson SA, et al. A new framework for developing and evaluating complex interventions: update of Medical Research Council guidance. *BMJ*2021;374:n2061.
3. Nichols VP, Abraham C, Eldabe S, et al. Process evaluation protocol for the I-WOTCH study: an opioid tapering support programme for people with chronic non-malignant pain. *BMJ Open*2019;9:e028998.
4. Sandhu HK, Shaw J, Carnes D, et al. Development and testing of an opioid tapering self-management intervention for chronic pain: I-WOTCH. *BMJ Open*2022;12:e053725.
5. Sandhu HK, Abraham C, Alleyne S, et al. Testing a support programme for opioid reduction for people with chronic non-malignant pain: the I-WOTCH randomised controlled trial protocol. *BMJ Open*2019;9:e028937.
6. Sandhu HK, Booth K, Furlan AD et al Reducing Opioid Use for Chronic Pain With a Group-Based Intervention: A Randomized Clinical Trial. JAMA. 2023 May 23;329(20):1745-1756. doi: 10.1001/jama.2023.6454. PMID: 37219554; PMCID: PMC10208139.
7. Linnan L and Steckler A. Process evaluation for public health interventions and research San Francisco: Jossey-Bass, 2002.
8. Fisher JD, Fisher WA. Changing AIDS risk behavior. Psychological Bulletin. 1992;111:455–474.
9. Fisher JD, Fisher WA. & Harman, J. (2003). The Information Motivation–Behavioral Skills Model: A general social psychological. In, Social Psychological Foundations of Health and Illness (Eds. Suls, J & Wallston, K A), Blackwell Publishing Ltd. Oxford.
10. Ritchie J and Spencer L. In: The qualitative researcher's companion. In: Huberman AM, & Miles, M. B. (Eds.), (ed.). *Qualitative data analysis for applied policy research.*: SAGE Publications, Inc 2002: p. 305–29.

For peer review only - http://bmjopen.bmj.com/site/about/guidelines.xhtml

11. Braun V and Clarke VJQRP. Using thematic analysis in psychology. 2006;3.
12. O'Cathain A, Murphy E and Nicholl J. Three techniques for integrating data in mixed methods studies. *BMJ*2010;341:c4587.
13. GOV.UK. Data Protection Act. In: Collections, (ed.). 2018. https://www.legislation.gov.uk/ukpga/2018/12/contents/enacted. Accessed 4-4-23
14. Borrelli B, Sepinwall D, Ernst D, et al. A new tool to assess treatment fidelity and evaluation of treatment fidelity across 10 years of health behavior research. 2005;73.
15. Nichols VP, Toye F, Eldabe S, et al. Experiences of people taking opioid medication for chronic non-malignant pain: a qualitative evidence synthesis using meta-ethnography. *BMJ Open*2020;10:e032988.
16. Paulozzi L and Baldwin G. CDC grand rounds: prescription drug overdoses - a U.S. epidemic. *MMWR Morb Mortal Wkly Rep*2012;61:10-3.
17. Goesling J, DeJonckheere M, Pierce J, et al. Opioid cessation and chronic pain: perspectives of former opioid users. *Pain*2019;160:1131-45.
18. Quinlan J, Willson H and Grange K. Hopes and fears before opioid tapering: a quantitative and qualitative study of patients with chronic pain and long-term opioids. *British Journal of Pain*2021;15:120-28.
19. Henry SG, Paterniti DA, Feng B, et al. Patients' Experience With Opioid Tapering: A Conceptual Model With Recommendations for Clinicians. *J Pain*2019;20:181-91.
20. Langford AV, Gnjidic D, Lin CC, et al. "The lesser of two evils": a framework analysis of consumers' perspectives on opioid deprescribing and the development of opioid deprescribing guidelines. *Pain*2021;162:2686-92.
21. Matthias MS, Talib TL and Huffman MA. Managing Chronic Pain in an Opioid Crisis: What Is the Role of Shared Decision-Making? *Health Commun*2020;35:1239-47.
22. Hamilton M, Mathieson S, Gnjidic D, et al. Barriers, facilitators, and resources to opioid deprescribing in primary care: experiences of general practitioners in Australia. *Pain*2022;163:e518-e26.
23. Keyworth C, Epton T, Goldthorpe J, et al. Perceptions of receiving behaviour change interventions from GPs during routine consultations: A qualitative study. *PLoS One*2020;15:e0233399.
24. Borek AJ and Abraham C. How do small groups promote behaviour change? An integrative conceptual review of explanatory mechanisms. *Applied Psychology: Health and Well-Being*2018;10:30-61.
25. Borek AJ, Abraham C, Greaves CJ, et al. Identifying change processes in group-based health behaviour-change interventions: development of the mechanisms of action in group-based interventions (MAGI) framework. *Health Psychol Rev*2019:1-21.
26. Borek AJ, Abraham C, Greaves CJ, et al. 'We're all in the same boat': A qualitative study on how groups work in a diabetes prevention and management programme. *Br J Health Psychol*2019;24:787-805.

For peer review only - http://bmjopen.bmj.com/site/about/guidelines.xhtml

For peer review only

Figure 1 – Qualitative data sources

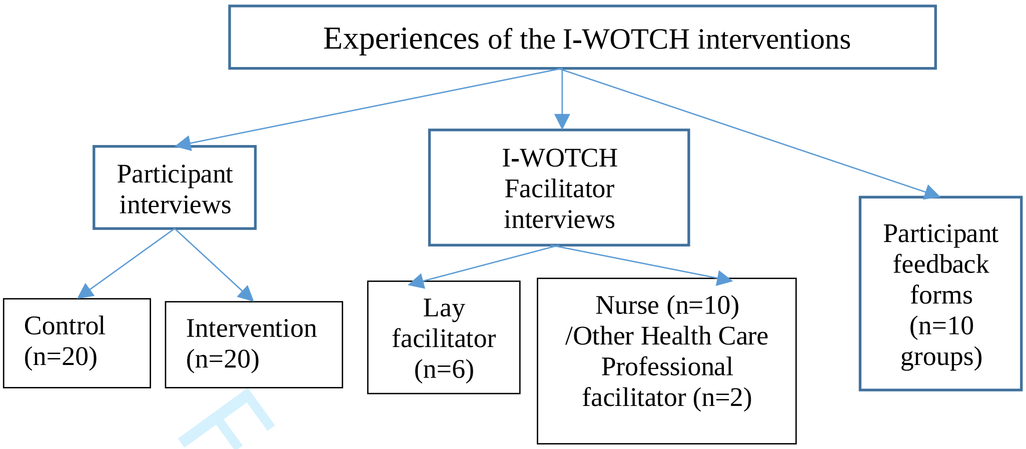

For peer review only

For peer review only

Figure 2 Being in a Group

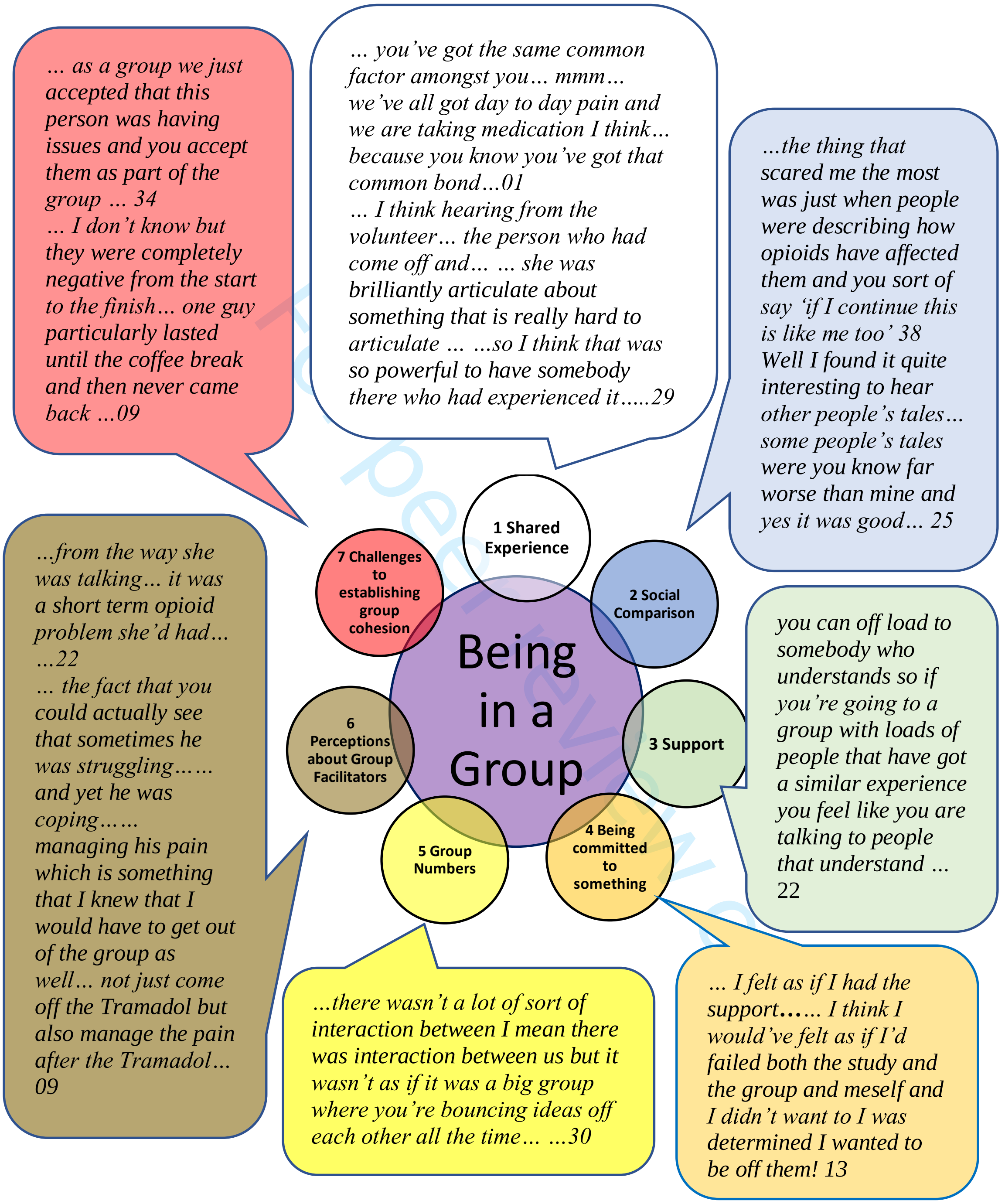

For peer review only

Figure 3 – Structure of the implementation items

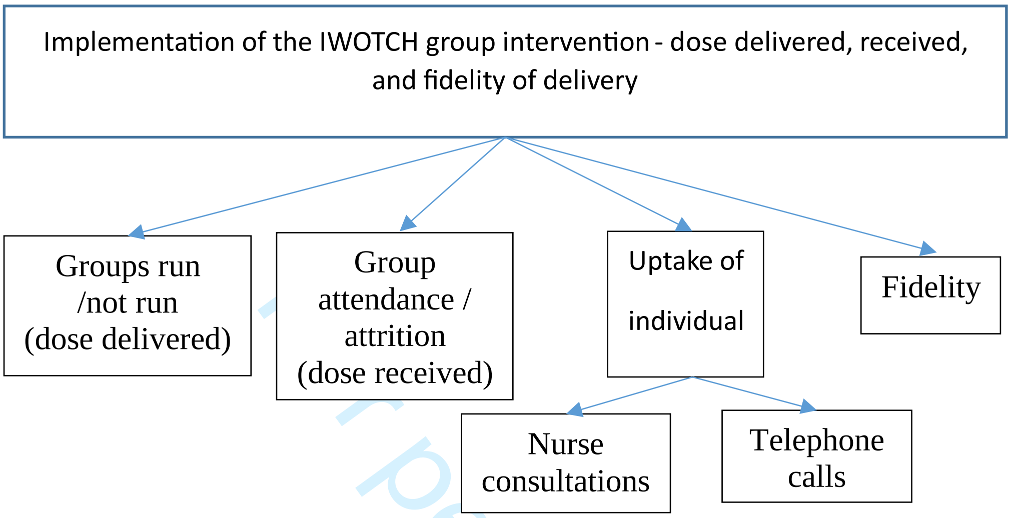

Figure 4 – Conceptual model of overarching themes

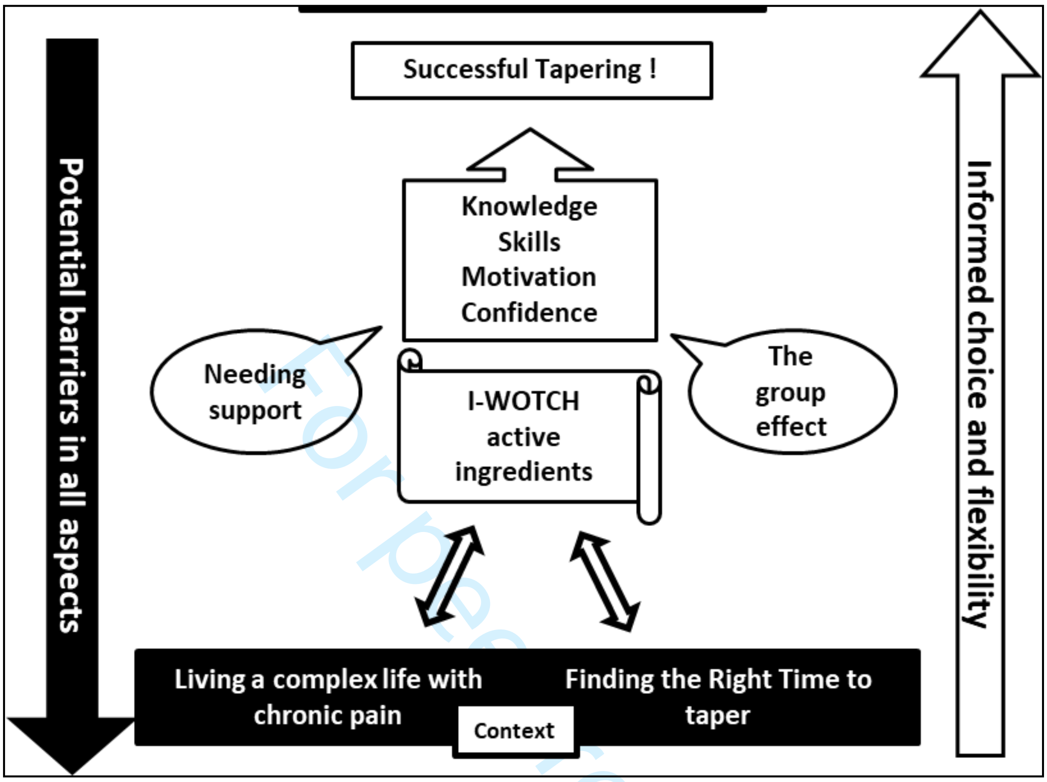

I-WOTCH Process evaluation supplementary material 1-10

Supplementary material 1 Logic Model

The problem	Intervention Aims	Intervention	Theory and Guidance	Interim Targets	Desired Outcomes
People with chronic non-malignant pain are taking opioids, which have side effects and are not effective in the long term.	To test the effectiveness and cost effectiveness of a patient-centred multicomponent self-management intervention targeting withdrawal of strong opioids on activities of daily living for people living with chronic non-malignant pain	**Manualised Intervention Delivery**<br>**Core pain management topics:**<br>• Acute versus Chronic pain<br>• Acceptance<br>• Attention Control and distraction<br>• the pain cycle<br>• Posture and movement advice<br>• Relaxation techniques<br>• Stress busting for health action planning, problem solving, pacing, SMART goal setting<br>• identifying and overcoming barriers to change<br>• Mindfulness<br>• Anger, irritability and frustration<br>• Communication Skills<br><br>**Core opioid specific topics:**<br>• The rationale of prescribing in chronic pain<br>• Opioid induced tolerance and need for dose escalation<br>• Evidence of usefulness of opioids short and long term<br>• Side effects of opioids short term and long term<br>• Case studies of successful discontinued opioid therapy<br>• Opioid withdrawal symptoms	Theory of Planned Behaviour<br><br>Social Cognitive Theory<br><br>Information Motivation and Behavioural (IMB model) skills<br><br>Patient Centred Communication<br><br>Motivational Interviewing	**Staff Training**<br>To facilitate groups, deliver individual tapering consultations and telephone support in an inclusive and non-judgemental manner<br>**Individual participant changes:**<br>a Knowledge of: opioids, withdrawal effects, chronic pain<br>b Fostering change: self-validation, legitimising pain, normalising expectations<br>c Motivation to change by: Improved self-efficacy, effective tapering<br>d Skills:<br>• General Self-Regulation<br>*Psychological skills*<br>Identify reasons for negative emotions (anger /frustration /irritable)<br>Identify problems and solutions, barriers to change<br>Recognise errors in thinking/automatic thoughts<br>Goal setting, goal review<br>*Physical skills*<br>Promote body awareness, posture<br>Reduce muscle tension<br>Body awareness and core strength<br>Relaxation-contract relax<br>• Pain Self-Regulation<br>Understand that pain and mood are linked – when is pain bearable and when not bearable.<br>Understanding of pain cycle, unhelpful emotions and behaviours<br>Using mind to relieve pain does not mean pain in mind<br>Distraction whilst relaxed<br>Focus mind away from pain	**Primary outcomes:**<br>Patient-Reported Outcomes Measurement Information System (PROMIS) Pain Interference Short Form (8A)(PROMIS -PI-SF-8A)<br><br>Daily morphine equivalent opioid dose

For peer review only

		• Advantages of slow supervised tapering<br>• Symptom management during tapering<br>• Pain control after opioids		Mindfulness for pain<br>Managing flare ups<br>Need for stretching<br>• <u>Communication Skills</u><br>How to communicate with General Practitioners (GPs) and Health Care Professionals (HCPs)<br>Listening skills - Active and giving feedback in communication-reward for help.	

For peer review only

Supplementary material 2 I-WOTCH Indicative Interview Topic Guide

Patient Participants

What did you hope to achieve by participating in this research?

Do you want to reduce your opioid intake- and if so why?

How important is it to you to reduce your opioid intake (that is, how strongly motivated are you)?

What were your expectations about whether this would work for you once you heard which group you would be in? (Intervention [I-WOTCH]) /best usual care [my opioid manager plus relaxation package])

How did your expectations match your actual experience?

How did you find using I-WOTCH/my opioid manager/relaxation package? (Where any parts easy for you? Were any parts difficult for you? Why was this?)

Did you find you were you able to use all the different components or not? (Prompt list of the components of intervention/control). If not, which components did you use? Why was this? Were there components you rarely or never used? Why was this?

What was it like trying to use this in your everyday life? (barriers/enablers)

Intervention group only: I'd like to ask you about your experiences of being in a group – how was that for you? (What was good about it, what was less good about it?)

Following from above…. How did the group work, was it better than having one-to-one sessions? Why?

How well was the group facilitated?

If you could change three things about the intervention what would those be?

If another patient asked you about taking part in this programme, what would you say?

Is there anything else you'd like to say that is important to you that I haven't asked you?

For peer review only

Supplementary material 3 Random sampling chart

	Group Number																													
	1	2	3	4	5	6	7	8	9	10	11	12	13	14	15	16	17	18	19	20	21	22	23	24	25	26	27	28	29	30
Sample 1		2		1		3					2			1			3				2		1	3						

All of the intervention groups were given a chronological number. To identify which groups would be checked for fidelity, for each block of ten groups (representing early, mid and later stages of the study) the trial statistician randomly allocated a day 1, day 2 and day 3 session.

For peer review only

Supplementary material 4 Fidelity data collection checklist (sessions in bold assessed for fidelity)

	*Educational and/or self-management regarding pain or opioid use	Practical, reflection or summarising sessions (not assessed for fidelity, recorded if took place or not)
Day 1	**2, 3, 4, 7, 8**	1,5,6,9,10,11
Day 2	**13, 14, 16**	12,15,17,18,19
Day 3	**21, 22, 23**	20,22 part 2, 24,25,26,27,28

**DAY 1**
Session 1 Introduction
***Session 2 Pain information**
***Session 3 Painkiller information and opioid education**
***Session 4 Acceptance: John's story**
Session 5 Attention Control and distraction
Session 6 Distraction activity – rose drawing
***Session 7 Good days, Bad days when is pain bearable and when is it not?**
***Session 8 The pain cycle unhelpful emotions and behaviours**
Session 9 Posture
Session 10 Relaxation and Breathing
Session 11 Summary of the day

**DAY 2**
Session 12 Reflections from day 1
***Session 13 Stress-busting – prioritising what's important, action planning, goal setting and pacing**
***Session 14 Withdrawal symptoms, case studies (Opioid Education 2)**
Session 15 Distraction activity- origami
***Session 16 Identifying and overcoming Barriers to change Part 1 – recognising unhelpful thinking**
***Identifying and overcoming Barriers to change Part 2– reframing negatives to positives**
Session 17 Mindful attention control
Session 18 Balance and introduction to stretch
Session 19 Summary of the Day

**DAY 3**
Session 20 Reflections from day 2 and previous week
***Session 21 Anger, irritability and frustration**
***Session 22 Relationships Part 1 Getting the most from your healthcare team**
Session 22 Part 2 Relationships Part 2 Listening skills
***Session 23 Managing setbacks and non-drug management techniques**
Session 24 Distraction activity – mindfulness colouring
Session 25 Stretching muscles that commonly get tight
Session 26 Mindfulness of thoughts and Senses
Session 27 Summary of Day 3
Session 28 Summary of the course

For peer review only

Supplementary material 5 Examples of I-WOTCH fidelity sessional and nurse consultation score sheets for adherence and competence

Day 1 /Session 2 /Title: Pain Information 30mins

Adherence: of the delivery as per protocol

Instructions: When at all possible please rate as 'Yes' or 'No' If 'partially' then write reason in comments box. Questions need not be verbatim (unless specified) as long as content of session is covered.

No.	Item	Adherence	Comments
Intro	Did the facilitator(s) introduce the session?	Yes (2) Partially (1) No (0)	
Step 1	Did the facilitator(s) play the DVD of the biomedical explanation about acute and chronic pain?	Yes (2) Partially (1) No (0)	
	Did the facilitator(s) ask the group Q1 and discuss, "What do you think about this explanation of pain? Is it missing anything?"	Yes (2) Partially (1) No (0)	
Step 2	Did the facilitator(s) present the bio-psycho-social explanation of pain?	Yes (2) Partially (1) No (0)	
	Did the facilitator(s) ask the group Q2 and discuss, "What do you think about this explanation of pain?"	Yes (2) Partially (1) No (0)	
Step 3	Did the facilitator(s) play the DVD of Experiences of living with opioid- treated long term pain?	Yes (2) Partially (1) No (0)	
	Did the facilitator(s) ask the group Q3 and discuss, "What do you think about Caroline's description of living with opioid-treated long-term pain?"	Yes (2) Partially (1) No (0)	
Summary	Did the facilitator(s) consolidate/embed the group's learning at the end of the session? *e.g. reading the summary, putting the session in context*	Yes (2) Partially (1) No (0)	
	Total adherence score (max 16)		
	Percentage adherence score (Total adherence score */16x100)		

Comments: For use if sessions; go off track, include items which are not on checklist, contain surprising unforeseen aspects or the facilitation wasn't covered as intended. Also if there was no opportunity to demonstrate the skill listed.

For peer review only

Day 1 /Session 2 /Title: Pain Information and Opioid Education

Competence: **of the quality of delivery or 'skill' of the facilitators**

	Item	Competence measure	Comments (*use box below to expand)*
1	Did the facilitator(s) create opportunities for discussion *e.g. did they; encourage individuals to participate, ask open questions, give enough time for the group to answer (rather than answer their own questions)*	Evident (2)<br>Partially evident (1)<br>Not evident (0)<br>Did not happen in this session (N/A)	
2	Did the facilitator(s) encourage individual disclosure? *e.g. did they ask different group members to comment or encourage the group to explore issues further (either individually or as a group)?*	Evident (2)<br>Partially evident (1)<br>Not evident (0)<br>Did not happen in this session (N/A)	
3	Did the facilitator(s) validate participants' disclosures? *e.g. Do other people find this/think that? I know how you feel. Sometimes people may feel differently about things.*	Evident (2)<br>Partially evident (1)<br>Not evident (0)<br>Did not happen in this session (N/A)	
4	Did the facilitator(s) give encouraging feedback on participants reported behaviours? *e.g. Did they give appraisal 'that's really good' or 'that's really good but I wonder if…'*	Evident (2)<br>Partially evident (1)<br>Not evident (0)<br>Did not happen in this session (N/A)	
5	Did the facilitator(s) foster a positive group climate? *e.g. did they; use humour, say positive things about people 'that's a helpful comment' 'thank you for sharing that'*	Evident (2)<br>Partially evident (1)<br>Not evident (0)<br>Did not happen in this session (N/A)	
6	Did the facilitator acknowledge and respond appropriately to admissions or statements of low self-efficacy? e.g. *'yes this can be difficult but…' ideas or examples offered of how this may be done. Issues surrounding confidence.*	Evident (2)<br>Partially evident (1)<br>Not evident (0)<br>Did not happen in this session (N/A)	
7	Did the facilitator respond appropriately to disclosures of negative events or barriers to progress?	Evident (2)<br>Partially evident (1)<br>Not evident (0)<br>Did not happen in this session (N/A)	
	Total competence score (max 14)		
	Percentage competence score		

Comments: For use if sessions; go off track, include items which are not on checklist, contain surprising unforeseen aspects or the facilitation wasn't covered as intended. Also if there was no opportunity to demonstrate the skill listed.

For peer review only

Fidelity score sheet for first one to one nurse consultation

Item	Adherence	Score	Comments *(expand in box below)*
1	Was the participant asked about their thoughts/feelings on reducing their opioids?	Yes (2)<br>Partially (1)<br>No (0)	
2	Was the participant asked about their pain related medication usage?	Yes (2)<br>Partially (1)<br>No (0)	
3	Was the participant's pain discussed?	Yes (2)<br>Partially (1)<br>No (0)	
4	Was a tapering plan discussed/negotiated?	Yes (2)<br>Partially (1)<br>No (0)<br>Not evident	
5	Were temporary withdrawal side effects mentioned?	Yes (2)<br>Partially (1)<br>No (0)	
6	Was a tapering plan summarised and a copy given to the participant?	Yes(2)<br>Partially (1)<br>No (0)	
7	Were barriers to implementing the tapering plan and setbacks discussed?	Yes (2)<br>Partially (1)<br>No (0)<br>Not evident	
8	Did the nurse leave an opportunity for any questions?	Yes (2)<br>Partially (1)<br>No (0)	
9	Did the nurse ensure the participant knew to make an appointment with the GP?	Yes (2)<br>Partially (1)<br>No (0)	
	Total score out of 14 or 18 max		
	Percentage score total score/ 14 or 18 x 100		
Comments:			

Item	Competence	Score	Comments *(expand in box below if nec.)*

For peer review only

1	Did the nurse allow participants to express their concerns/achievements?	Evident (2)<br>Partially evident (1)<br>Not evident (0)	
2	Did the nurse allow participants to discuss and/or explore their concerns/achievements?	Evident (2)<br>Partially evident (1)<br>Not evident (0)	
3	Did the nurse demonstrate empathy? e.g. *Did they show they understood the participant's feelings?*	Evident (2)<br>Partially evident (1)<br>Not evident (0)	
4	Did the nurse accept the participant's perspective? e.*g. Did they allow exploration of positive and negative feelings about pain and/or opioids, were they non-judgmental?*	Evident (2)<br>Partially evident (1)<br>Not evident (0)	
5	Did the nurse actively listen to the participant? e.g. *Did they use 'uhuu', 'oh' 'um' 'really' type of phrases to demonstrate they were listening*	Evident (2)<br>Partially evident (1)<br>Not evident (0)	
6	Did they support self-efficacy? *e.g. Did they offer reassurance, suggest other techniques, congratulate them on any successes or small steps in the right direction.*	Evident (2)<br>Partially evident (1)<br>Not evident (0)	
	Total score out of 12 max		
	Percentage score total score/ 12 x 100		
Comments:			

For peer review only

Supplementary material 6 Change Mechanism Questions

Motivation, expectation, self-efficacy and perceived intervention efficacy questions

Baseline motivation (baseline and follow-up).

I want to reduce my opioid use.

(Not at all, by a little, by half, so I only use a little, so I use no opioids)

Baseline expectation (baseline only).

I expect that, in 4 months' time, I will have reduced my opioid use.

(Not at all, by a little, by half, so I only use a little, so I use no opioids)

Baseline self-efficacy (baseline only).

I am confident I could reduce my opioid use a lot over 4 months.

(Not at all confident, somewhat confident, fairly confident, strongly confident, completely confident)

Perceived intervention efficacy (baseline and follow-up).

Baseline

I feel that involvement in this study can help me to reduce my opioid use.

(Not at all, by a little, by half, so I only use a little, so I use no opioids)

Follow-up

I feel that involvement in this study has helped me to reduce my opioid use.

(Not at all, by a little, by half, so I only use a little, so I use no opioids)

Supplementary material 7 Interviewee characteristics and uptake for the interview study

Interviewee ID	Location cohort ID NE (Northeast) orblank(midlands)	Gender	Age decade	Opioid usage at 12m compared to baseline*	Opioid Type	Baseline Morphine Equivalent (ME) **	Allocation***
Pilot 1	P1	F	70's	0	Di hydrocodeine / morphine	30-59	Int
3	1	F	30's	S	Tramadol MR	0-29	Intention to treat
4	1	F	50's	L by half	Tramadol	0-29	UC
5	2	M	60's	0	Buprenorphine patches	60-89	Int
6	3	M	60's	H	Tramadol and cocodamol	0-29	UC
7	3	F	50's	L sl	Buprenorphine patches	30-59	Int
8	5 NE	F	60's	0	Zomorph(morphine)	30-59	UC
9	5 NE	M	60's	0	Tramadol	0-29	Int
10	4 NE	F	50's	0	Fentanyl patches	30-59	Int
11	6	F	40's	S	Morphine SR and Oromorph	150+	UC
12	7 NE	F	50's	L	Oxycodone and liquid morphine	90-119	UC
13	12 NE	F	50's	0	Tramadol	0-29	Int
14	9 NE	M	60's	S	Tramadol	0-29	Int
15	11	F	70's	H *(?)*	Tramadol	0-29	UC
16	11	M	60's	H	Oxycodone	30-59	Int
17	10	M	70's	S	Tramadol	0-29	UC
18	8	F	80's	0	Morphine	0-29	UC
19	17 NE	M	60's	L sl.	Tapentadol /morphine/ liquid morphine	60-89	UC
20	9 NE	M	60's	L	Morphine / Codeine phosphate / Tramadol	60-89	UC
21	15	F	60's	L sl	Morphine / liquid morphine	150+	Int
22	8	M	50's	H	Tramadol / Morphine	60-89	Int

For peer review only

23	13	M	70's	H	Liquid morphine and Codeine phosphate	0-29	UC
24	16	M	40/*50*	(S) Still on	Oxycodone changed to fentanyl unknown 12m ME	90-119	UC
25	16	F	80	?L	Buprenorphine patches	30-59	Int
26	13	M	70	L	Morphine + ?	60-89	Int
27	19	F	50's	H	Fentanyl patches	150+	UC
28	18 NE	F	70s	H	Morphine + liquid morphine	150+	UC
29	17 NE	F	50/60	L	Codeine phosphate and fentanyl patches	150+	Int
30	22 NE	M	60/70	L	Tramadol	0-29	Int
31	20	F	60's	0	Fentanyl patches	90-119	Int
32	21	F	80	L	Oxycodone	60-89	Int
33	21	F	80's	Lower by 2/3	Buprenorphine patch	30-59	UC
34	20	F	60's	H(? S)	Hydromorphone ??	150+??	Int
35	20	M	50's	sl L	Tramadol	30-59	UC
36	26 NE	M	70's	L (or 0)	??Oxycodone and liquid morphine	150+	Int
37	26 NE	F	50s	S	Tramadol	60-89	UC
38	24	F	70's	H sl	Tramadol	0-29	Int
39	27	F	50s	L (or S)	Buprenorphine to Morphine	30-59	UC
40	23 NE	F	60s	0 (or L)	Tramadol to Codeine phosphate	0-29	UC
41	29 NE	F	60s	L sl	Fentanyl patches ??liquid morphine	??	Int

Legend: * H=higher S=Same L=lower 0 = no opioids
** Baseline morphine equivalent bandings: 0-29mg,30-59mg,60-89mg,90-119mg, 120-149mg, 150mg+
*** Allocation: UC- Usual Care, Int-Intervention, ITT-Intention to treat (allocated to Intervention but not exposed to intervention)

Comparison of Usual care and Intervention

Usual care Total 20	7NE 13 Mids	7M 13F	30s to 80s	5H 5S 7L 30	Range of patches tablets and liquid	0-29(8),30-59(4),60-89(3), 90-119(2), 120-149(0), 150+(3)	UC
Intervention Total 20	8 NE 12 Mids	8M 12F	50s to 80s	4H 1S 9L 60	Range of patches tablets and liquid	0-29(5),30-59(5),60-89(4), 90-119(1),120-149(0),150+(4),1 unknown	Int

Commented [NV1]: Do we need this in?

For peer review only

Supplementary material 8 Baseline demographic characteristics of all randomised participants vs interviewed participants

Demographic characteristics	Total N=608	PE Interviewees N=40
**Age (years)**		
N	608	40
Mean (SD)	61.3 (12.9)	64.7 (11.9)
Median (IQR)	62.3 (53.0 – 70.7)	64.1 (58.5, 71.8)
Missing	0	0
**Gender**		
Male	242 (39.8%)	15 (37.5%)
Female	362 (59.5%)	25 (62.5%)
Other	1 (0.2%)	0 (0%)
Prefer not to say	0 (0.0%)	0 (0%)
Missing	3 (0.5%)	0 (0%)
**Ethnicity**		
White	585 (96.2%)	38 (95.0%)
Black Caribbean	6 (1.0%)	1 (2.5%)
Black African	1 (0.2%)	1 (2.5%)
Black Other	1 (0.2%)	0 (0.0%)
Indian	6 (1.0%)	0 (0.0%)
Pakistani	1 (0.2%)	0 (0.0%)
Bangladeshi	0 (0.0%)	0 (0.0%)
Chinese	0 (0.0%)	0 (0.0%)
Prefer not to say	1 (0.2%)	0 (0.0%)
Other	4 (0.7%)	0 (0.0%)
Missing	3 (0.5%)	0 (0.0%)
**Employment status**		
Employed	132 (21.7%)	6 (15.0%)
Unemployed	14 (2.3%)	0 (0.0%)
At school or full time education	1 (0.2%)	0 (0.0%)
At school or part time education	1 (0.2%)	0 (0.0%)
Unable to work due to long term sickness	154 (25.3%)	13 (32.5%)
Looking after home/family	13 (2.1%)	0 (0.0%)
Retired from paid work	270 (44.4%)	21 (52.5%)
Other	20 (3.3%)	0 (0.0%)
Missing	3 (0.5%)	0 (0.0%)
**Age left full time education**		
Did not receive formal education	2 (0.3%)	0 (0.0%)
Age 12 or less	1 (0.2%)	0 (0.0%)
Age 13 to 16	345 (56.7%)	21 (52.5%)
Age 17 to 19	135 (22.2%)	6 (15.0%)
Age 20 or over	109 (17.9%)	12 (30.0%)
Still in full time education	4 (0.7%)	0 (0.0%)
Other	9 (1.5%)	1 (2.5%)
Missing	3 (0.5%)	0 (0.0%)
**How long have you experience pain**		
Less than 1 year	8 (1.3%)	0 (0.0%)
1-5 years	97 (16.0%)	9 (22.5%)
More than 5 years	500 (82.2%)	31 (77.5%)
Missing	3 (0.5%)	0 (0.0%)
**How long have you been taking opioids for your chronic pain**		
Less than 1 year	29 (4.8%)	0 (0.0%)
1-5 years	211 (34.7%)	20 (50.0%)
More than 5 years	365 (60.0%)	20 (50.0%)
Missing	3 (0.5%)	0 (0.0%)

For peer review only

Supplementary material 9 Participant feedback form findings

Questions	Responses			
**Q1 Were the aims of the course made clear?**	Out of 27 responses (4 missing) : 26 Yes, 1 No			
**Q2 What were the three most useful things on this course?**	**Theme** *26 responses (ranked but not all filled in all three).*	**1st**	**2nd**	**3rd**
	Appreciation of the information	8	7	3
	Being in a group – meeting and interacting	8	8	2
	Supported by facilitators	3	3	1
	Lay facilitator input	3	1	0
	Techniques taught in course which were helpful (all different)	3	3	0
	Motivational aspects	1	0	4
	Support of GP	0	0	3
**Q3 What three things would you suggest to make this course better for future participants?**	Out of 24 responses, six suggested to keep it as it is. The overall suggestions from the remainder were disparate. Some wanted more time for discussion (but one wanted shorter sessions due to comfort), more contacts with staff, more meetings especially at end of entire course. Practical issues specific to venue e.g. closeness, parking, one toilet. Two suggested CDs should also be in MP3/4 format. Specific issues: One felt the App advice was unrealistic, one didn't like the meditation, one didn't like the self-help booklet manual, one hadn't used CDs.			

	**Very confident**	**Confident**	**Not very confident**	**Not confident at all**
**Q4: How confident do you feel that the course content will help you personally?**	14	11	1	4
**Q5: How confident do you feel that you will be able to use this in the future?**	16	10	0	1
	**Very good**	**Good**	**Satisfacto ry**	**Poor**
**Q6: Overall were the facilitators?**	23	4	1	0
**Q7: Overall were the handouts?**	15	12	1	0
	**Very useful**	**Useful**	**Not very useful**	**Not useful at all**
**Q8: How did you find the face to face meeting with the nurse?**	22	5	1	0
**Q9: How did you find the telephone calls with the nurse?**	18	9	1	0
**Q10: Overall how useful did you find the whole course?**	22	5	0	1

Q11: Is there anything else you'd like to say? 21 responses to an open comments box. Fifteen positive, 2 negative ('course was a waste of time' and that the approach had not worked for them) and three mixed. Indicative quotes:

"I am now completely off tramadol and despite still going through some nasty withdrawal symptoms I am so glad I had the opportunity to attend the course. I didn't realise how bad the drug is and how ineffective it is for long term chronic pain. I am looking forward to getting my life back."

"I would recommend this course to anyone who has a chance to attend it. It has helped me to come off one of my painkillers, to be more outgoing, to talk about my problems more and given me more confidence when in a group of people."

Supplementary material 10 Summary of intervention attendance data

	Self-management (intervention)
**Group session attendance[1,2]**	
Number randomised to intervention	305
Session 1 only	13 (4.3%)
Session 1 and 2	17 (5.6%)
Session 1 and 3	10 (3.3%)
Session 1, 2 & 3	166 (54.4%)
Attended no sessions	90 (29.5%)
Missing	9 (3.0%)
**Group size at randomisation**	
N	35
Mean (SD)	8.71 (2.9)
Median (IQR)	9 (5,11)
Missing	0 (0%)
**Group size at Session 1[3]**	
N	35
Mean (SD)	6.24 (2.82)
Median (IQR)	7 (3, 8)
Missing	2 (5.7%)
**Face to Face interviews**	
Attended first F2F interview	190 (62.3%)
Attended both F2F interviews	131 (42.9%)
Missing	15 (4.9%)
**Telephone interviews**	
Attended first telephone session	167 (54.8%)
Attended both telephone sessions	152 (49.8%)
Missing	34 (11.1%)
**Compliance**	
Number of participants with full compliance	144 (47%)
Number of participants with at least minimal compliance	190 (62%)
Number with less than minimal compliance	115 (38%)

Legend: [1] 161/305 participants achieved minimal compliance by attending at least Day 1 and the first one-to-one consultation.
[2] 144/305 participants achieved full compliance by attending at least Day 1, 2 & 3, the first one-to-one consultation and 1 telephone call.
[3] 6 participants who attended day 1 attended groups they were not randomised to. This has been summarised by groups they attended, not randomised too.

For peer review only

Supplementary material 11 Fidelity scores of group sessions in percentages

Session	Adherence *(italics 10% check)*			Totals	Competence			Totals
	Early	Mid	Late		Early	Mid	Late	
Day 1 session 2	100	81	25 *(25)*		92	64	0 *(0)*	
Day 1 session 3	94 *(94)*	100	44		90 *(87)* agreed 88	86	42	
Day 1 session 4	100	75	88		70	71	67	
Day1 session 7	72	89	83		80	75	90	
Day 1 session 8	100	88	81		100	60	75	
Day 2 session 13	77	88 *(90)* agreed 89	84		57	100 *(100)*	100	
Day 2 session 14	56 *(66)* agreed 61	100	94		58*(43)* agreed 50	92	90	
Day 2 session 16	79	88	82		50	70	100	
Day 3 session 21	88	100	89		80	100	100	
Day 3 session 22pt 1	93	86	64		70	92	83	
Day3 session 23	90	73	91		100	100	100	
**Average**	**86.72**	**88.09**	**75.00**	**83.27%**	**76.09**	**82.73**	**77**	**78.61%**
**Range**	**61-100**	**73-100**	**25-94**	**25-100%**	**50-100**	**60-100**	**0-100**	**0-100%**
**Median**	**90**	**88**	**83**	**88**	**80**	**86**	**90**	**86**

Supplementary material 12 Fidelity scores of one-to-one nurse consultations

**Timepoint Early**	**Group ID**	**Adherence Score**	**Competence Score**
1st	1	100%	100%
2nd	2	93% *(93%)*	100% *(100%)*
1st	3	61%	67%
2nd	3	86%	83%
1st	4 NE	100%	100%
2nd	4 NE	100%	100%
1st	5 NE	100%	100%
2nd	5 NE	100%	100%
2nd	6	100%	100%
1st	7 NE	67%	100%
1st	8	100%	100%
1st	9 NE	83% *(94%)*94	100% *(83%)*92
2nd	10	88%	83%
2nd	10	100%	100%
**Early averages**		**92.07%**	**94.64%**
**Range**		**61 to 100**	**67 to 100**
**Timepoint Mid**	**Group ID**	**Adherence**	**Competence**
1st	11	100%	100%
1st	12 NE	94%	100%
2nd	12 NE	100%	100%
1st	13	89%	92%
1st	14 NE	61% *(61%)*	50% *(50%)*
1st	15	94%	100%
1st	15	94%	100%
2nd	20	71%	83%
**Mid averages**		**87.87%**	**90.63%**
**Range**		**61 to 100**	**50 to 100**
**Timepoint Late**	**Group ID**	**Adherence Score**	**Competence Score**
1st	21	100%	92%
2nd	21	93%	100%
1st	24	94%	100%
2nd	24	86% *(86%)*	100% *(100%)*
1st	27	89%	83%
**Late averages**		**92.4%**	**95%**
**Range**		**86 to 100**	**83 to 100**
	**Total averages**	**90.78%**	**93.42%**
	**Range**	**61 to 100**	**50 to 100**

Legend: NE - North East region

For peer review only

Supplementary material 13

Baseline change mechanism questions of all randomised participants by treatment group

		Control N=303	Intervention N=305	TOTAL N=608
**1.I want to reduce my opioid use**				
Motivation	Not at all	25 (8%)	21 (7%)	46 (8%)
	By a little	45 (15%)	37 (12%)	82 (14%)
	By Half	36 (12%)	44 (14%)	80 (13%)
	So I only use a little	60 (20%)	95 (31%)	155 (26%)
	So I use no opioids	133 (44%)	102 (33%)	235 (39%)
	Missing	4 (1%)	6 (2%)	10 (2%)
**2.I expect in 4 months' time, I will have reduced my opioid use**				
Expectations	Not at all	45 (15%)	43 (14%)	88 (15%)
	By a little	78 (26%)	82 (27%)	160 (26%)
	By Half	56 (19%)	56 (18%)	112 (18%)
	So I only use a little	67 (22%)	82 (27%)	149 (25%)
	So I use no opioids	50 (17%)	37 (12%)	87 (14%)
	Missing	7 (2%)	5 (2%)	12 (2%)
**3.I am confident I could reduce my opioid use a lot over 4 months**				
Self-efficacy	Not at all confident	90 (30%)	90 (30%)	180 (30%)
	Somewhat confident	70 (23%)	77 (25%)	147 (24%)
	Fairly confident	79 (26%)	79 (26%)	158 (26%)
	Strongly confident	35 (12%)	40 (13%)	75 (12%)
	Completely confident	22 (7%)	15 (5%)	37 (6%)
	Missing	7 (2%)	4 (1%)	11 (2%)
**4.I feel that involvement in this study can help me to reduce my opioid use**				
Perceived credibility of intervention	Not at all	25 (8%)	22 (7%)	47 (8%)
	By a little	76 (25%)	73 (24%)	149 (25%)
	By Half	38 (13%)	46 (15%)	84 (14%)
	So I only use a little	68 (22%)	86 (28%)	154 (25%)
	So I use no opioids	86 (28%)	69 (23%)	155 (26%)
	Missing	10 (3%)	9 (3%)	19 (3%)

For peer review only

Supplementary material 14

Change mechanism question data from 4, 8 and 12 month follow up

**4-month follow-up change mechanism questions of all randomised participants by treatment group**	**Control**	**Intervention**
**I want to reduce my opioid use (If still on opioids)**		
Number still on opioids	194	166
Not at all	20 (10.3%)	18 (10.8%)
By a little	23 (11.9%)	11 (6.6%)
By Half	32 (16.5%)	11 (6.6%)
So I only use a little	29 (14.9%)	31 (18.7%)
So I use no opioids	40 (20.6%)	57 (34.3%)
Missing	50 (25.8%)	38 (22.9%)
**I feel that involvement in this study has helped me to reduce my opioid use**		
Number still on opioids	159	192
Not at all	82 (51.6%)	30 (15.6%)
By a little	43 (27.0%)	24 (12.5%)
By Half	10 (6.3%)	21 (10.9%)
So I only use a little	6 (3.8%)	33 (17.2%)
So I use no opioids	8 (5.0%)	79 (41.1%)
Missing	10 (6.3%)	5 (2.6%)
**8-month follow-up change mechanism questions of all randomised participants by treatment group**	**Control**	**Intervention**
**I want to reduce my opioid use (If still on opioids)**		
Number still on opioids	152	136
Not at all	25 (16.4%)	20 (14.7%)
By a little	25 (16.4%)	16 (11.8%)
By Half	24 (15.8%)	9 (6.6%)
So I only use a little	25 (16.4%)	26 (19.1%)
So I use no opioids	36 (23.7%)	45 (33.1%)
Missing	17 (11.2%)	20 (14.7%)
**I feel that involvement in this study has helped me to reduce my opioid use**		
N	149	181
Not at all	68 (45.6%)	27 (14.9%)
By a little	41 (27.5%)	27 (14.9%)
By Half	16 (10.7%)	20 (11.0%)
So I only use a little	9 (6.0%)	28 (15.5%)
So I use no opioids	12 (8.1%)	74 (40.9%)
Missing	3 (2.0%)	5 (2.8%)
**12-month follow-up change mechanism questions of all randomised participants by treatment group**	**Control**	**Intervention**
**I want to reduce my opioid use (If still on opioids)**		
Number still on opioids	193	160
Not at all	20 (10.4%)	18 (11.3%)
By a little	22 (11.4%)	10 (6.3%)
By Half	27 (14.0%)	15 (9.4%)
So I only use a little	36 (18.7%)	32 (20.0%)
So I use no opioids	38 (19.7%)	49 (30.6%)
Missing	50 (25.9%)	36 (22.5%)
**I feel that involvement in this study has helped me to reduce my opioid use**		
N	160	188
Not at all	73 (45.6%)	26 (13.8%)
By a little	39 (24.4%)	25 (13.3%)
By Half	17 (10.6%)	18 (9.6%)
So I only use a little	10 (6.3%)	44 (23.4%)

For peer review only

So I use no opioids	18 (11.3%)	72 (38.3%)
Missing	3 (1.9%)	3 (1.6%)

For peer review only

Supplementary material 15

Involvement in study reducing opioid use at 4, 8 and 12 months allocation comparison

**I feel that involvement in this study has helped me to reduce my opioid use:** Not at all, by a little, by half, so I only use a little, so I use no opioids						
**Grouping into 3 bands**	**Control 4month follow up**	**Intervention 4month follow up**	**Control 8month follow up**	**Intervention 8month follow up**	**Control 12month follow up**	**Intervention 12month follow-up**
N	159	192	149	181	160	188
Opioids reduced not at all + by a little	125 (79%)	54 (28%)	109 (73%)	54 (30%)	112 (70%)	51 (26%)
By half	10 (6%)	21 (11%)	16 (11%)	20 (11%)	17 (11%)	18 (10%)
Opioids reduced so I only use a little + so I use none	14 (9%)	112 (58%)	21 (14%)	102 (56%)	28 (18%)	116 (62%)
Missing	10 (6%)	5 (3%)	3 (2%)	5 (3%)	3 (2%)	3 (2%)

For peer review only
